# Prevention of infections and fever to improve outcome in older patients with acute stroke (PRECIOUS): a randomised, open, phase III, multifactorial, clinical trial with blinded outcome assessment

**DOI:** 10.1016/j.lanepe.2023.100782

**Published:** 2023-12-01

**Authors:** Jeroen C. de Jonge, Wouter M. Sluis, Hendrik Reinink, Philip M. Bath, Lisa J. Woodhouse, Berber Zweedijk, Diederik van de Beek, Anne Hege Aamodt, Iris Alpers, Alfonso Ciccone, Laszlo Csiba, Jacques Demotes, Janika Kõrv, Iwona Kurkowska-Jastrzebska, Jesse Dawson, Malcolm R. Macleod, George Ntaios, Sven Poli, Haralampos Milionis, Stefano Ricci, Silvia Cenciarelli, Paolo Candelaresi, Sebastiaan FTM. de Bruijn, Rohan Pathansali, Kailash Krishnan, Brian Clarke, Götz Thomalla, H Bart van der Worp, the PRECIOUS investigators, Henk Kerkhoff, Marieke JH. Wermer, Korné Jellema, Vincent IH. Kwa, Ben P. Jansen, Tobien AHCML. Schreuder, Sanne M. Zinkstok, Walid Moudrous, Katrin Antsov, Katrin Gross-Paju, Inga Kalju, Gerhard F. Hamann, Michael Rosenkranz, Christoph Gumbinger, Georg Royl, Susanne Müller, Sophie Vassilopoulou, Athanasios D. Protogerou, Efstathios Manios, Dániel Bereczki, Gábor Jakab, Ferenc Nagy, András Folyovich, László Szapáry, Nicola Gilberti, Enrico Righetti, Pietro Bassi, Simona Marcheselli, Alessia Giossi, Anne G. Holtan, Sameer Maini, Waldemar Fryze, Waldemar Brola, Piotr Sobolewski, Marta Bilik, Ruth Davies, Anand Nair, Dipankar Dutta, Martin Cooper, Khalid Rashed, Louise Shaw, Johann R. Selvarajah, Jessica Redgrave, Mary Joan MacLeod, Philip Clatworthy, Vasileios Papavasileiou, Vera Cvoro, Omid Halse, Usman Ghani

**Affiliations:** a Department of Neurology and Neurosurgery, Brain Center, University Medical Center Utrecht, Heidelberglaan 100, 3584 CX, Utrecht, the Netherlands; b Stroke Trials Unit, Mental Health & Clinical Neurosciences, School of Medicine, University of Nottingham, Nottingham, United Kingdom; c Department of Neurology, Amsterdam University Medical Centers, Location AMC, Amsterdam Neuroscience, Amsterdam, the Netherlands; d Department of Neurology, Oslo University Hospital, Oslo, Norway; e Clinical Trial Center North, University Medical Center Hamburg-Eppendorf, Hamburg, Germany; f Department of Neurology and Stroke Unit, ASST di Mantova, Mantua, Italy; g Department of Neurology, University of Debrecen, Debrecen, Hungary; h European Clinical Research Infrastructure Network & Institut National de la Santé et de la Recherche Médicale, Paris, France; i Department of Neurology and Neurosurgery, University of Tartu, Tartu, Estonia; j 2nd Department of Neurology, Institute of Psychiatry and Neurology, Warsaw, Poland; k Institute of Cardiovascular and Medical Sciences, University of Glasgow, Glasgow, United Kingdom; l Centre for Clinical Brain Sciences, University of Edinburgh, Edinburgh, United Kingdom; m Department of Internal Medicine, School of Health Sciences, University of Thessaly, Larissa, Greece; n Department of Neurology & Stroke, University of Tübingen, Tübingen, Germany; o Hertie Institute for Clinical Brain Research, University of Tübingen, Tübingen, Germany; p Department of Internal Medicine, Faculty of Medicine, School of Health Sciences, University of Ioannina, Ioannina, Greece; q Stroke Center - Neurology, Città Di Castello Hospital and Gubbio-Gualdo Tadino Hospital, Città di Castello, Italy; r Neurology and Stroke Unit, AORN “Antonio Cardarelli”, Naples, Italy; s Department of Neurology, Haga Hospital, the Netherlands; t Department of Stroke Medicine, King’s College Hospital NHS Foundation Trust, London, United Kingdom; u Stroke, Department of Acute Medicine, Nottingham University Hospitals NHS Trust, Nottingham, United Kingdom; v Department of Neurology, St George’s University Hospitals NHS Foundation Trust, London, United Kingdom; w Department of Neurology, University Medical Center Hamburg-Eppendorf, Hamburg, Germany; x Norway and Department of Neuromedicine and Movement Science, Norwegian University of Science and Technology, Trondheim, Norway

**Keywords:** Stroke, Ischaemic stroke, Intracerebral haemorrhage, Fever, Infection, Pneumonia

## Abstract

**Background:**

Infections and fever after stroke are associated with poor functional outcome or death. We assessed whether prophylactic treatment with anti-emetic, antibiotic, or antipyretic medication would improve functional outcome in older patients with acute stroke.

**Methods:**

We conducted an international, 2∗2∗2-factorial, randomised, controlled, open-label trial with blinded outcome assessment in patients aged 66 years or older with acute ischaemic stroke or intracerebral haemorrhage and a score on the National Institutes of Health Stroke Scale ≥ 6. Patients were randomly allocated (1:1) to metoclopramide (oral, rectal, or intravenous; 10 mg thrice daily) vs. no metoclopramide, ceftriaxone (intravenous; 2000 mg once daily) vs. no ceftriaxone, and paracetamol (oral, rectal, or intravenous; 1000 mg four times daily) vs. no paracetamol, started within 24 h after symptom onset and continued for four days. All participants received standard of care. The target sample size was 3800 patients. The primary outcome was the score on the modified Rankin Scale (mRS) at 90 days analysed with ordinal logistic regression and reported as an adjusted common odds ratio (an acOR < 1 suggests benefit and an acOR > 1 harm). This trial is registered (ISRCTN82217627).

**Findings:**

From April 2016 through June 2022, 1493 patients from 67 European sites were randomised to metoclopramide (n = 704) or no metoclopramide (n = 709), ceftriaxone (n = 594) or no ceftriaxone (n = 482), and paracetamol (n = 706) or no paracetamol (n = 739), of whom 1471 were included in the intention-to-treat analysis. Prophylactic use of study medication did not significantly alter the primary outcome at 90 days: metoclopramide vs. no metoclopramide (adjusted common odds ratio [acOR], 1.01; 95% CI 0.81–1.25), ceftriaxone vs. no ceftriaxone (acOR 0.99; 95% CI 0.77–1.27), paracetamol vs. no paracetamol (acOR 1.19; 95% CI 0.96–1.47). The study drugs were safe and not associated with an increased incidence of serious adverse events.

**Interpretation:**

We observed no sign of benefit of prophylactic use of metoclopramide, ceftriaxone, or paracetamol during four days in older patients with a moderately severe to severe acute stroke.

**Funding:**

This project has received funding from the European Union’s Horizon 2020 research and innovation programme under grant agreement No: 634809.

**Research in context**

**Evidence before this study**

On 1-2-2023, we searched PubMed, Embase and Clinicaltrial.gov using the terms ‘stroke’, ‘antibiotic,’ ‘anti-emetic,’ ‘antipyretic,’ ‘randomised controlled trial’, and comparable terms. No language restrictions were used. Effects of the prophylactic use of antibiotics in patients with acute ischaemic stroke or intracerebral haemorrhage have been studied in 14 randomised clinical trials, of which two were large phase 3 trials. A meta-analysis of 9 of these trials (n = 4197) showed a reduction in the frequency of any infection from 20% to 13%, but no reduction in the rate of pneumonia nor an improvement of functional outcome.

Paracetamol has previously been tested in four phase II and two phase III trials. In the largest study PAIS, paracetamol reduced the number of patients with a subfebrile temperature or fever after 24 h by 50% and was associated with a tendency towards improvement in functional outcome. In a post-hoc analysis of patients with a body temperature of 37–39 °C, paracetamol was associated with improved outcome, but this could not be replicated in a prematurely terminated clinical trial of paracetamol in patients with an increased body temperature.

Metoclopramide has been studied in one phase II trial in patients with stroke fed via a nasogastric tube. Treatment for 21 days led to a reduction in the rate of pneumonia from 87% to 27%, but functional outcomes were not reported. The effects of the prophylactic use of metoclopramide on the rate of pneumonia and on functional outcome are currently studied in MAPS-2 (ISRCTN14124645).

**Added value of this study**

PRECIOUS showed no sign of benefit of the prophylactic use of metoclopramide, ceftriaxone, paracetamol, or any combination of these, in patients at high risk of developing infections after stroke, as compared to standard care. Updated meta-analysis of all studies assessing the effects of prophylactic antibiotics or antipyretic drugs on functional outcome shows no benefit of prophylactic antibiotics in a total of 5193 patients, and no benefit of prophylactic antipyretic drugs in a total of 3281 patients [Fig. 4].

**Implications of all the available evidence**

Prophylactic anti-emetic, antibiotic, or antipyretic medication should not be advised in patients with acute stroke, even if they are at high risk of developing an infection. The substantial number of patients who were treated with anti-emetics, antibiotics, or antipyretics when not allocated to a study drug suggests that clinicians recognise aspiration and infection early and that the prophylactic use of these medications does not add to organised (stroke-unit) care. Which individual strategies drive the benefit of care on a stroke unit remains uncertain.

## Introduction

Infection and fever are common complications in the first days after stroke, occurring each in about one third of patients.^1^^,^^2^ The risk of developing an infection is greater in older patients and those with more severe stroke.^3^ In the acute phase of stroke, increased body temperature, infections in general, and pneumonia in particular are independently associated with a greater risk of death or dependency.^1^^,^^2^ Animal studies of ischaemic stroke have suggested that the relation between higher body temperatures and poor outcome is at least in part causal.^4^

Monitoring of body temperature and prevention of aspiration pneumonia are important components of stroke unit care,^5^ but it is uncertain whether these contribute to the observed benefit of such organised care.^6^ In a cluster-randomised trial, protocol-based management of fever, hyperglycaemia, and swallowing during the first three days of admission to an acute stroke unit was associated with a reduction in the risk of death or dependency at 90 days.^7^

For strategies addressing a single post-stroke complication the evidence of benefit is much less convincing. Prophylactic treatment with the antiemetic and prokinetic drug metoclopramide reduced the risk of pneumonia in a randomised trial of 60 stroke patients fed via a nasogastric tube, but functional outcomes of these patients were not reported.^8^ High-dose paracetamol started in the first 12 h after stroke onset in the PAIS trial halved the risk of subfebrile temperatures or fever 24 h later, but did not improve functional outcome in the overall population.^9^^,^^25^ However, there was a tendency towards improved functional outcome and a statistically significant benefit was observed in patients with a baseline body temperature of at least 37.0 °C. In a systematic review of nine trials, preventive antibiotic therapy in patients with acute stroke did not improve functional outcome and, remarkably, also did not reduce the occurrence of pneumonia.^10^ However, a post-hoc analysis of the PASS trial suggested that prophylactic ceftriaxone may reduce the rate of pneumonia in patients of high age and with more severe neurological deficit, who are at the greatest risk of pneumonia.^11^

In the current PREvention of Complications to Improve OUtcome in elderly patients with acute Stroke (PRECIOUS) trial we assessed whether a pharmacological strategy to prevent infections or fever with metoclopramide, ceftriaxone, paracetamol, or any combination of these, in the first four days after stroke onset, improves outcome in older patients with a moderately severe to severe acute stroke.

## Methods

### Study design and participants

PRECIOUS was an investigator-initiated, multi-centre, multi-factorial, open-label, randomised controlled clinical trial with blinded outcome assessment of the preventive use of metoclopramide vs. no metoclopramide, ceftriaxone vs. no ceftriaxone, and paracetamol vs. no paracetamol, in older patients with acute stroke across 82 academic and non-academic hospitals in nine European countries (Estonia, Germany, Greece, Hungary, Italy, the Netherlands, Norway, Poland, United Kingdom). The trial protocol and the statistical analysis plan have been published previously and can be found in the Supplementary Material.^12^^,^^13^ The trial population consisted of patients aged 66 years or older who were hospitalised with moderately severe to severe acute ischaemic stroke or intracerebral haemorrhage, defined as a score on the National Institutes of Health Stroke Scale (NIHSS) of 6 or higher. Trial treatment initiation originally had to be possible within 12 h of stroke onset, but this was extended to 24 h after an approved amendment to the study protocol in December 2017 in an attempt to increase recruitment. Patients were excluded if they had an active infection requiring antibiotic treatment or if they had a pre-stroke score on the modified Rankin Scale (mRS) of 4 or higher. Detailed inclusion and exclusion criteria are listed in the study protocol. Patients, their legal representatives or independent physicians provided written informed consent. The trial was approved by the central medical ethics committee of the University Medical Center Utrecht on 3 February 2016 and by national or local research ethics committees in all participating countries.

### Randomisation and masking

Patients were randomly allocated in a 2∗2∗2 factorial design (1:1) to metoclopramide vs. no metoclopramide, ceftriaxone vs. no ceftriaxone, and paracetamol vs. no paracetamol. Treatment allocation was open and based on minimisation through a web-based allocation service. Investigators had the opportunity to omit a single randomisation stratum before randomisation in a specific patient (for example in case of an allergy to or a clinical indication for one of the study drugs) or for all patients at their study site (for example because of concerns about the prophylactic use of ceftriaxone). In these cases, randomisation was limited to the other two stratums. Treatment allocation was stratified by country and included the following minimisation factors: age (66–75 years vs. > 75 years); sex (male vs. female); stroke type (ischaemic stroke vs. intracerebral haemorrhage); stroke severity (NIHSS 6–12 vs. > 12); and diabetes mellitus (yes vs. no). In January 2020, after inclusion of 865 patients, it became apparent that the web-based allocation service mistakenly also used study site as a stratification factor, which was corrected after discovery.

During the final follow-up visit at 90 (±14) days after inclusion, an outcome interview by a trained investigator was recorded using a digital video camera. When a video recording of the patient was not possible, a video recording of an interview with a nurse or another caregiver, or an audiotape with a full description of the medical condition of the patient, was obtained. For each video, the score on the mRS was assessed by three independent raters blinded to treatment allocation.

### Procedures

Study treatment consisted of metoclopramide (oral, rectal, or intravenous; 10 mg thrice daily) or no metoclopramide, ceftriaxone (intravenous; 2000 mg once daily) or no ceftriaxone, and paracetamol (oral, rectal, or intravenous; 1000 mg four times daily) or no paracetamol, started within 24 h after symptom onset and continued for four days. All participants received standard of care as determined by each site, including reperfusion therapies. In patients with moderate to severe renal impairment (estimated glomerular filtration rate (eGFR) 15–60 ml/min) or with severe hepatic impairment (liver cirrhosis), the dose of metoclopramide was reduced to 5 mg thrice daily, and in patients with end-stage renal disease (eGFR ≤ 15 ml/min) to 2.5 mg thrice daily. Study treatment was started within 24 h after stroke onset and continued for four days or until discharge, if earlier. Treating physicians were allowed to start any antiemetic, antibiotic, or antipyretic drug in patients in any treatment group if clinically indicated. An overview of the data collected at baseline, during hospital admission, and at 90 days follow-up is provided in the protocol paper and in the study protocol in the Supplementary Material.^12^

### Outcomes

The primary outcome was the score on the mRS at 90 days (±14 days). A median mRS score was calculated for each patient from the three mRS scores obtained through centralised adjudication. If no video or audio recording was obtained, the mRS score by the local investigator at 90 days follow-up was used. Secondary outcomes included infections, bacterial resistance to third generation cephalosporins (detected as part of routine clinical care), antimicrobial use (converted to units of defined daily doses according to the classification of the WHO Anatomical Therapeutic Chemical Classification System with Defined Daily Doses Index), and serious adverse events (SAEs), all in the first seven days after randomisation. Infections are reported both as diagnosed by the treating physician and as adjudicated by an expert panel using the CDC and PISCES criteria.^14^^,^^15^ SAEs were collected up to and including day 90 if these were believed to be possibly related to the study medication. Death, unfavourable functional outcome (defined as a score on the mRS of 3–6), disability (assessed with the Barthel Index (BI)), cognition (assessed with the score on the Montreal Cognitive Assessment (MoCA)), and quality of life (assessed with the EuroQol 5D-5L) at 90 days, home time,^16^ and patient location over the first 90 days were other secondary outcomes.

### Statistical analysis

A statistical analysis plan has been published previously.^13^ A total sample size of 3800 patients was estimated to yield 90% power to detect a statistically significant difference in the proportion of patients with mRS 0 to 2 at 90 days, assuming an effect that leads to a 5% absolute increase (from 36% to 41%) in the cumulative proportion of patients with mRS 0 to 2 in any intervention group compared with controls. The use of a binary choice for mRS in sample size estimation is conservative for subsequent analysis of the ordinal mRS.^17^ Where the published protocol and statistical analysis plan^12^^,^^13^ diverge in describing plans for analysis, the latter takes precedence since more recent. The primary outcome was analysed with multivariable ordinal logistic regression to determine an adjusted common odds ratio (acOR) with 95% confidence interval (95% CI); an acOR < 1 suggests benefit and acOR > 1 potential harm. A likelihood ratio test was used to assess the proportional odds assumption. Three separate primary analyses were performed, one for each intervention vs. their respective controls (e.g. metoclopramide vs. non-metoclopramide). The control group for each intervention consisted of patients who were not randomised to that intervention (both patients in the usual care group and patients in the other study drug groups) and excluded patients in whom that study drug was to be omitted. Analyses were adjusted for stratification and minimisation factors, other baseline prognostic factors (reperfusion treatment, time from onset to randomisation, pre-stroke mRS, atrial fibrillation), and treatment allocation to the other two strata of the trial. The statistical analyses were performed according to the intention-to-treat principle, including all randomised patients with a valid score on the mRS at 90 ± 14 days. Comparison of the effect of the three intervention groups vs. their respective controls on the primary outcome was also performed in pre-specified subgroups.^13^ Although the study was not powered to detect interactions between the three interventions, such interactions were investigated in secondary analyses. We did not include higher–level interactions, e.g. ceftraxione x paracetamol for the comparison of metoclopramide vs. no metoclopramide, because of the complexity of such models in the presence of omitted data. For the secondary outcomes, binary logistic regression was used for binary outcomes, Cox proportional hazards regression was used for time to events (e.g. death), and multivariable linear regression was used for continuous outcomes. Missing outcome data were not imputed for the primary analyses but a sensitivity analysis with imputed outcome data was done. For the secondary outcome measures BI, MoCA, EQ-5D-5L, and EQ-VAS, patients who had died were assigned a value one unit worse than any living value.^18^ Hence, deceased patients could not be given a score similar to the worst score of patients who were alive. This approach ensured that all patients were included in the analysis. We also performed analyses for these outcomes in patients who were alive at 90 days. We made no adjustments for multiplicity of testing since all secondary analyses were hypothesis-generating and designed to support the primary analyses. An independent data safety monitoring board conducted unblinded interim analyses to assess the safety and efficacy of the trial. All analyses were two-tailed, and a *p* value of < 0.05 denotes statistical significance. All data was analysed using SAS version 9.4 (SAS institute, Cary, NC, United States). This trial is registered with the International Standard Randomised Controlled Trial Number, ISRCTN82217627.

### Role of the funding source

The funders of the study had no role in study design, planning, data analysis, data interpretation, or writing of the report.

## Results

**Fig. 1:**
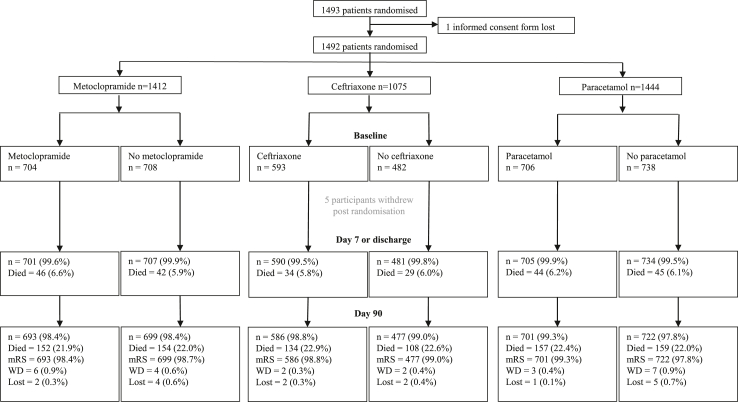
**Trial profile.** mRS indicates modified Rankin Scale; WD, withdrawn; lost, lost to follow-up.

On 30 June 2022, inclusion in the trial was terminated prematurely because of cessation of funding. From April 2016 through June 2022, 1493 patients were enrolled at 67 of the 82 activated study sites and were randomly assigned to one of the study groups. During the COVID-19 pandemic, recruitment was suspended at a varying number of study sites, subject to local regulation. Sixteen study sites omitted ceftriaxone for all of their patients. After excluding one patient whose informed consent form was lost, five patients who withdrew consent immediately after randomisation, ten patients who withdrew consent during the course of follow-up and six patients (0.4%) who were lost to follow-up, 1471 patients (98.5%) were included in the intention-to-treat analysis. These patients were randomised to metoclopramide (n = 693) or no metoclopramide (n = 699), ceftriaxone (n = 586) or no ceftriaxone (n = 477), and paracetamol (n = 701) or no paracetamol (n = 722; Fig. 1). In 1118 (97.6%) of the 1146 patients who were alive at 90-days follow-up, a median mRS by centralised adjudication was available. For the other 28 patients the score as determined by the local investigator was used. Protocol violations with regard to eligibility are listed in Supplementary Table S1.

The median time from symptom onset to randomisation was 14.6 h (IQR 7.8–20.5). The mean age of the patients was 79.7 years (SD 7.7), 741 (49.7%) were female and the median NIHSS score was 12 (IQR 8–17). The final clinical diagnosis was ischaemic stroke in 1263 (84.9%) patients, intracerebral haemorrhage in 204 (13.7%), and a TIA or stroke mimic in 21 (1.4%). Mean baseline temperature was 36.5 °C (SD 0.5) in all treatment groups. Of all patients with ischaemic stroke, 593 (47%) were treated with intravenous thrombolysis (IVT) and 305 (24.2%) with endovascular thrombectomy, of whom 177 (13.8%) received both revascularisation treatments. Demographic and clinical characteristics were similar between treatment groups (Table 1). Sixty eight patients (9.6%) randomised to ‘no metoclopramide’ received any anti-emetic medication in the first seven days, 151 (31.5%) of those randomised to ‘no ceftriaxone’ received any antibiotic, and 326 (44%) of those randomised to ‘no paracetamol’ received any antipyretic (Supplementary Table S2).

**Table 1:** Baseline characteristics.

	n	All	Metoclopramide		Ceftriaxone		Paracetamol	
			Yes	None	Yes	None	Yes	None
**Patients randomised**		1492^a^	704	708	593	482	706	738
Age	1492	79.7 (7.7)	79.7 (7.8)	79.6 (7.8)	79.6 (8.1)	79.6 (7.8)	79.7 (7.7)	79.9 (7.7)
Sex, male (%)	1492	751 (50.3)	345 (49)	368 (52)	308 (51.9)	229 (47.5)	355 (50.3)	368 (49.9)
Pre-stroke mRS, median [IQR]	1490	0 [0,2]	0 [0,2]	0 [0,2]	0 [0,2]	0 [0,2]	0 [0,2]	0 [0,2]
Ethnicity, White (%)	1490	1435 (96.3)	677 (96.3)	680 (96.2)	575 (97.1)	474 (98.5)	672 (95.2)	717 (97.4)
**Medical history (%)**								
Atrial fibrillation (%)	1490	441 (29.6)	199 (28.3)	208 (29.4)	168 (28.4)	152 (31.6)	220 (31.2)	210 (28.5)
Hypercholesterolaemia (%)	1490	528 (35.4)	250 (35.6)	253 (35.8)	214 (36.1)	175 (36.4)	266 (37.7)	251 (34.1)
Hypertension (%)	1490	1084 (72.8)	514 (73.1)	515 (72.8)	431 (72.8)	375 (78)	500 (70.8)	549 (74.6)
Diabetes mellitus (%)	1492	321 (21.5)	159 (22.6)	146 (20.6)	131 (22.1)	113 (23.4)	157 (22.2)	152 (20.6)
Obstructive pulmonary disease (%)	1490	114 (7.7)	68 (9.7)	41 (5.8)	51 (8.6)	34 (7.1)	45 (6.4)	67 (9.1)
Previous stroke (%)	1490	294 (19.7)	139 (19.8)	137 (19.4)	116 (19.6)	97 (20.2)	141 (20)	137 (18.6)
Immunocompromised (%)	1490	15 (1)	9 (1.3)	6 (0.8)	6 (1)	5 (1)	6 (0.8)	8 (1.1)
Smoking status (%)								
Current	1492	152 (10.2)	72 (10.2)	74 (10.5)	79 (13.3)	47 (9.8)	68 (9.6)	76 (10.3)
Ever	1492	343 (23)	167 (23.7)	160 (22.6)	119 (20.1)	98 (20.3)	165 (23.4)	166 (22.5)
Never/unknown	1492	997 (66.8)	465 (66.1)	474 (66.9)	395 (66.6)	337 (69.9)	473 (67)	496 (67.2)
Pre-stroke feeding status (%)	.							
Normal	1490	1482 (99.5)	699 (99.4)	704 (99.6)	588 (99.3)	480 (99.8)	704 (99.7)	731 (99.3)
Oral softened food or fluids only	1490	8 (0.5)	4 (0.6)	3 (0.4)	4 (0.7)	1 (0.2)	2 (0.3)	5 (0.7)
Nasogastric tube	1490	0	0	0	0	0	0	0
Percutaneous endoscopic gastrostomy (PEG)	1490	0	0	0	0	0	0	0
Intravenous only	1490	0	0	0	0	0	0	0
Use of drugs 3 days before randomisation (%)								
Antipyretic inc. paracetamol	1490	129 (8.7)	67 (9.5)	57 (8.1)	42 (7.1)	40 (8.3)	66 (9.3)	56 (7.6)
Antiemetic inc. metoclopramide	1490	59 (4)	34 (4.8)	23 (3.3)	20 (3.4)	14 (2.9)	32 (4.5)	26 (3.5)
Antibiotics inc. ceftriaxone	1490	19 (1.3)	10 (1.4)	9 (1.3)	5 (0.8)	5 (1)	9 (1.3)	10 (1.4)
Time from onset to randomisation (hours)	1490	14.6 [7.8, 20.5]	14.8 [7.6, 20.4]	14.4 [7.8, 20.8]	13.4 [7.5, 19.9]	14.3 [8.6, 20.5]	14.8 [7.8, 20.7]	14.5 [7.7, 20.3]
Stroke type (%)								
Ischaemic stroke	1488	1263 (84.9)	590 (84)	608 (86)	509 (86.3)	420 (87.3)	582 (82.4)	644 (87.7)
Intracerebral haemorrhage	1488	204 (13.7)	99 (14.1)	95 (13.4)	75 (12.7)	53 (11)	113 (16)	80 (10.9)
Other diagnosis	1488	21 (1.4)	13 (1.9)	4 (0.6)	6 (1)	8 (1.7)	11 (1.6)	10 (1.4)
NIHSS total score (/42)	1492	12 [8,17]	11 [8,17]	12 [8,17]	11 [8,17]	11 [8,16]	12 [8,17]	12 [8,17]
Systolic blood pressure (mmHg)	1483	152.8 (26)	153.4 (25.6)	152.2 (26.3)	151 (25.7)	151.9 (26.3)	151.7 (26.4)	153.8 (25.5)
Diastolic blood pressure (mmHg))	1483	80.6 (16.8)	81 (17.1)	80.5 (16.5)	79.5 (16.6)	80.3 (17)	80.5 (16.7)	80.6 (16.7)
Heart rate (bpm)	1470	78.1 (17.8)	78.3 (18.4)	77.7 (17.2)	77.6 (17.5)	79.1 (18)	78 (18)	77.8 (17.7)
Temperature (°C)	1380	36.5 (0.5)	36.5 (0.5)	36.5 (0.5)	36.5 (0.5)	36.5 (0.6)	36.5 (0.5)	36.5 (0.5)
Acute stroke treatment (%)								
Intravenous thrombolysis	1282	598 (46.6)	281 (46.8)	287 (46.9)	253 (49.2)	193 (45.1)	282 (47.6)	295 (45.2)
Mechanical thrombectomy	1282	305 (23.8)	149 (24.8)	133 (21.7)	149 (29)	107 (25)	138 (23.3)	161 (24.7)

Data are n (%), mean (SD) or median [IQR].

inc indicates including; mRS, modified Rankin Scale; NIHSS, National Institutes of Health Stroke scale; BP, blood pressure.

a Note that the Medical Ethics Committee of UMC Utrecht did not allow to use any data of the patient whose consent form was lost.

**Fig. 2:**
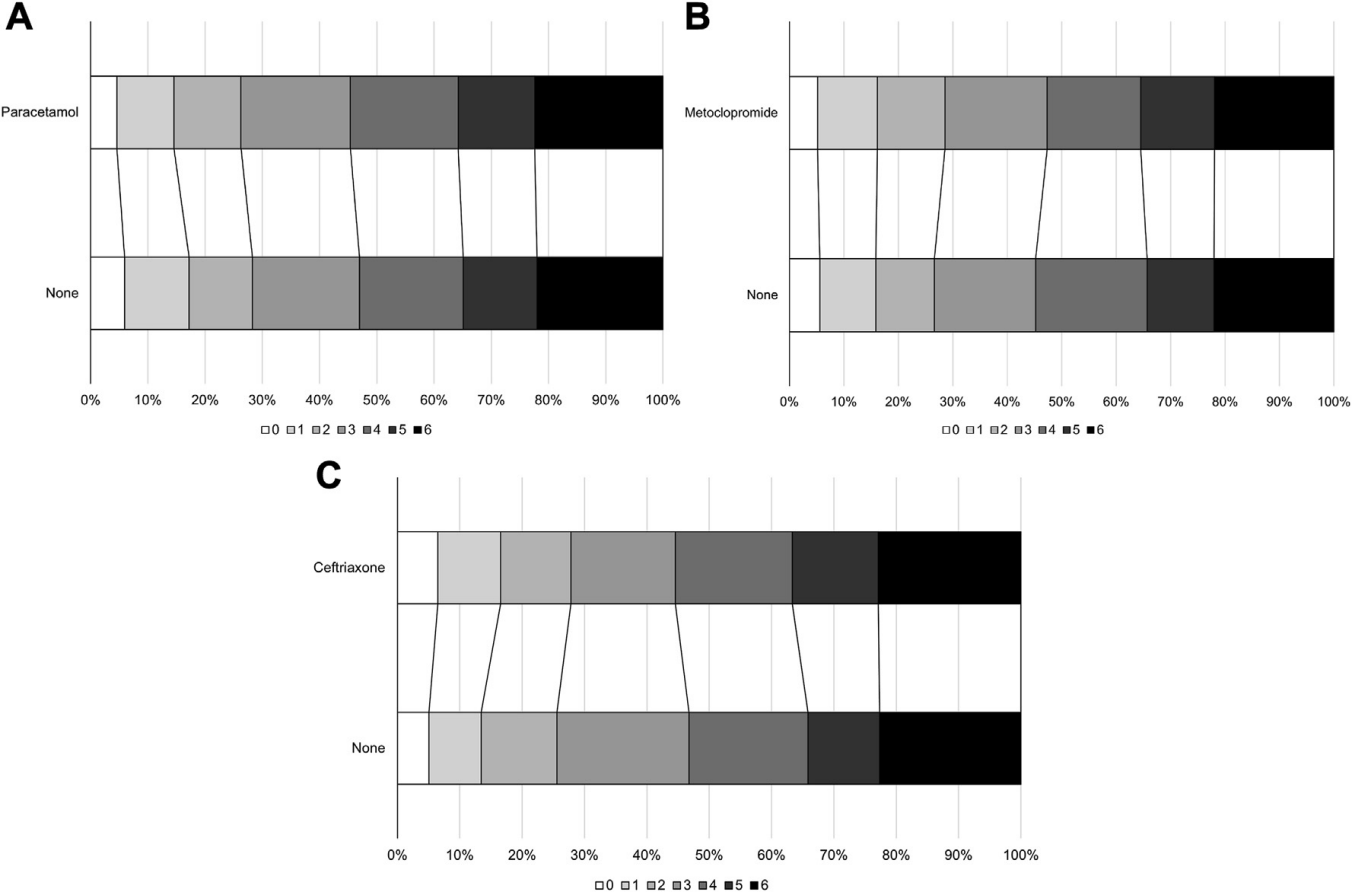
**Distribution of scores on the modified Rankin Scale at 90 days for each treatment stratum.** (A) Patients allocated to paracetamol vs. patients allocated to no paracetamol; (B) Patients allocated to metoclopramide vs. patients allocated to no metoclopramide; (C) Patients allocated to ceftriaxone vs. patients allocated to no ceftriaxone.

When assessing the assumption of common odds, the likelihood ratio test found no deviation from the assumption for any of the three comparisons (Table 2). Prophylactic use of metoclopramide, ceftriaxone, or paracetamol was not associated with a shift in the distribution of the scores on the mRS towards a better functional outcome at 90 days (metoclopramide: acOR, 1.01; 95% CI 0.81–1.25; *p* = 0.94; ceftriaxone: acOR, 0.99; 95% CI 0.77–1.27; *p* = 0.93), with higher ORs indicating poorer outcomes. Although not statistically significant, there was a tendency to a worse outcome with paracetamol vs. no paracetamol (acOR, 1.19; 95% CI 0.96–1.47; *p* = 0.12) (Table 2; Fig. 2A–C). None of the treatments had an effect on the number of patients who had an unfavourable outcome (mRS ≥ 3) or died, nor were there differences between treatment groups for any of the other secondary outcomes (Table 3 and Supplementary Fig. S1 A–C). No combination of study drugs was associated with a better functional outcome or survival at 90 days (Supplementary Tables S2 and S3).

**Table 2:** Primary and secondary outcomes.

	N	Metoclopramide				Ceftriaxone				Paracetamol			
		Yes	None	DIM/OR (95% CI)	*p*	Yes	None	DIM/OR (95% CI)	*p*	Yes	None	DIM/OR (95% CI)	*p*
**Primary outcome**^a^													
mRS, median [IQR]	1471	4 [2,5]	4 [2,5]	1.01 (0.81, 1.25)	0.94	4 [2,5]	4 [2,5]	0.99 (0.77, 1.27)	0.92	4 [2,5]	4 [2,5]	1.19 (0.96, 1.47)	0.12
**Sensitivity analysis**													
mRS, unadjusted	1471	4 [2,5]	4 [2,5]	0.97 (0.80, 1.16)	0.73	4 [2,5]	4 [2,5]	1.00 (0.81, 1.24)	0.97	4 [2,5]	4 [2,5]	1.08 (0.90, 1.29)	0.42
mRS, imputed	1492	4 [2,5]	4 [2,5]	1.00 (0.81, 1.25)	0.98	4 [2,5]	4 [2,5]	0.99 (0.77, 1.28)	0.96	4 [2,5]	4 [2,5]	1.19 (0.97, 1.48)	0.10
mRS, mean	1471	3.6 (1.8)	3.6 (1.8)	−0.02 (−0.21, 0.16)	0.83	3.6 (1.9)	3.7 (1.8)	−0.06 (−0.27, 0.15)	0.59	3.7 (1.8)	3.6 (1.9)	0.13 (−0.05, 0.32)	0.15
mRS > 2 (%)	1471	495 (71.4)	513 (73.4)	0.84 (0.62, 1.14)	0.27	423 (72.2)	355 (74.4)	0.91 (0.64, 1.29)	0.58	517 (73.8)	518 (71.7)	1.15 (0.85, 1.55)	0.35
**By diagnosis**													
Ischaemic stroke	1250	4 [2,5]	4 [2,5]	1.00 (0.80, 1.25)	1.00	4 [2,5]	4 [2,5]	0.97 (0.75, 1.24)	0.78	4 [2,5]	4 [2,5]	1.18 (0.95, 1.47)	0.13
Intracerebral haemorrhage	201	4 [3,5]	4 [3,5]	0.92 (0.53, 1.62)	0.78	4 [3,5]	4 [3,5]	1.09 (0.53, 2.25)	0.81	4 [3,5]	4 [3,5]	0.82 (0.47, 1.43)	0.49
Other diagnosis	20	2 [2,3]	2.5 [1.5, 3.5]	31.53 (0.01, 78,349)	0.39	3 [2,6]	2.5 [2, 3.5]			3 [2,6]	2 [2,3]	1707.8 (0.34, 8.59E6)	0.087
**Secondary outcomes**													
Death (%)	1471	152 (21.9)	154 (22)	1.04 (0.80, 1.36)	0.76	134 (22.9)	108 (22.6)	1.06 (0.78, 1.43)	0.71	157 (22.4)	159 (22)	1.13 (0.87, 1.46)	0.36
**Patient location**				1.05 (0.83, 1.33)	0.67			1.02 (0.78, 1.34)	0.89			1.32 (1.05, 1.66)	0.018
Home (%)	1464	276 (39.9)	289 (41.6)			220 (38.1)	178 (37.7)			267 (38.6)	315 (43.5)		
Nursing home (%)	1464	53 (7.7)	52 (7.5)			45 (7.8)	35 (7.4)			52 (7.5)	59 (8.1)		
Rehabilitation service (%)	1464	128 (18.5)	137 (19.7)			126 (21.8)	104 (22)			137 (19.8)	127 (17.5)		
Hospital (%)	1464	72 (10.4)	59 (8.5)			47 (8.1)	43 (9.1)			72 (10.4)	58 (8)		
Other (%)	1464	10 (1.4)	4 (0.6)			6 (1)	4 (0.8)			7 (1)	6 (0.8)		
Died (%)	1464	152 (22)	154 (22.2)			134 (23.2)	108 (22.9)			157 (22.7)	159 (22)		
Home time (No of days)	1465	27.7 (39.5)	31.2 (47.6)	−3.02 (−8.18, 2.14)	0.25	28 (41.9)	26.3 (44)	1.55 (−4.31, 7.40)	0.60	28.6 (47.8)	30.9 (39.6)	−2.91 (−7.96, 2.14)	0.26
**Questionnaires**													
Barthel index	1383	50.3 (43.6)	48.2 (42.8)	1.24 (−3.15, 5.62)	0.58	48.4 (43.5)	48.4 (43)	1.29 (−3.70, 6.28)	0.61	48.1 (43.1)	49.5 (43.4)	−1.81 (−6.11, 2.49)	0.41
MoCA	722∗	11.8 (12.1)	11.1 (12.1)	0.30 (−1.30, 1.90)	0.71	12 (12.5)	11.1 (12.1)	−0.12 (−1.89, 1.65)	0.89	11 (12)	11.5 (12.1)	−0.43 (−1.98, 1.13)	0.59
MOCA, alive only	397∗	21.3 (6.6)	21.5 (5.9)	0.05 (−1.17, 1.28)	0.93	22.3 (6.1)	21.8 (5.7)	0.04 (−1.26, 1.34)	0.96	21 (6.5)	21.6 (6)	−0.26 (−1.48, 0.96)	0.68
EQ-5D-5L	1098	0.5 (0.4)	0.5 (0.4)	0.00 (−0.04, 0.05)	0.89	0.5 (0.4)	0.5 (0.4)	0.02 (−0.04, 0.07)	0.53	0.5 (0.4)	0.5 (0.4)	−0.03 (−0.07, 0.02)	0.24
EQ-VAS	1071	43.7 (34.5)	42.7 (34.5)	−0.14 (−4.19, 3.92)	0.95	41.1 (34.2)	41.7 (34.9)	−0.14 (−4.78, 4.50)	0.95	41.7 (34.4)	43.8 (34.7)	−2.26 (−6.23, 1.71)	0.27

Data are n (%), median [IQR], or mean (SD). Treatments effects are adjusted for stratification (country), minimisation (age, sex, stroke type, stroke severity, diabetes), and other baseline prognostic factors (e.g. pre-morbid mRS, atrial fibrillation, reperfusion treatment [alteplase and/or thrombectomy], time from onset to randomisation), and treatment allocation to the other two strata of the trial, unless otherwise stated. aDIM: adjusted difference in means. aHR: adjusted hazards ratio. aOR: adjusted odds ratio. Comparison by adjusted ordinal logistic regression (aOLR), multiple linear regression (aMLR), Cox proportional hazards regression (CPHR) or adjusted binary logistic regression (aBLR). mRS, modified Rankin Scale; MoCA, Montreal Cognitive Assessment; EQ-5D-5L, EuroQol 5D-5L; EQ-VAS, EuroQol-Visual Analogue Scale. Scores for death are included in questionnaire outcomes as follows: mRS, 6; Barthel Index, −5; EQ-5D-5L, 0; EQ-VAS, −1; MOCA, −1. ∗: During the COVID-19 pandemic, in-person visits were substituted by virtual visits for many patients, preventing assessment of the MoCA.

a Likelihood ratio test: for metoclopramide vs. no metoclopramide, *p* = 0.83; ceftriaxone vs. no ceftriaxone, *p* = 0.23; paracetamol vs. no paracetamol, *p* = 0.94.

**Table 3:** Early secondary outcomes and treatment restrictions.

	N	Metoclopramide				Ceftriaxone				Paracetamol			
		Yes	None	DIM/OR (95% CI)	*p*	Yes	None	DIM/OR (95% CI)	*p*	Yes	None	DIM/OR (95% CI)	*p*
**24 h**													
Body temperature °C	1258	36.8 (0.6)	36.7 (0.6)	0.02 (−0.05, 0.08)	0.61	36.8 (0.6)	36.8 (0.6)	−0.00 (−0.08, 0.07)	0.96	36.7 (0.5)	36.8 (0.6)	−0.12 (−0.19, −0.06)	<0.001
Temperature ≥ 38 °C (%)	1258	19 (3.2)	20 (3.3)	0.99 (0.44, 2.25)	0.98	18 (3.6)	15 (3.7)	0.95 (0.38, 2.35)	0.91	7 (1.2)	30 (4.8)	0.32 (0.13, 0.78)	0.012
**Day 7**													
mRS													
All patients	1482	4 [3,5]	4 [3,5]	0.88 (0.71, 1.10)	0.27	4 [3,5]	4 [3,5]	0.89 (0.69, 1.15)	0.38	4 [3,5]	4 [3,5]	1.05 (0.84, 1.30)	0.68
Ischaemic stroke	1259	4 [3,5]	4 [3,5]	0.88 (0.71, 1.10)	0.26	4 [3,5]	4 [3,5]	0.87 (0.67, 1.12)	0.28	4 [3,5]	4 [3,5]	1.06 (0.85, 1.31)	0.63
Intracerebral haemorrhage	203	4 [4,5]	5 [4,5]	0.86 (0.48, 1.53)	0.61	5 [4,5]	4 [4,5]	1.73 (0.80, 3.74)	0.16	5 [4,5]	5 [4,5]	0.77 (0.43, 1.37)	0.37
Death at 7 days (%)	1482	46 (6.6)	42 (6)	1.07 (0.62, 1.84)	0.81	34 (5.8)	29 (6)	0.77 (0.40, 1.46)	0.42	44 (6.3)	45 (6.1)	1.11 (0.65, 1.90)	0.69
**Infection (number of participants), clinical diagnosis**													
Any infection (%)	1482	199 (28.5)	175 (24.8)	1.20 (0.90, 1.60)	0.22	129 (21.9)	159 (33.1)	0.55 (0.39, 0.78)	<0.001	177 (25.2)	207 (28.3)	0.79 (0.60, 1.05)	0.11
Pneumonia (%)	1482	138 (19.8)	111 (15.7)	1.38 (0.98, 1.95)	0.063	102 (17.3)	89 (18.5)	0.77 (0.52, 1.15)	0.21	121 (17.2)	134 (18.3)	0.84 (0.60, 1.17)	0.31
Urinary tract infection (%)	1482	52 (7.4)	48 (6.8)	1.00 (0.60, 1.65)	1.00	12 (2)	59 (12.3)	0.21 (0.11, 0.44)	<0.001	41 (5.8)	64 (8.7)	0.53 (0.31, 0.88)	0.015
Other infections (%)	1482	24 (3.4)	26 (3.7)	0.89 (0.47, 1.68)	0.71	20 (3.4)	20 (4.2)	0.85 (0.40, 1.79)	0.66	25 (3.6)	25 (3.4)	1.32 (0.70, 2.49)	0.39
**Infection (number of participants), panel adjudicated**													
All any infection (%)	1487	71 (10.1)	61 (8.6)	1.07 (0.70, 1.64)	0.74	54 (9.2)	55 (11.4)	0.76 (0.48, 1.23)	0.27	60 (8.5)	80 (10.9)	0.72 (0.48, 1.09)	0.12
Pneumonia (%)	1487	46 (6.6)	35 (5)	1.27 (0.76, 2.14)	0.36	41 (6.9)	29 (6)	0.96 (0.54, 1.69)	0.88	40 (5.7)	48 (6.5)	0.79 (0.48, 1.32)	0.37
Urinary tract infection (%)	1487	19 (2.7)	21 (3)	0.67 (0.31, 1.44)	0.31	6 (1)	21 (4.4)	0.35 (0.13, 0.95)	0.039	16 (2.3)	24 (3.3)	0.63 (0.29, 1.35)	0.24
Other infections (%)	1487	7 (1)	7 (1)	1.24 (0.36, 4.27)	0.73	7 (1.2)	5 (1)	1.33 (0.30, 5.94)	0.71	5 (0.7)	10 (1.4)	0.68 (0.19, 2.40)	0.55

mRS, modified Rankin Scale. Data are n (%), mean (SD) or median [IQR]. Treatments effects are adjusted for stratification (country), minimisation (age, sex, stroke type, stroke severity, diabetes), and other baseline prognostic factors (e.g. pre-morbid mRS, atrial fibrillation, reperfusion treatment [alteplase and/or thrombectomy], time from onset to randomisation), and treatment allocation to the other two strata of the trial, unless otherwise stated. aOR: adjusted odds ratio, aDIM: adjusted difference in means. Comparison by adjusted ordinal logistic regression (aOLR), binary logistic regression (aBLR) or multiple linear regression (aMLR).

In the first seven days after stroke, physicians diagnosed an infection in 402 (27%) of 1487 patients: pneumonia in 269 patients (18.1%), urinary tract infection in 106 patients (7.1%), and other infections in 53 patients (3.6%). Twenty-six patients developed two types of infection. After review by an expert panel, rates were 5.9% for pneumonia, 2.8% for urinary tract infection, and 1.0% for any other infection. Prophylactic use of ceftriaxone prevented the occurrence of any infection (from 33.1% to 21.9%, based on the clinical diagnosis; aOR 0.55 [95% CI 0.39–0.78], *p* < 0.001), which was mainly driven by prevention of urinary tract infections (from 12.3% to 2%; aOR 0.21 [95% CI 0.11–0.44], *p* < 0.001) and a non-significant tendency to less pneumonia (17.3% vs. 18.5%; aOR 0.77 [95% CI 0.52–1.15], *p* = 0.21). In contrast, metoclopramide was associated with a non-significant increase in the rate of pneumonia (19.8% vs. 15.7%; aOR 1.38 [95% CI 0.98–1.95], *p* = 0.063; Table 3).

In a *post hoc* analysis, the median body temperature at 24 h was 36.7 °C (interquartile range, 36.4–37.1). At 24 h, the number of patients with a body temperature ≥37 °C, ≥37.5 °C, or ≥38.0 °C was 408 (32.4%), 159 (12.6%), and 40 (3.2%), respectively. Paracetamol reduced the mean body temperature at 24 h (36.7 °C [SD 0.5]) vs. 36.8 °C [SD 0.6]; difference in mean −0.12 [95% CI −0.19, −0.06], *p* < 0.001). Fever (≥38.0 °C) at 24 h was observed in 1.2% in patients randomised to paracetamol vs. 4.8% in controls (aOR 0.32 [95% CI 0.13–0.78], *p* = 0.012; Table 3).

**Fig. 3:**
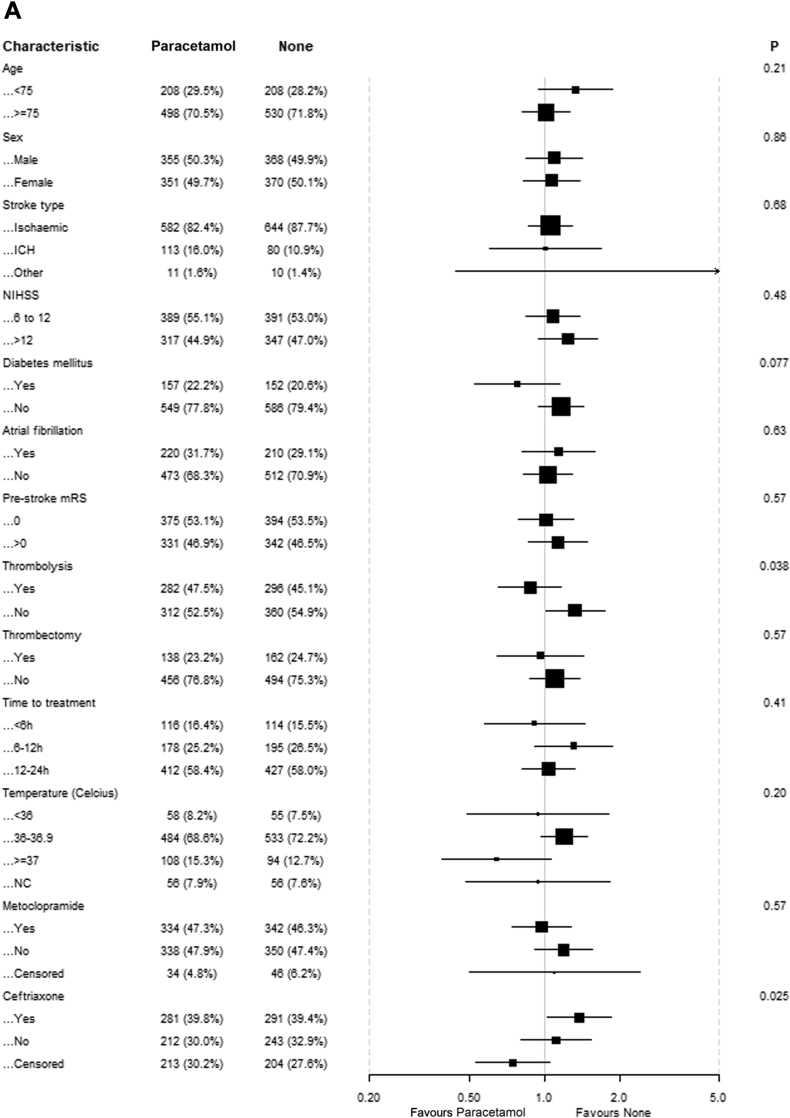

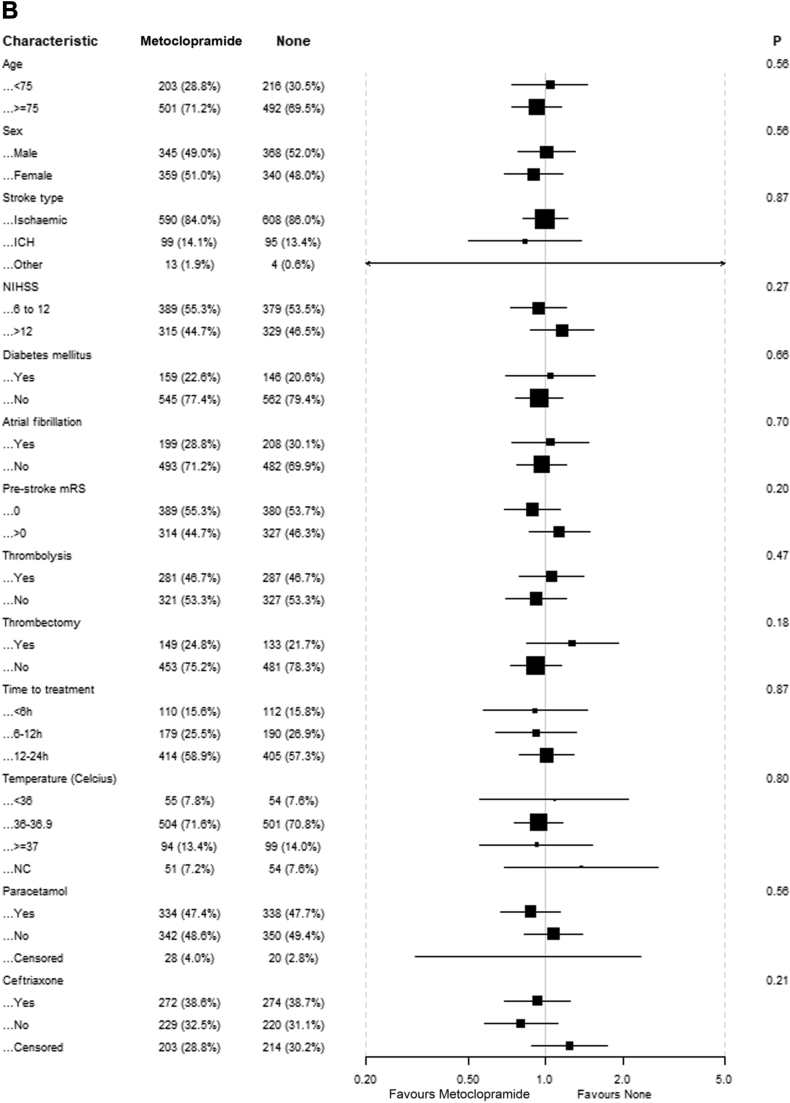

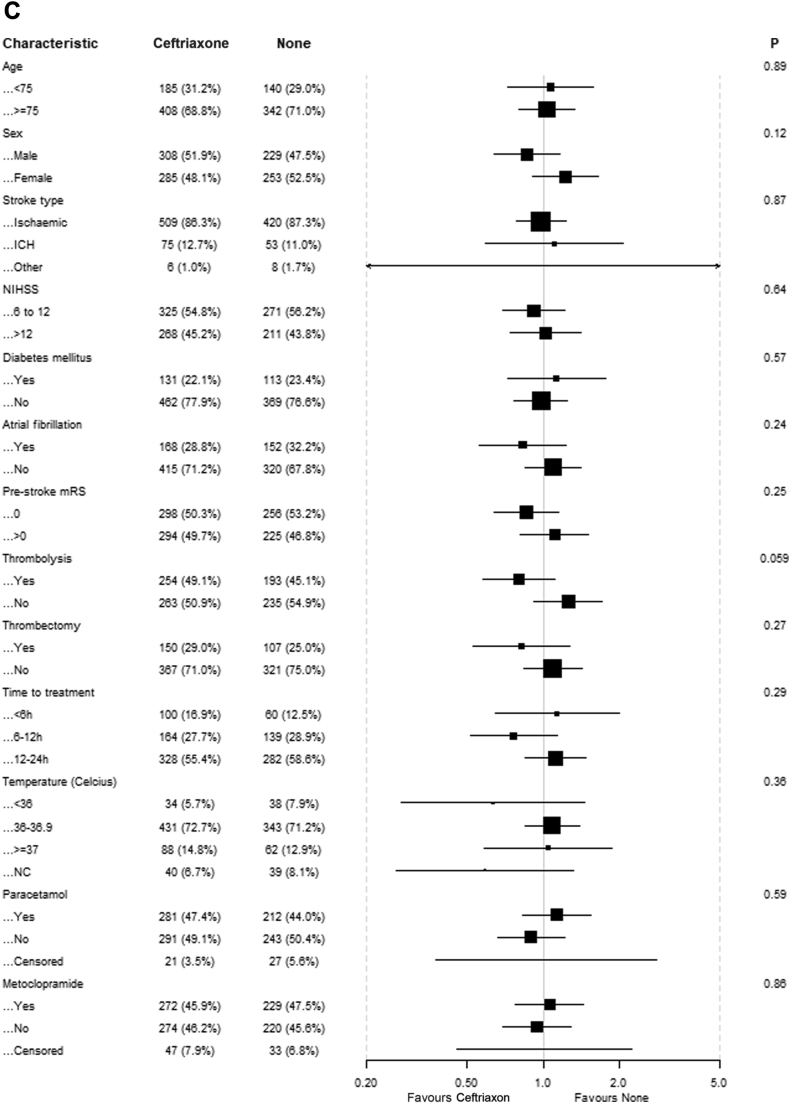
**Effect of treatment on mRS scores at 90 days in prespecified subgroups**. (A) Patients allocated to paracetamol vs patients allocated to no paracetamol; (B) Patients allocated to metoclopramide vs patients allocated to no metoclopramide; (C) Patients allocated to ceftriaxone vs patients allocated to no ceftriaxone.

There was no evidence of benefit of any of the study drugs in the predefined subgroups based on age, sex, stroke type, NIHSS, diabetes mellitus, atrial fibrillation, pre-stroke mRS, intravenous thrombolysis, endovascular thrombectomy and time to treatment (Fig. 3). For patients randomised to paracetamol, there were statistically significant interactions with intravenous thrombolysis and randomisation to ceftriaxone, suggesting that patients who received thrombolysis had better outcomes if treated with paracetamol and that patients randomised to ceftriaxone had worse outcomes if treated with paracetamol. There was no association between the time between stroke onset and start of treatment and functional outcome for any of the study drugs. There was also no association between baseline body temperature and the effect of paracetamol.

**Fig. 4:**
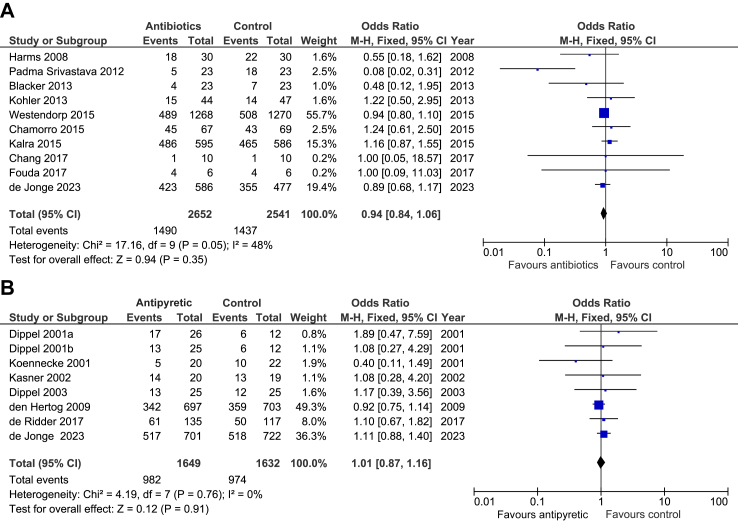
**Updated meta-analyses of studies assessing the effects of prophylactic antibiotics or antipyretic drugs on functional outcome after stroke.** Unfavourable outcome is defined as a modified Rankin Scale (mRS) score of 3 through 6. An odds ratio (OR) <1.0 indicates a positive effect of treatment. The ORs are unadjusted. (A) Ten out of 15 trials have been included in the meta-analysis, because no score on the mRS at three months was available for five trials. (B) Dippel 2001a and 2001b refer to two intervention groups in one study; the numbers in the control group have been divided by two to avoid multiple comparisons of the same patients. CI, confidence interval.

The numbers of SAEs and rates of death at seven days were similar between treatment groups. Two patients randomised to metoclopramide had an allergic reaction as SAE. One additional SAE was considered to be possibly related to the study drug by the safety desk (suicide attempt 19 days after start of treatment with metoclopramide). Six (1%) infections with *Clostridioides difficile* and 17 (2.9%) infections with ceftriaxone-resistant organisms were observed in patients randomised to ceftriaxone vs. 1 (0.2%; *p* = 0.45) and 9 (1.9%; *p* = 0.65) in the non-ceftriaxone group, respectively (Supplementary Table S4).

## Discussion

In this trial in patients aged 66 years or older, prophylactic use of metoclopramide, ceftriaxone, or paracetamol in the first four days after ischaemic stroke or intracerebral haemorrhage was safe but did not result in improvement of functional outcome or a reduction in the risk of death. Preventive antibiotics failed to reduce the rate of pneumonia in the first seven days, both as diagnosed by the local investigator and as diagnosed by an independent expert panel. Only a very modest reduction in body temperature at 24 h was seen in patients randomised to paracetamol.

Our finding that prophylactic ceftriaxone did not reduce the rate of pneumonia nor improve functional outcome is in line with those of previous trials of prophylactic antibiotic therapy after stroke.^10^^,^^19^, ^20^, ^21^ As compared with PASS, the only previous trial of ceftriaxone in patients with acute stroke, the rate of pneumonia in controls in PRECIOUS was higher (7% vs. 18.5%), most likely related to the higher median age (74 vs. 80 years) and greater stroke severity (median NIHSS score 5 vs. 11) in PRECIOUS.^19^ Nevertheless, we did not confirm a reduction in the rate of pneumonia with prophylactic use of ceftriaxone in patients at high risk of pneumonia suggested in a *post hoc* substudy of PASS.^11^ As in PASS, the use of prophylactic ceftriaxone in PRECIOUS was safe and not associated with an increased risk infections with *Clostridiodes difficile* or ceftriaxone-resistant micro-organisms. The rate of pneumonia in PRECIOUS was comparable with that in the cluster-randomised trial STROKE-INF, which assessed the effects of a range of different prophylactic antibiotics started within 48 h after stroke, and which also did not show a reduction in the rate of pneumonia with prophylactic treatment with antibiotics.^20^

Possible explanations for the lack of benefit of prophylactic antibiotics is that these have no added value over specialised care and meticulous clinical surveillance at a stroke unit, with early detection of fever and initiation of antibiotic treatment. The type of antibiotic could be insufficient to prevent pneumonia. However, ceftriaxone is a broad-spectrum third-generation cephalosporin and covers most bacteria believed to cause stroke-associated pneumonia.^22^ Because the risk of pneumonia is greatest in the first few days after stroke,^23^ the timing of treatment with ceftriaxone in PRECIOUS was appropriate. Alternatively, it has been suggested that stroke-associated pneumonia is not merely a bacterial infection, but chemical factors (e.g. aspiration of acid gastric content) play an important role and could lead to aspiration pneumonitis, less susceptible to antibiotic treatment.^19^ About 15% of all patients have radiological signs of aspiration or pulmonary infection already in the first few hours after stroke onset.^24^

In the small trial MAPS, prophylactic treatment with metoclopramide reduced the rate of pneumonia from 87% to 27% in patients with stroke fed via a nasogastric tube, presumably through its prokinetic and antiemetic properties.^8^ By contrast, prophylactic treatment with metoclopramide in the first four days after stroke did not reduce the rate of pneumonia in PRECIOUS and was even associated with a tendency towards an increased risk of pneumonia. The difference between the two trials could be explained by the duration of treatment, which was four days in PRECIOUS and 21 days in MAPS, considerably longer than the maximum duration of treatment recommended in the summary of product characteristics of metoclopramide. The effects of prophylactic therapy with metoclopramide during 21 days after stroke are currently tested in the MAPS 2 trial (ISRCTN14124645).

In our trial, prophylactic treatment with paracetamol decreased the mean body temperature at 24 h by 0.12 °C and reduced the incidence of fever (from 4.8% to 1.2%) at 24 h. This finding is in line with the largest previous trial on preventive treatment with paracetamol in patients with acute stroke (PAIS).^9^ The proportion of patients with a subfebrile temperature or fever in PRECIOUS was relatively low: just 12.6% of controls had a body temperature of ≥37.5 °C at 24 h, which is lower than the 22% after 24 h in previous studies.^25^ This may be related to the high age of patients in our trial which has been associated with a reduced tendency to develop hyperthermia.^26^ In addition, a quarter of patients in the control group received any antipyretic drug, most often paracetamol, in the first 24 h. In contrast to findings in PAIS, prophylactic treatment with paracetamol was associated with a tendency towards a worse functional outcome.

Our study was underpowered to assess whether a combination of drugs with different mechanisms to prevent stroke-associated pneumonia or fever is more effective than a single intervention. However, in the subsets of patients who received two or all three study drugs, functional outcome was similar to that in patients who were not allocated to these drugs. In the paracetamol stratum, we did find a statistically significant interaction with ceftriaxone, suggesting that paracetamol increased the risk of poor outcome in patients also treated with ceftriaxone. We are not aware of drug interactions between cephalosporins and paracetamol, and this finding is most likely due to the play of chance. We did not identify subgroups of patients in whom prophylactic use of metoclopramide, ceftriaxone, or paracetamol is likely to be of benefit.

Our study has several limitations. First, our study did not reach the target sample size of 3800 patients. Explanations for slow recruitment were regulatory hurdles causing delay in site initiation^27^ and the COVID-19 pandemic during which inclusion in clinical trials not related to COVID-19 was suspended in a large number of study sites and investigators had less time to include patients. After the main COVID-19 measures had been released, recruitment never caught up to pre-COVID numbers because of a loss of research routines, changed patient pathways, and a shift to more clinical activities for research personnel. Secondly, the use of open-label antiemetic, antibiotic, or antipyretic medication in patients allocated to the relevant control groups may have diluted any treatment effect. However, this does not invalidate our conclusion that prophylactic administration of metoclopramide, ceftriaxone, or paracetamol to every older patient with acute stroke and a moderate to severe neurological deficit is unlikely to be of benefit. Third, we did not collect information on the outcomes of a swallow test before randomisation and have no information on the number of patients who were actually dysphagic. Finally, the open-label design of our trial could have changed the treating physicians’ sensitivity to diagnose and report an infection or fever.

The neutral findings of our study are in line with those of other trials aiming to improve functional outcome after stroke through prevention or treatment of post-stroke complications or conditions, including hypertension,^28^ hyperglycaemia,^29^ aspiration,^30^ infections,^31^ fever,^31^^,^^32^ depression,^33^ immobility,^34^ or malnutrition.^35^ Only a combination of measures through organised inpatient (stroke unit) care has been shown to reduce the risk of death or dependency after stroke, although the quality of this evidence is considered just moderate.^6^ It therefore remains unknown which individual strategies drive the benefit of stroke unit care.

## Contributors

JCdJ, WMS, HR, PMB, BZ, DvdB, AHA, IA, AC, AF, LC, JD, JK, IK, MRM, GN, GR, GT, HBvdW designed the trial. HM, SP, SR, SC, PC, SFTMdB, RP, KK, BC were the principal investigators of the best recruiting sites. JCdJ and WS wrote the first draft of the paper. LJW and PMB performed/supervised the statistical analyses. All authors contributed to data collection and critically revised the draft of the paper. All authors had full access to all the data in the study and had final responsibility for the decision to submit for publication. HBvdW, JCdJ, WMS, LJW and PMB had full access to all the data and vouch for the completeness and accuracy of the data.

## Data sharing statement

De-identified individual participant data and a data dictionary defining each field in the set can be made available to others upon reasonable request to the corresponding author, subject to privacy regulation.

## Declaration of interests

LJW, BZ, IA, LC, IKJ, JeD, GN, HM, SR, SC, PC, SFTMdB, RP, KK, BC: none related. JCdJ, WMS, HR, JaD and MM report grants from the European Union, all paid to their institution. PMB reports having received grants from the UK National Institute of Health Research, and fees as consultant from CoMind, DiaMedica, Phagenesis and Roche. DvdB reports having received research grants from the European Union, The Netherlands for Health Research and Development, ItsMe Foundation, AMC Foundation and Roche; none related. AHA reports research grants from Boehringer Ingelheim, lectures fee from Abbvie, BMS/Pfizer, Novartis, Roche and Teva and participation in Advisory Board for Lundbeck, Abbvie and MSD; none related. AC reports grants from the European Union and Lombardy Region, for research paid to his institution, and fees as consultant or lecturer from Alexion Pharma, Daiichi Sanky, and Italfarmaco. JK reports lecturer fees from Boehringer Ingelheim, Pfizer and Servier, and travel grants from Boehringer Ingelheim and Servier, none related. SP received research support from BMS/Pfizer, Boehringer-Ingelheim, Daiichi Sankyo, European Union, German Federal Joint Committee Innovation Fund, and German Federal Ministry of Education and Research, Helena Laboratories and Werfen as well as speakers’ honoraria/consulting fees from Alexion, AstraZeneca, Bayer, Boehringer-Ingelheim, BMS/Pfizer, Daiichi Sankyo, Portola, and Werfen (all outside the submitted work). GT reports grants from the European Union, German Research Foundation, German Federal Ministry of Education and Research, German Innovation Fund for research paid to his institution, and fees as consultant or lecturer from Acandis, Alexion, Amarin, Bayer, Boehringer Ingelheim, Daiichi Sanky, BristolMyersSqibb/Pfizer, and Stryker. HBvdW reports having received grants from the European Union, the Dutch Heart Foundation, and Stryker for research, and funding for consultancy from Bayer and TargED, all paid to his institution.

## Appendix ASupplementary data

Supplementary Fig. S1 and Tables S1–S5

TLRHEUROPE-D-23-00354 statistical analysis plan.pdf

TLRHEUROPE-D-23-00354 study protocol.pdf

## References

[1] Westendorp W.F., Nederkoorn P.J., Vermeij J.D., Dijkgraaf M.G., van de Beek D. (2011). Post-stroke infection: a systematic review and meta-analysis. BMC Neurol.

[2] Greer D.M., Funk S.E., Reaven N.L., Ouzounelli M., Uman G.C. (2008). Impact of fever on outcome in patients with stroke and neurologic injury: a comprehensive meta-analysis. Stroke.

[3] Westendorp W.F., Vermeij J.D., Hilkens N.A. (2018). Development and internal validation of a prediction rule for post-stroke infection and post-stroke pneumonia in acute stroke patients. Eur Stroke J.

[4] de Jonge J.C., Wallet J., van der Worp H.B. (2019). Fever worsens outcomes in animal models of ischaemic stroke: a systematic review and meta-analysis. Eur Stroke J.

[5] Ringelstein E.B., Chamorro A., Kaste M. (2013). ESO Stroke Unit Certification Committee. European Stroke Organisation recommendations to establish a stroke unit and stroke center. Stroke.

[6] Langhorne P., Ramachandra S., Stroke Unit Trialists’ Collaboration (2020). Organised inpatient (stroke unit) care for stroke: network meta-analysis. Cochrane Database Syst Rev.

[7] Middleton S., McElduff P., Ward J. (2011). Implementation of evidence-based treatment protocols to manage fever, hyperglycaemia, and swallowing dysfunction in acute stroke (QASC): a cluster randomised controlled trial. Lancet.

[8] Warusevitane A., Karunatilake D., Sim J., Lally F., Roffe C. (2015). Safety and effect of metoclopramide to prevent pneumonia in patients with stroke fed via nasogastric tubes trial. Stroke.

[9] den Hertog H.M., van der Worp H.B., van Gemert H.M. (2009). The Paracetamol (Acetaminophen) in Stroke (PAIS) trial: a multicentre, randomised, placebo-controlled, phase III trial. Lancet Neurol.

[10] Westendorp W.F., Vermeij J.D., Smith C.J. (2021). Preventive antibiotic therapy in acute stroke patients: a systematic review and meta-analysis of individual patient data of randomized controlled trials. Eur Stroke J.

[11] Sluis W.M., Westendorp W.F., van de Beek D., Nederkoorn P.J., van der Worp H.B. (2022). Preventive ceftriaxone in patients at high risk of stroke-associated pneumonia. A post-hoc analysis of the PASS trial. PLoS One.

[12] Reinink H., de Jonge J.C., Bath P.M. (2018). PRECIOUS: prevention of complications to improve outcome in elderly patients with acute stroke. rationale and design of a randomised, open, phase III, clinical trial with blinded outcome assessment. Eur Stroke J.

[13] de Jonge J.C., Woodhouse L.J., Reinink H., van der Worp H.B., Bath P.M., PRECIOUS investigators (2020). PRECIOUS: prevention of complications to improve outcome in elderly patients with acute Stroke-statistical analysis plan of a randomised, open, phase III, clinical trial with blinded outcome assessment. Trials.

[14] Horan T.C., Andrus M., Dudeck M.A. (2008). CDC/NHSN surveillance definition of health care-associated infection and criteria for specific types of infections in the acute care setting. Am J Infect Control.

[15] Smith C.J., Kishore A.K., Vail A. (2015). Diagnosis of stroke-associated pneumonia: recommendations from the pneumonia in stroke consensus group. Stroke.

[16] Quinn T.J., Dawson J., Lees J.S., Chang T.P., Walters M.R., Lees K.R. (2008). Time spent at home poststroke: "home-time" a meaningful and robust outcome measure for stroke trials. Stroke.

[17] The Optimising Analysis of Stroke Trials (OAST) Collaboration (2007). Can we improve the statistical analysis of stroke trials? Statistical re-analysis of functional outcomes in stroke trials. Stroke.

[18] Bath P.M., Woodhouse L.J., Appleton J.P. (2018). Antiplatelet therapy with aspirin, clopidogrel, and dipyridamole versus clopidogrel alone or aspirin and dipyridamole in patients with acute cerebral ischaemia (TARDIS): a randomised, open-label, phase 3 superiority trial. Lancet.

[19] Westendorp W.F., Vermeij J.D., Zock E. (2015). The Preventive Antibiotics in Stroke Study (PASS): a pragmatic randomised open-label masked endpoint clinical trial. Lancet.

[20] Kalra L., Irshad S., Hodsoll J. (2015). Prophylactic antibiotics after acute stroke for reducing pneumonia in patients with dysphagia (STROKE-INF): a prospective, cluster-randomised, open-label, masked endpoint, controlled clinical trial. Lancet.

[21] Vermeij J.D., Westendorp W.F., Dippel D.W., van de Beek D., Nederkoorn P.J. (2018). Antibiotic therapy for preventing infections in people with acute stroke. Cochrane Database Syst Rev.

[22] Kishore A.K., Jeans A.R., Garau J. (2019). Antibiotic treatment for pneumonia complicating stroke: recommendations from the pneumonia in stroke consensus (PISCES) group. Eur Stroke J.

[23] de Jonge J.C., van de Beek D., Lyden P., Brady M.C., Bath P.M., van der Worp H.B. (2022). Temporal profile of pneumonia after stroke. Stroke.

[24] de Jonge J.C., Takx R.A.P., Kauw F., de Jong P.A., Dankbaar J.W., van der Worp H.B. (2020). Signs of pulmonary infection on admission chest computed tomography are associated with pneumonia or death in patients with acute stroke. Stroke.

[25] den Hertog H.M., van der Worp H.B., van Gemert H.M. (2011). An early rise in body temperature is related to unfavorable outcome after stroke: data from the PAIS study. J Neurol.

[26] Norman D.C., Yoshikawa T.T. (1996). Fever in the elderly. Infect Dis Clin North Am.

[27] de Jonge J.C., Reinink H., Colam B. (2021). Regulatory delays in a multinational clinical stroke trial. Eur Stroke J.

[28] Sandset E.C., Anderson C.S., Bath P.M. (2021). European Stroke Organisation (ESO) guidelines on blood pressure management in acute ischaemic stroke and intracerebral haemorrhage. Eur Stroke J.

[29] Zheng D., Zhao X. (2020). Intensive versus standard glucose control in patients with ischemic stroke: a meta-analysis of randomized controlled trials. World Neurosurg.

[30] Anderson C.S., Arima H., Lavados P. (2017). HeadPoST investigators and coordinators. Cluster-randomized, crossover trial of head positioning in acute stroke. N Engl J Med.

[31] Kuczynski A.M., Marzoughi S., Al Sultan A.S. (2020). Therapeutic hypothermia in acute ischemic stroke-a systematic review and meta-analysis. Curr Neurol Neurosci Rep.

[32] Fang J., Chen C., Cheng H., Wang R., Ma L. (2017). Effect of paracetamol (acetaminophen) on body temperature in acute stroke: a meta-analysis. Am J Emerg Med.

[33] Legg L.A., Rudberg A.S., Hua X. (2021). Selective serotonin reuptake inhibitors (SSRIs) for stroke recovery. Cochrane Database Syst Rev.

[34] AVERT Trial Collaboration group (2015). Efficacy and safety of very early mobilisation within 24 h of stroke onset (AVERT): a randomised controlled trial. Lancet.

[35] Dennis M., Lewis S., Cranswick G., Forbes J., FOOD Trial Collaboration (2006). FOOD: a multicentre randomised trial evaluating feeding policies in patients admitted to hospital with a recent stroke. Health Technol Assess.

