# Supplementary material for: Prevention of infections and fever to improve outcome in older patients with acute stroke (PRECIOUS): a randomised, open, phase III, multifactorial, clinical trial with blinded outcome assessment
Source: Lancet Reg Health Eur. 2023 Dec 1;36:100782. doi: 10.1016/j.lanepe.2023.100782 (PMC10698669; doi:10.1016/j.lanepe.2023.100782)
Supplement: Supplementary Fig. S1 and Tables S1–S5 [file mmc1.docx]

**Prevention of infections and fever to improve outcome in older patients with acute stroke (PRECIOUS): a randomised, open, phase III, multifactorial, clinical trial with blinded outcome assessment**

**Supplementary material**

**Table of content**

- Page 3: Supplement Table 1· Protocol violations in eligibility
- Page 4: Supplement Table 2· Compliance and cross-over in first 7 days
- Page 5: Supplement Table 3. Overview of safety
- Page 6: Supplement Table 4. Primary outcome, by dual treatment versus neither
- Page 7: Supplement Table 5. Primary outcome, by triple treatment versus none
- Page 8: Supplement Figure 1 a/b/c. Kaplan Meier of death for each intervention
- Page 11: Addendum to protocol
- Page 12: PRECIOUS investigators

**Supplement Table 1· Protocol violations in eligibility**

|  | | | **Metoclopramide** | | **Ceftriaxone** | | **Paracetamol** | |
| --- | --- | --- | --- | --- | --- | --- | --- | --- |
|  | **n** | **All** | **Yes** | **None** | **Yes** | **None** | **Yes** | **None** |
| Patients randomised |  | 1492 | 704 | 708 | 593 | 482 | 706 | 738 |
| Other diagnosis than stroke | 1492 | 21 (1·4) | 13 (1·8) | 4 (0·6) | 6 (1) | 8 (1·7) | 11 (1·6) | 10 (1·4) |
| NIHSS score ≤5 | 1492 | 0 | 0 | 0 | 0 | 0 | 0 | 0 |
| Age ≤65 years | 1492 | 0 | 0 | 0 | 0 | 0 | 0 | 0 |
| Start treatment >24 hours | 1492 | 80 (5·4) | 37 (5·3) | 42 (5·9) | 22 (3·7) | 20 (4·1) | 41 (5·8) | 38 (5·1) |
| Inclusion with active infection requiring antibiotic treatment | 1492 | 0 | 0 | 0 | 0 | 0 | 0 | 0 |
| Pre-stroke mRS 4 or 5 | 1492 | 3 (0·2) | 3 (0·4) | 0 (0) | 3 (0·5) | 0 (0) | 1 (0·1) | 2 (0·3) |
| Death is imminent | 1492 | 0 | 0 | 0 | 0 | 0 | 0 | 0 |
| Inclusion in treatment arm despite contra-indication | 1492 | 4 (0·3) | 3 (0·4) | 1 (0·1) | 2 (0·3) | 1 (0·2) | 2 (0·3) | 2 (0·3) |

Data are n (%). mRS, modified Rankin Scale.

**Supplement Table 2· Compliance and cross-over in first 7 days**

|  | | **Metoclopramide** | | | **Ceftriaxone** | | | **Paracetamol** | | |
| --- | --- | --- | --- | --- | --- | --- | --- | --- | --- | --- |
|  | **N** | **Yes** | **None** | **p** | **Yes** | **None** | **p** | **Yes** | **None** | **p** |
| N |  | 704 | 708 |  | 593 | 482 |  | 706 | 738 |  |
| Received all | 1492 | 251 (35·7) | - |  | 476 (80·3) | - |  | 277 (39·2) | - |  |
| Received 75-99% | 1492 | 206 (29·3) | - |  | 55 (9·3) | - |  | 273 (38·7) | - |  |
| Received 50-74% | 1492 | 119 (16·9) | - |  | 31 (5·2) | - |  | 72 (10·2) | - |  |
| Received 25-49% | 1492 | 84 (11·9) | - |  | 21 (3·5) | - |  | 42 (5·9) | - |  |
| Received <25% | 1492 | 44 (6·3) | - |  | 10 (1·7) | - |  | 42 (5·9) | - |  |
| Received any drug | 1492 | 700 (99·4) | - |  | 587 (99·0) | - |  | 702 (99·4) |  |  |
| Received any antiemetic | 1485 | 44 (6·3) | 68 (9·6) † | 0·021 | 46 (7·8) | 38 (7·9) | 0·93 | 59 (8·4) | 63 (8·6) | 0·88 |
| Received any antibiotic | 1486 | 195 (27·9) | 178 (25·2) | 0·25 | 121 (20·5) | 151 (31·5) † | <0·001 | 185 (26·3) | 198 (27) | 0·77 |
| Received any antipyretic | 1485 | 223 (31·9) | 237 (33·5) | 0·52 | 171 (29) | 169 (35·3) | 0·028 | 141 (20) | 326 (44·4) † | <0·001 |

† Open-label treatment administered to the relevant control group. Crossovers are recorded up to 7 days to allow for washout. Patients in the active treatment arms could receive additional anti-emetic, antibiotic, or antipyretic medication on top of metoclopramide, ceftriaxone, or paracetamol, respectively, if this was considered clinically indicated or after the treatment period of four days had been terminated

**Supplement Table 3. Overview of safety**

|  | | **Metoclopramide** | | | **Ceftriaxone** | | | **Paracetamol** | | |
| --- | --- | --- | --- | --- | --- | --- | --- | --- | --- | --- |
|  | **N** | **Yes** | **None** | **p** | **Yes** | **None** | **p** | **Yes** | **None** | **p** |
| All infections (%) | 1484 | 199 (28·4) | 175 (24·8) | 0·24 | 129 (21·9) | 159 (33·1) | <0·001 | 177 (25·1) | 207 (28·4) | 0·10 |
| Pneumonia (%) | 1483 | 138 (19·7) | 111 (15·8) | 0·067 | 102 (17·4) | 89 (18·5) | 0·21 | 121 (17·1) | 134 (18·4) | 0·29 |
| Urinary tract infection (%) | 1482 | 52 (7·4) | 48 (6·8) | 1·00 | 12 (2) | 59 (12·3) | <0·001 | 41 (5·8) | 64 (8·8) | 0·014 |
| *Clostridiodes difficile* infection (%) | 1482 | 3 (0·4) | 3 (0·4) | 0·73 | 6 (1) | 1 (0·2) | 0·45 | 1 (0·1) | 6 (0·8) | 0·37 |
| Other infections (%) | 1483 | 21 (3) | 23 (3·3) | 0·63 | 14 (2·4) | 19 (4) | 0·41 | 24 (3·4) | 19 (2·6) | 0·23 |
| Infection with ceftriaxone-resistant micro-organism (%)* | 1484 | 18 (2·6) | 9 (1·3) | 0·049 | 17 (2·9) | 9 (1·9) | 0·65 | 8 (1·1) | 19 (2·6) | 0·13 |
| SAEs | . |  |  |  |  |  |  |  |  |  |
| Number of participants with SAEs (%) | 1483 | 82 (11·7) | 85 (12·1) | 0·74 | 65 (11·1) | 54 (11·3) | 0·79 | 82 (11·6) | 87 (11·9) | 0·89 |
| By class |  |  |  |  |  |  |  |  |  |  |
| - Cardiovascular (%) | 1482 | 12 (1·7) | 11 (1·6) | 0·88 | 13 (2·2) | 7 (1·5) | 0·54 | 8 (1·1) | 15 (2·1) | 0·53 |
| - Nervous system (%) | 1483 | 52 (7·4) | 64 (9·1) | 0·97 | 47 (8) | 35 (7·3) | 0·63 | 58 (8·2) | 60 (8·2) | 0·72 |
| - Gastro-intestinal (%) | 1482 | 2 (0·3) | 1 (0·1) | 0·64 | 0 (0) | 2 (0·4) | 1·00 | 3 (0·4) | 0 (0) | <0·001 |
| - Respiratory (%) | 1482 | 2 (0·3) | 2 (0·3) | 0·43 | 3 (0·5) | 1 (0·2) | <0·001 | 2 (0·3) | 2 (0·3) | 0·57 |
| - Other (%) | 1482 | 17 (2·4) | 9 (1·3) | 0·094 | 7 (1·2) | 10 (2·1) | 0·85 | 13 (1·8) | 14 (1·9) | 0·79 |
| Related SAEs (%) | 1482 | 2 (0·3) | 0 (0) | - | 0 (0) | 0 (0) | - | 0 (0) | 2 (0·3) | - |
| SUSARS (%) | 1482 | 0 (0) | 0 (0) | - | 0 (0) | 0 (0) | - | 0 (0) | 0 (0) | - |

Outcomes are from the Infection and SAE databases with the exception of those marked with *. Data are number of participants with an event with (%). SAE, Severe Adverse Event; SAR, Severe Adverse Reaction; SUSAR, Severe Unexpected Serious Adverse Reaction. Comparisons made by binary logistic regression**.**

**Supplement Table 4. Primary outcome, by dual treatment versus neither**

|  | **Metoclopramide + Ceftriaxone** | | | | | **Paracetamol + Metoclopramide** | | | | | **Paracetamol + Ceftriaxone** | | | | |
| --- | --- | --- | --- | --- | --- | --- | --- | --- | --- | --- | --- | --- | --- | --- | --- |
|  | **N** | **Yes** | **None** | **DIM/OR (95% CI)** | **p** | **N** | **Yes** | **None** | **DIM/OR (95% CI)** | **p** | **N** | **Yes** | **None** | **DIM/OR (95% CI)** | **p** |
| **Day 90** |  |  |  |  |  |  |  |  |  |  |  |  |  |  |  |
| mRS, median [IQR] | 356 | 4 [2,5] | 4 [3,5] | 0·75 (0·48, 1·15) | 0·18 | 545 | 4 [2,5] | 4 [2,5] | 1·17 (0·81, 1·67) | 0·40 | 390 | 4 [3,6] | 4 [2,5] | 1·41 (0·91, 2·17) | 0·12 |
| Sensitivity analysis | . |  |  |  |  | . |  |  |  |  | . |  |  |  |  |
| mRS, unadjusted | 356 | 4 [2,5] | 4 [3,5] | 0·81 (0·56, 1·19) | 0·29 | 545 | 4 [2,5] | 4 [2,5] | 1·01 (0·74, 1·37) | 0·96 | 390 | 4 [3,6] | 4 [2,5] | 1·43 (1·00, 2·05) | 0·053 |
| mRS, imputed | 363 | 4 [2,5] | 4 [3,5] | 0·74 (0·48, 1·14) | 0·18 | 555 | 4 [2,5] | 4 [2,5] | 1·16 (0·81, 1·66) | 0·43 | 395 | 4 [3,6] | 4 [2,5] | 1·41 (0·91, 2·17) | 0·12 |
| mRS, mean | 356 | 3·5 (1·8) | 3·8 (1·7) | -0·26 (-0·61, 0·10) | 0·16 | 545 | 3·6 (1·8) | 3·5 (1·9) | 0·11 (-0·21, 0·42) | 0·51 | 390 | 4 (1·8) | 3·6 (1·8) | 0·26 (-0·11, 0·62) | 0·17 |
| mRS >2 (%) | 356 | 100 (71·4) | 167 (77·3) | 0·73 (0·39, 1·35) | 0·31 | 545 | 140 (69·3) | 239 (69·7) | 0·96 (0·58, 1·60) | 0·89 | 390 | 121 (80·1) | 173 (72·4) | 1·48 (0·78, 2·81) | 0·23 |
| Death (%) | 356 | 28 (20) | 47 (21·8) | 0·79 (0·45, 1·37) | 0·40 | 545 | 41 (20·3) | 74 (21·6) | 1·06 (0·67, 1·67) | 0·81 | 390 | 43 (28·5) | 56 (23·4) | 1·44 (0·90, 2·30) | 0·12 |
| **Day 7** |  |  |  |  |  |  |  |  |  |  |  |  |  |  |  |
| All infections (%) | 360 | 37 (26·2) | 76 (34·7) | 0·64 (0·36, 1·11) | 0·11 | 550 | 54 (26·7) | 89 (25·6) | 0·99 (0·61, 1·61) | 0·97 | 394 | 25 (16·4) | 83 (34·3) | 0·36 (0·19, 0·66) | 0·0011 |
| Pneumonia (%) | 360 | 29 (20·6) | 42 (19·2) | 0·97 (0·51, 1·84) | 0·92 | 550 | 30 (14·9) | 49 (14·1) | 1·03 (0·55, 1·93) | 0·94 | 394 | 23 (15·1) | 44 (18·2) | 0·70 (0·35, 1·40) | 0·31 |
| Urinary tract infection (%) | 360 | 5 (3·5) | 27 (12·3) | 0·27 (0·09, 0·81) | 0·019 | 550 | 22 (10·9) | 34 (9·8) | 0·76 (0·36, 1·60) | 0·46 | 394 | 1 (0·7) | 38 (15·7) | 0·05 (0·01, 0·38) | 0·0039 |
| Other infections (%) | 360 | 6 (4·3) | 11 (5) | 0·96 (0·28, 3·31) | 0·95 | 550 | 7 (3·5) | 12 (3·4) | 1·57 (0·53, 4·63) | 0·41 | 394 | 1 (0·7) | 6 (2·5) | 0·34 (0·03, 3·57) | 0·37 |

**Supplement Table 5. Primary outcome, by triple treatment versus none**

|  | | **Metoclopramide + Ceftriaxone + Paracetamol** | | | |
| --- | --- | --- | --- | --- | --- |
|  | **N** | **Yes** | **None** | **DIM/OR (95% CI)** | **p** |
| **Day 90** |  |  |  |  |  |
| mRS, median [IQR] | 245 | 4 [2,5] | 4 [3,5] | 0·94 (0·55, 1·61) | 0·82 |
| Sensitivity analysis | . |  |  |  |  |
| mRS, unadjusted | 245 | 4 [2,5] | 4 [3,5] | 0·85 (0·55, 1·32) | 0·47 |
| mRS, imputed | 248 | 4 [2,5] | 4 [3,5] | 0·95 (0·55, 1·62) | 0·84 |
| mRS, mean | 245 | 3·6 (1·9) | 3·8 (1·8) | -0·16 (-0·60, 0·28) | 0·47 |
| mRS >2 (%) | 245 | 91 (70·5) | 90 (77·6) | 0·62 (0·29, 1·33) | 0·22 |
| Death (%) | 245 | 32 (24·8) | 28 (24·1) | 0·99 (0·52, 1·87) | 0·98 |
| **Day 7** |  |  |  |  |  |
| All infections (%) | 247 | 32 (24·8) | 43 (36·4) | 0·38 (0·18, 0·80) | 0·011 |
| Pneumonia (%) | 247 | 26 (20·2) | 21 (17·8) | 0·74 (0·32, 1·72) | 0·48 |
| Urinary tract infection (%) | 247 | 3 (2·3) | 21 (17·8) | 0·11 (0·02, 0·52) | 0·0057 |
| Other infections (%) | 247 | 4 (3·1) | 3 (2·5) | 0·89 (0·12, 6·50) | 0·91 |

**Supplemental Figure 1 a/b/c. Kaplan Meier of death for each intervention**

1. **Paracetamol**

**
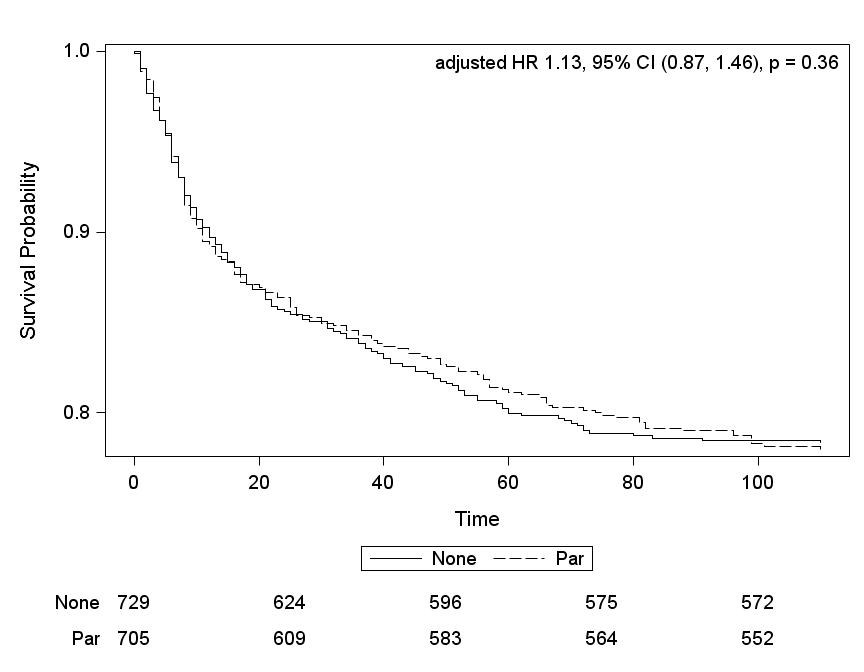
**

1. **Metoclopramide**

**
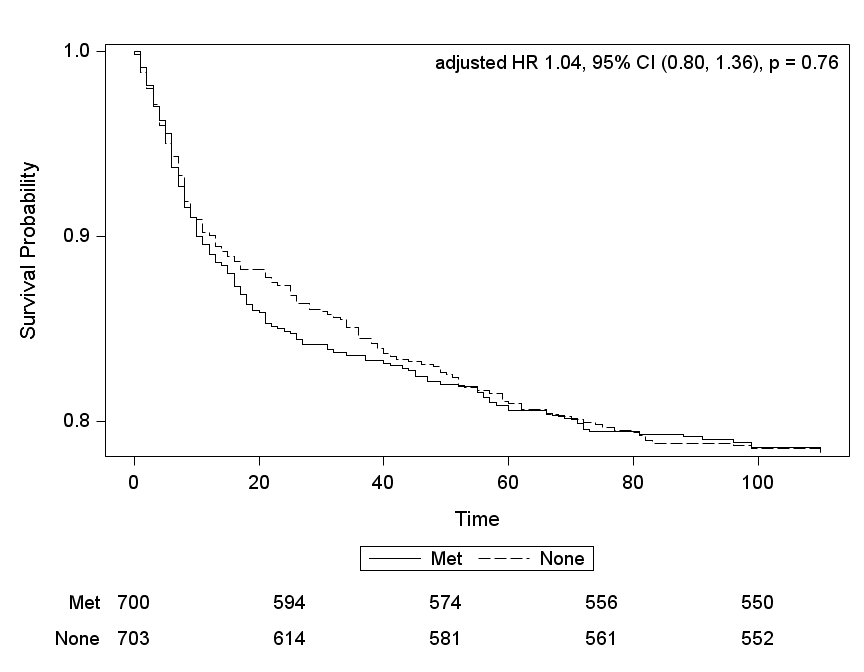
**

1. **Ceftriaxone**

**
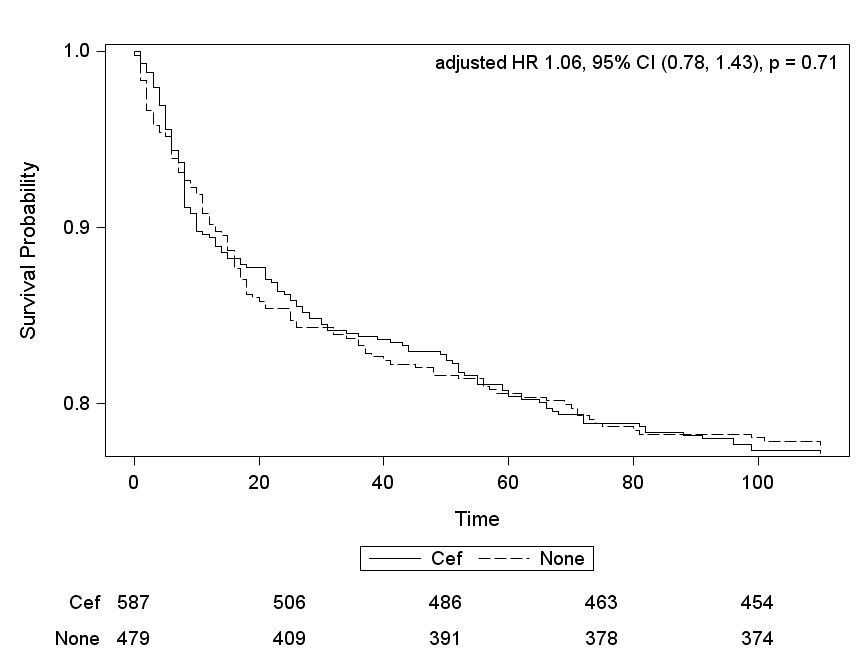
**

**Addendum**

Figure 1 of the study protocol does not illustrate the correct method of randomisation. Patients were randomised to metoclopramide or no metoclopramide, ceftriaxone or no ceftriaxone, and paracetamol or no paracetamol. All patients received standard of care. Therefore, for the correct interpretation of the treatment allocation process, we refer to the text of the protocol, instead of Figure 1.

PRECIOUS investigators

Bart van der Worp, Jeroen de Jonge, Wouter Sluis, Rik Reinink, Berber Zweedijk, University Medical Center Utrecht, Utrecht, the Netherlands. Diederik van de Beek, Willeke Westendorp, Amsterdam UMC, Amsterdam, the Netherlands; Henk Kerkhoff, Elles Zock, Corry Deuling, Albert Schweitzer Ziekenhuis, the Netherlands; Sebastiaan de Bruijn, Yvonne Drabbe-Coops, Amy Nijst, Haga ziekenhuis, the Hague, the Netherlands; Marieke Wermer, Ghislaine Holswilder, LUMC, Leiden, the Netherlands; Korne Jellema, Peggy Sorensen, Haaglanden Medical Center, the Hague, the Netherlands; Vincent Kwa, Sarah Godefrooij, OLVG, Amsterdam, the Netherlands; Ben Jansen, Esther Santegoets, St Elisabeth Ziekenhuis, Tilburg, the Netherlands; Tobien Schreuder, Tiny Sporken, Zuyderland Medical Center, Heerlen, the Netherlands; Sanne Zinkstok, Kitty Harrison, Tergooi Medical Center, Hilversum, the Netherlands; Walid Moudrous, Chantal van der Spoel, Arienne Verwijs-Bode, Maasstad ziekenhuis, Rotterdam, the Netherlands; Malcolm Macleod, Allan MacRaild, Royal Infirmary of Edinburgh, Edinburgh, UK; Ruth Davies, Jessica Teasdale, Arrowe Park Hospital, Wirral, UK; Anand Nair, Venetia Johnson, Calderdale Royal Hospital, Calderdale, UK; Dipankar Dutta, Matthew Robinson, Jill Greig, Gloucestershire Royal Hospital, Gloucester, UK; Rohan Pathansali, Deborah Ward, Jon Glass, Jonnie Aeron-Thomas, Myriam Aissa, Fong Kum Chan, Staci Conway, Beatrix Sari, Maria Tibaja, King’s College Hospital, London, UK; Martin Cooper, Inez Wynter, Sherwood Forest Hospital, Sutton in Ashfield, UK; Kailash Krishnan, Camille Hutchinson, Ben Jackson, Nottingham University Hospitals, Nottingham, UK; Khalid Rashed. Sarah Board, Yeovil District Hospital, Yeovil, UK; Louise Shaw, Suzanne Lucas, Joanne Avis, Telma Costa, Lauren Pearce, Royal United Hospital, Bath, UK; Alastair Wilson, Johann Selvarajah, Angela Welch, Shirley Mitchell, Queen Elisabeth University Hospital, Glasgow, UK; Jessica Redgrave, Emma Richards, Jo Howe, Royal Hallamshire Hospital, Sheffield, UK; Mary Joan Macleod, Janice Irvine, Vicky Taylor, Aberdeen Royal Infirmary, Aberdeen, UK; Philip Clatworthy, Kerry Smith, North Bristol NHS Trust, Bristol, UK; Vasileois Papavasileiou, Emelda Veraque, Dean Waugh, Leeds Teaching Hospitals NHS Trust, Leeds, UK; Vera Cvoro, Mandy Couser, Amanda McGrego, Victoria Hospital, Kirkcaldy, UK; Brian Clarke, Ghatala Rita, Cai Hua Sim, Sarah Stratton, St. George Hospital, London, UK; Omid Halse , Peter Wilding, Sheila Mashate, Vaishali Dave, Imperial College, London, UK; Usman Ghani, Faith Omoregie, Yates Kimberley, University Hospital Coventry and Warwickshire, Coventry, UK; Janika Korv, Tartu University Hospital, Tarty, Estonia; Katrin Antsov, Parnu Hospital, Parnu, Estonia; Katrin Gross-Paju, West Talinn Hospital, Tallinn, Estonia; Inga Kalju, Maarja Kaarlop, East Tallinn Hospital, Tallinn, Estonia; Gotz Thomalla, Hannes Appelbohm, Christoph Brosinski, University Medical Center Hamburg-Eppendorf, Hamburg, Germany; Gerhard Hamann, Michaela Vogel, Bezirkskrankenhaus Günzburg, Günzburg, Germany; Michael Rosenkranz, Stefan Boskamp, Albertinen-Krankenhaus Hamburg, Hamburg, Germany; Christoph Gumbinger, Peter Ringleb, Elisabeth Beyrle, Universitätsklinikum Heidelberg, Heidelberg, Germany; Georg Royl, Susanne Ribau, Universitätsklinikum Schleswig-Holstein, Lübeck, Germany; Sven Poli, Julia Zeller, Sonja Ruschitzka, Universitätsklinikum Tübingen, Tübingen, Germany; Susanne Müller, Andrea Schirmer, Universitätsklinikum Ulm, Ulm, Germany; George Ntaios, Efstathia Karagkiozi, Larissa University Hospital, Larissa, Greece; Sophie Vassilopoulou, Aeginition Hospital, Athens, Greece; Haralampos Milionis, Angelos Liontos, Ioannina University Hospital, Ioannina, Greece; Athanasios Protogerou, Stamatia Samara, Laiko Hospital, Athens, Greece; Efsthathios Manios, Efthalia Mitsikosta, Alexandra Hospital, Athens, Greece; Laszlo Csiba, Krisztina Buzás-Petrócz, Csilla Vér, University of Debrecen, Debrecen, Hungary; Dániel Bereczki, Andrea Kovacs, Semmelweis University, Budapest, Hungary; Gábor Jakab, Uzsoki Hospital, Budapest, Hungary; Ferenc Nagy, Lõrinczy Ritta, Puskásné Emmer Mária, Jávorszky Ödön Hospital, Vác, Hungary; András Folyovich, Nadim Al-Muhanna, Szent János Hospital, Budapest, Hungary; László Szapáry, Eszter Jozifek, University of Pecs, Pecs, Hungary; Alfonso Ciccone, Giorgio Silvestrelli, Paola Danesi, Marco Russo, ASST di Mantova, Mantova, Italy; Nicola Gilberti, Spedali Civili, Brescia, Italy; Enrico Righetti, Ospedale Castiglione del Lago, Castiglione del Lago, Italy; Stefano Ricci, Maria Elena Mattace, Silvia Cenciarelli, Ospedale di Città di Castello, Città di Castello, Italy; Stefano Ricci, Ospedale Gubbio – Gualdo Tadino, Branca, Italy; Pietro Bassi, Ospedale San Giuseppe, Milano, Italy; Simona Marcheselli, IRCCS Istituto Clinico Humanitas, Rozzano, Italy; Alessia Giossi, ASST Cremona, Cremona, Italy; Paolo Candelaresi, Giovanna Servillo, Ospedale Antonio Cardarelli, Napoli, Italy; Eivind Berge, Anne Hege Aamodt, Oslo University Hospital, Oslo, Norway; Anne Gro Holtan, Notodden Sykehus, Notodden, Norway; Sameer Maini, Alesund Hospital, Alesund, Norway; Iwona Kurkowska, Michal Karlinski, Institute of Psychiatry and Neurology, Warsaw, Poland; Waldemar Fryze, Malgorzata Krzyzanowska, University of Gdansk, Gdansk, Poland; Waldemar Brola, Hospital Sw. Lukasza, Konskie, Poland; Piotr Sobolewski, Szpital Specjalistyczny Ducha Swietego, Sandomierz, Poland; Marta Bilik, Samodzielny Publiczny Specjalistyczny Szpital Zachodni im. św. Jana Pawła II, Grodzisk Mazowiecki, Poland.
