# Supplementary material for: Prevention of infections and fever to improve outcome in older patients with acute stroke (PRECIOUS): a randomised, open, phase III, multifactorial, clinical trial with blinded outcome assessment
Source: Lancet Reg Health Eur. 2023 Dec 1;36:100782. doi: 10.1016/j.lanepe.2023.100782 (PMC10698669; doi:10.1016/j.lanepe.2023.100782)
Supplement: TLRHEUROPE-D-23-00354 statistical analysis plan.pdf [file mmc2.pdf]

PRECIOUS: PREvention of Complications to Improve OUtcome in elderly patients with acute Stroke. Statistical analysis plan of a randomised, open, phase III, clinical trial with blinded outcome assessment

Jeroen C de Jonge,[1]† Lisa J Woodhouse,[2]† Hendrik Reinink,[1]† H Bart van der Worp,[1]‡ Philip M Bath,[2,3]‡ for the PRECIOUS investigators

[1] Department of Neurology and Neurosurgery, Brain Center, University Medical Center Utrecht, Utrecht University, Utrecht, The Netherlands.

[2] Stroke Trials Unit, Division of Clinical Neuroscience, University of Nottingham, Nottingham, United Kingdom

[3] Stroke, Nottingham University Hospitals NHS Trust, Nottingham, United Kingdom

† ‡ These authors contributed equally to this paper.

Chief investigator / corresponding author:

H. Bart van der Worp, MD, PhD
Department of Neurology and Neurosurgery
Brain Center, University Medical Center Utrecht, Utrecht University
Heidelberglaan 1003584 CX Utrecht, The Netherlands
E: H.B.vanderWorp@umcutrecht.nl
T: +31 88 7571833 / F: +31 30 2542100

Statistician: L.J. Woodhouse

Trial registration number: ISRCTN82217627

Key words: stroke, complications, elderly, ceftriaxone, metoclopramide, paracetamol

ABSTRACT

Rationale. Aspiration, infections, and fever are common in the first days after stroke, especially in older patients. The occurrence of these complications has been associated with an increased risk of death or dependency.

Aims and design. PREvention of Complications to Improve OUtcome in elderly patients with acute Stroke **(**PRECIOUS) is an international, multi-centre, 3x2 factorial, randomised, controlled, open-label clinical trial with blinded outcome assessment, which will assess whether prevention of aspiration, infections, or fever with metoclopramide, ceftriaxone, paracetamol, respectively, or any combination of these, in the first four days after stroke onset improves functional outcome at 90 days in elderly patients with acute stroke.

Discussion. This statistical analysis plan provides a technical description of the statistical methodology and unpopulated tables and figures. The paper is written prior to data lock and unblinding of treatment allocation.

PRECIOUS is registered: ISRCTN82217627 **(**date of registration 22-9-2015; the trial was prospectively registered).

Background

In the first days after stroke, about half of all patients develop one or more complications, including aspiration, infections, or fever. The risk of developing these events is greater in patients of higher age or with more severe stroke (1–3). These complications can impede functional recovery, prolong hospital admissions, and are independently associated with an increased risk of death or long-term dependency (1,2,4–11). The risk of developing these complications can be reduced by very simple, safe and inexpensive measures, such as metoclopramide for the management of dysphagia, antibiotics for the prevention of infections, and paracetamol for the prevention of fever, but it is uncertain whether these measures also improve functional outcome (12–15). In some, generally small, randomised trials, preventive treatment with these drugs not only convincingly reduced the risks of aspiration, infections, or fever by one third to one half, but was also associated with clear trends towards a lower risk of death or poor outcome (12–15). However, in two large randomised clinical trials, preventive treatment with antibiotics did not improve functional outcomes (16,17). Guidelines of the European Stroke Organisation concluded that there is insufficient evidence from randomised trials to make strong recommendations on whether, when and to whom preventive antibiotic or antipyretic treatment should be given after ischaemic stroke or intracerebral haemorrhage (18,19). The PREvention of Complications to Improve OUtcome in elderly patients with acute Stroke (PRECIOUS) trial will assess whether prevention of aspiration, infections, or fever with metoclopramide, ceftriaxone, paracetamol, or any combination of these in the first four days after stroke onset improves functional outcome at 90 days in older patients with acute stroke. The current paper describes the statistical analysis plan (SAP) of the trial and conforms to the guidelines set by Gamble et al (20). The details of the study protocol of the PRECIOUS trial have been published earlier

(21). PRECIOUS has received funding from the *European Union's Horizon 2020 research and innovation programme* under grant agreement No 634809.

Study methods

PRECIOUS is an international, multi-centre, multi-factorial, randomised, controlled, phase-III, open-label clinical trial with blinded outcome assessment (PROBE). The primary objective is to assess whether prevention of aspiration, infections, or fever with metoclopramide, ceftriaxone, paracetamol, or any combination of these in the first four days after stroke onset improves functional outcome at 90 days in older patients with acute stroke. Patients will be randomly allocated in a 2*2*2 factorial design to any combination of open-label oral, rectal, or intravenous metoclopramide (10 mg thrice daily); intravenous ceftriaxone (2000 mg once daily); oral, rectal, or intravenous paracetamol (1000 mg four times daily), or usual care, started within 24 hours after symptom onset and continued for 4 days or until complete recovery or discharge from hospital, if earlier. In patients with moderate to severe renal impairment or with severe hepatic impairment, the dose of metoclopramide is reduced to 5 mg thrice daily, and in patients with end-stage renal disease to 2.5 mg thrice daily. Patients will be stratified according to country (Estonia, Germany, Greece, Hungary, Italy, the Netherlands, Norway, Poland, United Kingdom) and there will be 5 minimisation factors: age (66 – 75 years; >75 years), sex (male vs. female), stroke type (ischaemic stroke vs. intracerebral haemorrhage), stroke severity (NIHSS 6-12 vs. >12) and diabetes Mellitus (yes vs. no). 3800 patients will be recruited, based on the sample size calculation described in the previously published protocol (21).

Statistical interim analyses and stopping guidance

An independent Data and Safety Monitoring Board (DSMB) will conduct unblinded interim analyses after 600, 1200, 1800, 2400, and 3000 patients have completed follow-up to assess the safety of the interventions in the trial. With respect to efficacy, the DSMB will conduct unblinded interim analyses after 2400 patients had their final follow-up. DSMB members will receive listings of all SAE reports as well as unblinded aggregate summaries of data by treatment groups for review in closed meetings. The results of these interim analyses are confidential and limited to the members of DSMB.

Timing of final analysis

This statistical analysis plan (SAP) will be signed off by the trial Steering Committee and then submitted for publication prior to data lock and final analysis. The final statistical analysis will be performed once recruitment has ceased, final follow-up and final outcome adjudication have been completed, final data have been checked and any errors corrected, and the database has been locked. The analyses will be carried out according to the current statistical analysis plan. The statistical analyses will be performed by the Nottingham Stroke Trial Unit (NSTU) at the University of Nottingham (UNOTT) in collaboration with the UMC Utrecht.

Trial population

The study population will consist of patients aged 66 years or older who are hospitalised with moderately severe to severe (National Institutes of Health Stroke Scale (NIHSS) ≥6) acute ischaemic stroke or intracerebral haemorrhage. Patients will only be included if treatment can be started within 24 hours of stroke onset. For a complete overview of the inclusion and exclusion criteria, we refer to the study protocol (21). Patients are planned to be recruited in

about 80 hospitals in 9 European countries over a period of about four years. To increase the generalisability of the findings, these countries are distributed across Europe, and include Estonia, Germany, Greece, Hungary, Italy, the Netherlands, Norway, Poland, and the United Kingdom. For the same reason, the trial will recruit patients both in academic and regional hospitals.

3. Statistical Analysis

Primary outcome

The primary outcome measure is the score on the modified Rankin Scale (mRS) at 90 days (± 14 days). The mRS is an ordinal scale ranging from 0-6 (22). The mRS assessment at 90 days will be during a hospital/home visit or by telephone, and the assessment or a report thereof will be recorded using a digital video camera. Three blinded raters will view the videotape and adjudicate a score on the mRS.

Primary outcome analysis

For each patient, a median mRS score will be calculated from the three mRS scores obtained through centralised adjudications by raters who are blinded to treatment allocation. The use of three scores increases the precision in scoring and statistical power as compared to a single mRS assessment (23). The primary effect estimate will be the difference in the mRS scores between the active treatment group and controls assessed using ordinal logistic regression, and will be expressed as an odds ratio with 95% confidence interval (24). The primary analysis will be performed on all randomised patients with a valid mRS score at 90 days. The distribution of the mRS scores will be shown as a figure. Three separate primary analyses will be performed for each intervention versus their respective controls (e.g. metoclopramide vs. non-metoclopramide). The primary analyses will be adjusted for stratification (country),

minimisation (age, sex, stroke type, stroke severity, diabetes), and other baseline prognostic (e.g. pre-morbid mRS, atrial fibrillation, reperfusion treatment [alteplase and/or thrombectomy], time from onset to randomisation) factors, and treatment allocation for the other two strata of the trial.

Primary outcome subgroup analysis

Comparison of the effect of the three intervention groups vs. their respective controls on the primary outcome will be performed in the following pre-specified subgroups (assuming sufficient numbers in each subgroup) with assessment of interaction between treatment and the minimisation factors (these subgroup analyses are considered hypothesis-generating):

- Age (≤75, > 75 years);
- Sex (male, female);
- Stroke type (ischaemic stroke, intracerebral haemorrhage);
- Stroke severity (NIHSS 6 – 12, >12);
- Diabetes mellitus (yes, no);

In addition, the interaction between treatment and other baseline factors will be assessed:

- Presence of atrial fibrillation (yes, no);
- Pre-stroke mRS score (0, >0);
- Reperfusion treatment (alteplase and/or mechanical thrombectomy);
- Time to treatment (<6, ≥6 hours <12 hours, ≥12 hours);
- Treatment allocation for the other two trial strata (paracetamol – active, control; ceftriaxone – active, control; metoclopramide – active, control). Since the study is not powered to detect interactions between the three interventions, these interactions will be investigated in secondary analyses.

Sensitivity analyses

Four sensitivity analyses of the mRS will also be performed: unadjusted ordinal logistic regression; adjusted analysis of mRS following regression imputation of missing data; multiple linear regression on the mean mRS score for each participant, and binary logistic regression on mRS>2.

Secondary outcomes

The following secondary outcomes will be assessed at 7 days (± 1 day) or at discharge, if earlier:

- Infections in the first 7 days (± 1 day; frequency, type, and *Clostridium difficile* infections). Infections will be categorised as diagnosed by the clinician, and as judged by an independent adjudication committee (masked to treatment allocation);
- 3rd generation cephalosporin resistance in the first 7 days (± 1 day), detected as part of routine clinical practice;
- Antimicrobial use during the first 7 days, converted to units of defined daily doses according to the classification of the WHO Anatomical Therapeutic Chemical Classification System with Defined Daily Doses Index
- Serious adverse events (SAEs) in the first 7 days.
- In a subgroup of patients: presence of Extended-Spectrum Beta-Lactamase (ESBL)-producing bacteria as detected by PCR in a rectal swab at day 7 (± 1 day, or at discharge, if earlier).

The following secondary outcomes will be assessed at 90 days (± 14 days):

- Death;

- Unfavourable functional outcome, defined as mRS 3 to 6;
- Disability assessed with the score on the Barthel Index (BI);
- Cognition assessed with the Montreal Cognitive Assessment (MoCA);
- Quality of life assessed with the EuroQol 5D-5L (EQ-5D-5L), and EQ-visual analogue scale (EQ-VAS)
- Home time: the number of nights among the first 90 since stroke onset that are spent in the patient's own home or a relative's home. Resource use will be censored at 90 days. Where final follow-up occurs earlier, the last known placement will be extrapolated to 90 days;
- Patient location over first 90 days (± 14 days): hospital; rehabilitation service; chronic nursing facility; home.

Analysis of secondary outcomes

Binary logistic regression will be used for binary outcomes (e.g. mRS >2). Cox proportional hazards regression for time to events (e.g. death). Ordinal logistic regression will be used for ordered categorical data (e.g. mRS). Multiple linear regression will be used for continuous outcomes (e.g. BI, EQ-VAS). Patients with missing outcome data will be excluded from the analysis.

Missing data and death

Patients without a primary outcome assessment at 90 ± 14 days will be considered as a lost to follow-up. The total amount of patients who are lost to follow-up will be recorded and calculated for each treatment arm. The primary analysis will be performed on all randomised patients with a valid mRS score at 90 days. In a sensitivity analysis, missing mRS data will be imputed using multiple regression-based imputation.

For the secondary outcome measures (Barthel Index, MoCA, EQ-5D-5L, EQ-VAS), patients who die will be assigned a value one unit worse than any living value. This way, patients who die cannot be given a score similar to the worst score of patients who are alive, and it ensures that all patients will be included in the analysis. Potential scores, with worst with dead added, are:

- Modified Rankin Scale (mRS), 0 to 5 with death = 6
- Barthel Index (BI), 100 to 0 with death = -5
- EuroQol 5D-5L (EQ-5D-5L), -0.5 to 1 with death = 0
- EuroQol visual analogue scale (EQ-VAS), 0 to 100 with death = -1
- Montreal cognitive assessment (MoCA), 0 to 30 with death = -1.

Safety outcomes

In the first 7 days after randomisation, all SAEs will be reported and described by duration (start and stop dates), severity, outcome, treatment, and relation to the investigational medical product (IMP), or if unrelated, the cause. All SAEs will be tabulated per treatment stratum. In addition, any SAE occurring between day 7 and the end of follow-up on day 90 (± 14 days) for which a causal relationship between the IMP and the SAE is considered at least a reasonable possibility (i.e., SARs and SUSARs) should be reported as other SAEs.

Treatment restrictions

The presence of any treatment restriction will be recorded at baseline and during the hospital phase, and classified as 1. Do not resuscitate; 2. Do not intubate and ventilate; 3. Withhold other treatments that may prolong life; 4. Withhold food; 5. Withhold fluids; and 6. Palliation (e.g. with morphine or a benzodiazepine). Any combination of these strategies is possible.

The primary study will report on the frequency of each treatment restriction, further analyses on this topic will be published in future sub-group analyses.

Minimising bias

PRECIOUS is an open-label clinical trial and both patients and treating physicians are therefore aware of the assigned treatment. Knowledge of treatment allocation can influence outcome assessment, and unblinded trials like PRECIOUS are therefore at risk of detection bias. In addition, despite its apparent simplicity, assessment of the score on the mRS has been associated with considerable inter-observer variability, especially in multicentre studies, and may therefore affect trial power and treatment effect size. In PRECIOUS, these two major issues are minimised through 1) online training and certification of outcome assessors via a link on the PRECIOUS website; and 2) central outcome assessment by three blinded adjudicators based on digital video recordings of the 90-day outcome interviews. This central adjudication by trained adjudicators offers several benefits (23):

1. Blinding is assured;
2. Standardisation is possible across multiple regions and cultures;
3. Statistical power is enhanced through the use of three repeated assessments;
4. The estimate of treatment effect size is restored (since statistical noise leads to underestimation)
5. It provides independent validation of the information that is collected, thereby minimising the risk of fraud;
6. Site staff perform to a higher standard when aware that there will be review or audit of their activity.

In addition, the risk of bias is reduced by performing the statistical analyses according to the intention-to-treat principle and adjusting for the minimisation factors, other relevant baseline characteristics, and treatment allocation for the other two strata of the trial.

Statistical principles

Confidence intervals and P values

Analyses will be two-sided $p<0.05$ with 95% confidence intervals presented. The trial is testing the effect of the interventions on mRS and analyses in subgroups and on other outcomes are considered hypothesis-generating. Hence, no adjustment will be made for multiplicity of testing.

Alpha spending

The Data Monitoring Committee performs safety assessments using the Haybittle-Peto boundary rule ($p<0.001$); hence, no significant spending of alpha will occur during the trial. All analyses will be two-tailed and p-values of <0.05 will denote statistical significance; 95% confidence intervals will be provided. Adjustment for multiple comparisons will not be performed but all contrasts will be declared.

Compliance

Compliance with allocated treatment will be tabulated. For each of the three study drugs, the number of received dosages will be calculated (maximum of four for ceftriaxone, twelve for metoclopramide and sixteen for paracetamol). The number of patients who received the first dosage within the time window of 24 hours will also be presented; if the dosage was not given within 24 hours, the reason will be given (withdrawn informed consent, death, human error, other reason).

Analysis populations

All efficacy analyses will be performed on the intention-to-treat population. The robustness of the primary and key secondary analyses will be assessed in the per-protocol population. Safety analyses will be performed on the safety population.

The following population definitions will be used:

- Intention-to-treat in primary efficacy analysis: All randomised participants who received any study medication and with a valid mRS score recorded at 90 days.
- Intention-to-treat in primary safety analysis: All randomised participants with a vital status recorded at 90 days.
- Per-protocol: All participants in the intention-to-treat population who are deemed to have no major protocol violations that could interfere with the objectives of the study.

Patients with protocol violations in trial eligibility will be included in the intention to treat population, but excluded in the per-protocol analysis. Patients who withdrew informed consent before initiating treatment will be excluded from analysis. If (per accident) multiple randomisations are performed for a single patient, the result of the first randomisation will be used.

Current status

The trial received approval from the central Medical Ethics Committee of the University Medical Center Utrecht, The Netherlands on 3 February 2016. The Dutch National Competent Authority (Centrale Commissie Mensgebonden Onderzoek (CCMO)), declared to have no objection against the execution of the clinical trial within the Netherlands on 17

November 2015. In addition, the national (and local, if applicable) medical ethical committees and competent authorities of the other 8 participating countries have approved the trial. The first patient was included in May 2016. The analysis and reporting of the trial will be in accordance with CONSORT guidelines. After publication of the trial, to promote the independent re-use of PRECIOUS data, a coded dataset will be made available in a public data repository within 18 months of the final follow-up of the last patient. Coded data will also be included in the Virtual International Stroke Trials Archive (VISTA).

Table 1. Baseline characteristics

	All	Paracetamol	Control	Metoclopramide	Control	Ceftriaxone	Control
Total patients randomised							
Age (years)	Mean (SD)	Mean (SD)	Mean (SD)	Mean (SD)	Mean (SD)	Mean (SD)	Mean (SD)
Sex, male (%)	n (%)	n (%)	n (%)	n (%)	n (%)	n (%)	n (%)
Premorbid mRS [/6]	Median [IQR]	Median [IQR]	Median [IQR]	Median [IQR]	Median [IQR]	Median [IQR]	Median [IQR]
Ethnicity, white (%)	n (%)	n (%)	n (%)	n (%)	n (%)	n (%)	n (%)
**Medical History (%)**							
- Atrial fibrillation	n (%)	n (%)	n (%)	n (%)	n (%)	n (%)	n (%)
- Hypercholesterolaemia	n (%)	n (%)	n (%)	n (%)	n (%)	n (%)	n (%)
- Hypertension	n (%)	n (%)	n (%)	n (%)	n (%)	n (%)	n (%)
- Diabetes mellitus	n (%)	n (%)	n (%)	n (%)	n (%)	n (%)	n (%)
- Obstructive pulmonary disease	n (%)	n (%)	n (%)	n (%)	n (%)	n (%)	n (%)
- Previous stroke	n (%)	n (%)	n (%)	n (%)	n (%)	n (%)	n (%)
- Immunocompromised	n (%)	n (%)	n (%)	n (%)	n (%)	n (%)	n (%)
**Smoking, current**							
- Never	n (%)	n (%)	n (%)	n (%)	n (%)	n (%)	n (%)
- Ever	n (%)	n (%)	n (%)	n (%)	n (%)	n (%)	n (%)
- Currently	n (%)	n (%)	n (%)	n (%)	n (%)	n (%)	n (%)
**Pre-stroke method of food intake**							
- Normal food	n (%)	n (%)	n (%)	n (%)	n (%)	n (%)	n (%)
- Oral softened food or fluids only	n (%)	n (%)	n (%)	n (%)	n (%)	n (%)	n (%)
- Nasogastric tube	n (%)	n (%)	n (%)	n (%)	n (%)	n (%)	n (%)
- Percutaneous endoscopic gastrostomy	n (%)	n (%)	n (%)	n (%)	n (%)	n (%)	n (%)
- Intravenous only	n (%)	n (%)	n (%)	n (%)	n (%)	n (%)	n (%)

**Use of drugs 3 days before randomisation**							
- Paracetamol	n (%)	n (%)	n (%)	n (%)	n (%)	n (%)	n (%)
- Metoclopramide	n (%)	n (%)	n (%)	n (%)	n (%)	n (%)	n (%)
- Ceftriaxone	n (%)	n (%)	n (%)	n (%)	n (%)	n (%)	n (%)
Time, onset to randomisation (min)	Mean (SD)	Mean (SD)	Mean (SD)	Mean (SD)	Mean (SD)	Mean (SD)	Mean (SD)
**Stroke type (%)**							
Ischaemic stroke	n (%)	n (%)	n (%)	n (%)	n (%)	n (%)	n (%)
Intracerebral haemorrhage	n (%)	n (%)	n (%)	n (%)	n (%)	n (%)	n (%)
Other diagnosis	n (%)	n (%)	n (%)	n (%)	n (%)	n (%)	n (%)
NIHSS (/42)	Mean (SD)	Mean (SD)	Mean (SD)	Mean (SD)	Mean (SD)	Mean (SD)	Mean (SD)
Systolic BP (mmHg)	Mean (SD)	Mean (SD)	Mean (SD)	Mean (SD)	Mean (SD)	Mean (SD)	Mean (SD)
Diastolic BP (mmHg)	Mean (SD)	Mean (SD)	Mean (SD)	Mean (SD)	Mean (SD)	Mean (SD)	Mean (SD)
Heart rate (bpm)	Mean (SD)	Mean (SD)	Mean (SD)	Mean (SD)	Mean (SD)	Mean (SD)	Mean (SD)
Body temperature (ºC)	Mean (SD)	Mean (SD)	Mean (SD)	Mean (SD)	Mean (SD)	Mean (SD)	Mean (SD)
**Acute stroke treatment (%)**							
- Intravenous thrombolysis	n (%)	n (%)	n (%)	n (%)	n (%)	n (%)	n (%)
- Mechanical thrombectomy	n (%)	n (%)	n (%)	n (%)	n (%)	n (%)	n (%)

Data are n (%) or median [IQR]. mRS, modified Rankin Scale; NIHSS, National Institutes of Health Stroke scale; BP, blood pressure.

Table 2. Primary outcome. Analyses are adjusted except where stated

	Analysis	Paracetamol	Control	DIM or OR (95% CI	Metoclopramide	Control	DIM or OR (95% CI	Ceftriaxone	Control	DIM or OR (95% CI
mRS, median	aOLR	Median [IQR]	Median [IQR]	aOR (95% CI)	Median [IQR]	Median [IQR]	aOR (95% CI)	Median [IQR]	Median [IQR]	aOR (95% CI)
**Sensitivity analyses**										
mRS, unadjusted	OLR	Median [IQR]	Median [IQR]	OR (95% CI)	Median [IQR]	Median [IQR]	OR (95% CI)	Median [IQR]	Median [IQR]	OR (95% CI)
mRS, imputed	aOLR	Median [IQR]	Median [IQR]	aOR (95% CI)	Median [IQR]	Median [IQR]	aOR (95% CI)	Median [IQR]	Median [IQR]	aOR (95% CI)
mRS, mean	aMLR	Mean (SD)	Mean (SD)	aDIM (95% CI)	Mean (SD)	Mean (SD)	aDIM (95% CI)	Mean (SD)	Mean (SD)	aDIM (95% CI)
mRS >2	aBLR	n (%)	n (%)	aOR (95% CI)	n (%)	n (%)	aOR (95% CI)	n (%)	n (%)	aOR (95% CI)
Death	aCPHR	n (%)	n (%)	aHR (95% CI)	n (%)	n (%)	aHR (95% CI)	n (%)	n (%)	aHR (95% CI)

Data are n (%), median [IQR], mean (SD). aDIM: adjusted difference in means. aHR: adjusted hazards ratio. aOR: adjusted odds ratio. Comparison by adjusted ordinal logistic regression (aOLR), multiple linear regression (aMLR), Cox proportional hazards regression (CPHR) or adjusted binary logistic regression (aBLR)

Table 3. Secondary outcome assessment at 90 days

	Analysis	Paracetamol	Control	OR (95% CI)	Metoclopramide	Control	OR (95% CI)	Ceftriaxone	Control	OR (95% CI)
**mRS, median**										
Ischaemic stroke	aOLR	Median [IQR]	Median [IQR]	aOR (95% CI)	Median [IQR]	Median [IQR]	aOR (95% CI)	Median [IQR]	Median [IQR]	aOR (95% CI)
Intracerebral haemorrhage	aOLR	Median [IQR]	Median [IQR]	aOR (95% CI)	Median [IQR]	Median [IQR]	aOR (95% CI)	Median [IQR]	Median [IQR]	aOR (95% CI)
Other diagnosis	aOLR	Median [IQR]	Median [IQR]	aOR (95% CI)	Median [IQR]	Median [IQR]	aOR (95% CI)	Median [IQR]	Median [IQR]	aOR (95% CI)
Mortality	aCPHR	n (%)	n (%)	aHR (95% CI)	n (%)	n (%)	aHR (95% CI)	n (%)	n (%)	aHR (95% CI)
**Patient location**	aOLR			aOR (95% CI)			aOR (95% CI)			aOR (95% CI)
Hospital		n (%)	n (%)		n (%)	n (%)		n (%)	n (%)	
Rehabilitation service		n (%)	n (%)		n (%)	n (%)		n (%)	n (%)	
Nursing home		n (%)	n (%)		n (%)	n (%)		n (%)	n (%)	
Home		n (%)	n (%)		n (%)	n (%)		n (%)	n (%)	
Home time (No of days)	aMLR	Mean (SD)	Mean (SD)	aDIM (95% CI)	Mean (SD)	Mean (SD)	aDIM (95% CI)	Mean (SD)	Mean (SD)	aDIM (95% CI)
**Questionnaires**										
Barthel Index	aMLR	Mean (SD)	Mean (SD)	aDIM (95% CI)	Mean (SD)	Mean (SD)	aDIM (95% CI)	Mean (SD)	Mean (SD)	aDIM (95% CI)

MoCA	aMLR	Mean (SD)	Mean (SD)	aDIM (95% CI)	Mean (SD)	Mean (SD)	aDIM (95% CI)	Mean (SD)	Mean (SD)	aDIM (95% CI)
EQ-5D-5L	aMLR	Mean (SD)	Mean (SD)	aDIM (95% CI)	Mean (SD)	Mean (SD)	aDIM (95% CI)	Mean (SD)	Mean (SD)	aDIM (95% CI)
EQ-VAS	aMLR	Mean (SD)	Mean (SD)	aDIM (95% CI)	Mean (SD)	Mean (SD)	aDIM (95% CI)	Mean (SD)	Mean (SD)	aDIM (95% CI)

Data are n (%) or median [IQR]. aDIM: adjusted difference in means. aOR: adjusted odds ratio. aHR adjusted hazards ratio. Comparison by adjusted ordinal logistic regression (aOLR), Cox Proportional Hazards regression (aCPHR) or multiple linear regression (aMLR). mRS, modified Rankin Scale; MoCA, Montreal Cognitive Assessment; EQ-5D-5L, EuroQol 5D-5L; EQ-VAS, EuroQol-Visual Analogue Scale

Figure 1. Trial profile

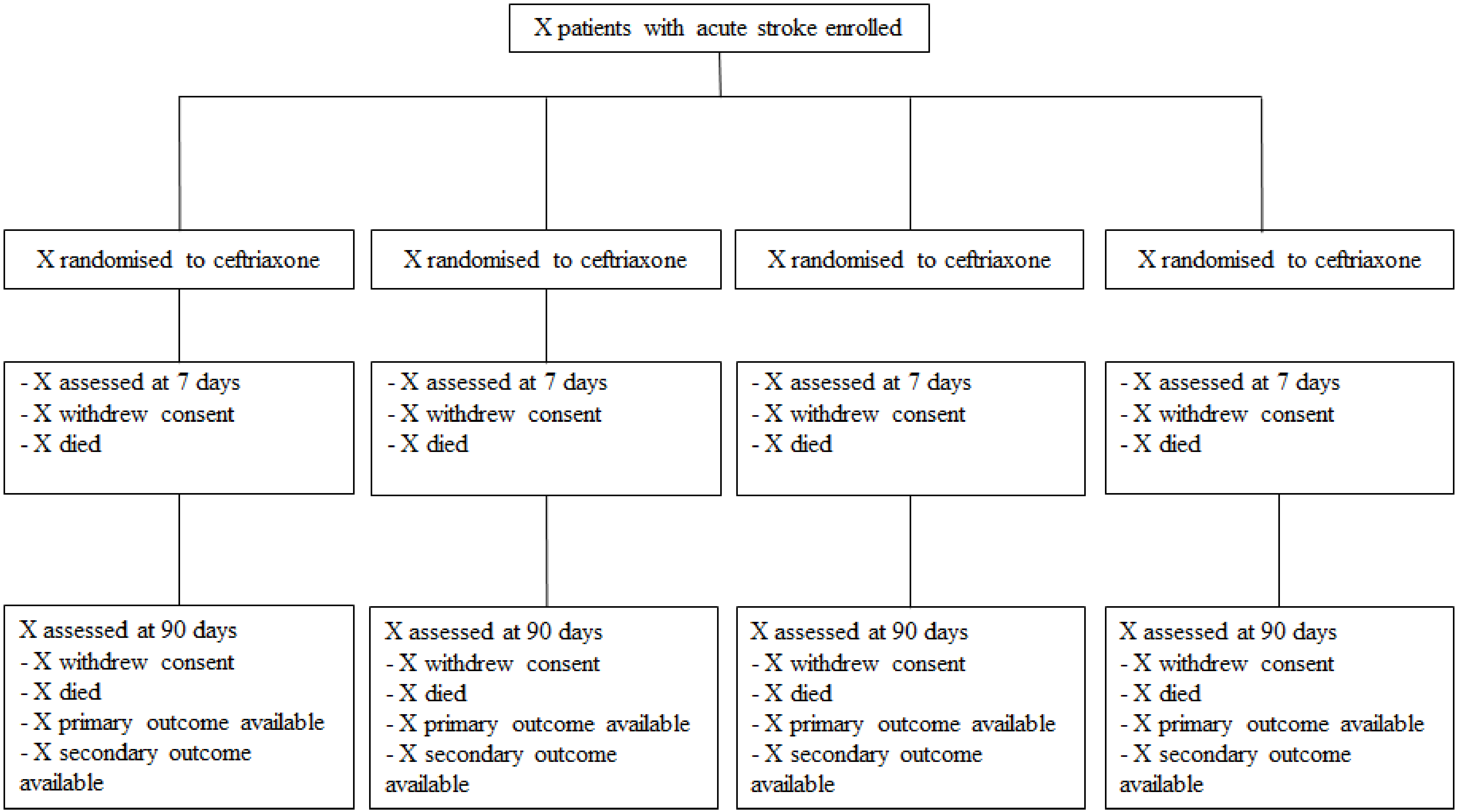

Figure 2 a/b/c. Distribution of modified Rankin Scale for each intervention using median mRS value for each participant

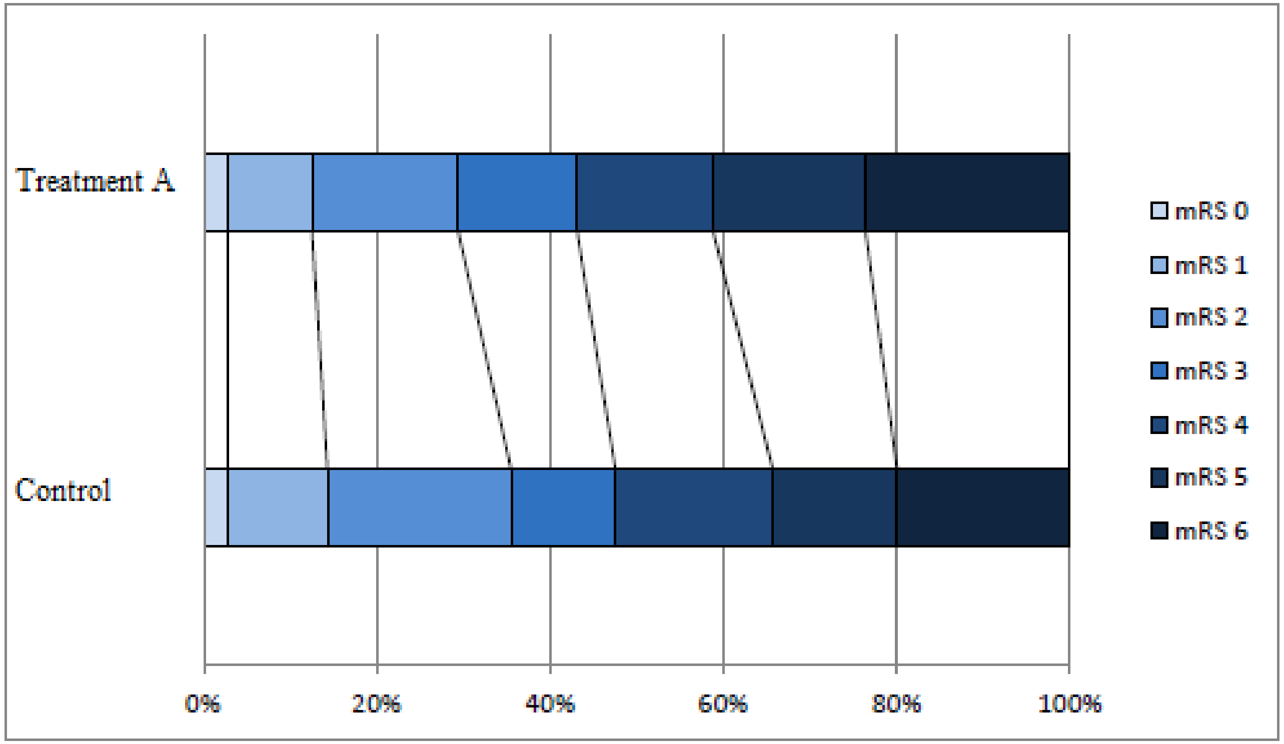

.

Example of a distribution of the modified Rankin Score at 3 months. The figure is an example, with dummy treatments and scores.

Figure 3. Subgroup analysis - shown as forest plot. Adjusted analysis with interaction term

	Paracetamol	Control	aOR (95% CI)	Inter-action P	Metoclopramide	Control	aOR (95% CI)	Inter-action P	Ceftriaxone	Control	aOR (95% CI)	Inter-action P
**Age**				+				+				+
Age <75 years	n/N (%)	n/N (%)	aOR (95% CI)		n/N (%)	n/N (%)	aOR (95% CI)		n/N (%)	n/N (%)	aOR (95% CI)	
Age >75 years	n/N (%)	n/N (%)	aOR (95% CI)		n/N (%)	n/N (%)	aOR (95% CI)		n/N (%)	n/N (%)	aOR (95% CI)	
**Sex**				+				+				+
Male	n/N (%)	n/N (%)	aOR (95% CI)		n/N (%)	n/N (%)	aOR (95% CI)		n/N (%)	n/N (%)	aOR (95% CI)	
Female	n/N (%)	n/N (%)	aOR (95% CI)		n/N (%)	n/N (%)	aOR (95% CI)		n/N (%)	n/N (%)	aOR (95% CI)	
**Stroke type**				+				+				+
Ischemic stroke	n/N (%)	n/N (%)	aOR (95% CI)		n/N (%)	n/N (%)	aOR (95% CI)		n/N (%)	n/N (%)	aOR (95% CI)	
Intracerebral haemorrhage	n/N (%)	n/N (%)	aOR (95% CI)		n/N (%)	n/N (%)	aOR (95% CI)		n/N (%)	n/N (%)	aOR (95% CI)	
Other diagnosis	n/N (%)	n/N (%)	aOR (95% CI)		n/N (%)	n/N (%)	aOR (95% CI)		n/N (%)	n/N (%)	aOR (95% CI)	
**Stroke severity**				+				+				+
NIHSS 6-12	n/N (%)	n/N (%)	aOR (95% CI)		n/N (%)	n/N (%)	aOR (95% CI)		n/N (%)	n/N (%)	aOR (95% CI)	

NIHSS >12	n/N (%)	n/N (%)	aOR (95% CI)		n/N (%)	n/N (%)	aOR (95% CI)		n/N (%)	n/N (%)	aOR (95% CI)	
**Diabetes Mellitus**				+				+				+
Yes	n/N (%)	n/N (%)	aOR (95% CI)		n/N (%)	n/N (%)	aOR (95% CI)		n/N (%)	n/N (%)	aOR (95% CI)	
No	n/N (%)	n/N (%)	aOR (95% CI)		n/N (%)	n/N (%)	aOR (95% CI)		n/N (%)	n/N (%)	aOR (95% CI)	
**Atrial Fibrillation**				+				+				+
Yes	n/N (%)	n/N (%)	aOR (95% CI)		n/N (%)	n/N (%)	aOR (95% CI)		n/N (%)	n/N (%)	aOR (95% CI)	
No	n/N (%)	n/N (%)	aOR (95% CI)		n/N (%)	n/N (%)	aOR (95% CI)		n/N (%)	n/N (%)	aOR (95% CI)	
**Pre-stroke mRS**				+				+				+
0	n/N (%)	n/N (%)	aOR (95% CI)		n/N (%)	n/N (%)	aOR (95% CI)		n/N (%)	n/N (%)	aOR (95% CI)	
>0	n/N (%)	n/N (%)	aOR (95% CI)		n/N (%)	n/N (%)	aOR (95% CI)		n/N (%)	n/N (%)	aOR (95% CI)	
**Treatment with alteplase**				+				+				+
Yes	n/N (%)	n/N (%)	aOR (95% CI)		n/N (%)	n/N (%)	aOR (95% CI)		n/N (%)	n/N (%)	aOR (95% CI)	
No	n/N (%)	n/N (%)	aOR (95% CI)		n/N (%)	n/N (%)	aOR (95% CI)		n/N (%)	n/N (%)	aOR (95% CI)	

**Thrombectomy**				+				+				+
Yes	n/N (%)	n/N (%)	aOR (95% CI)		n/N (%)	n/N (%)	aOR (95% CI)		n/N (%)	n/N (%)	aOR (95% CI)	
No	n/N (%)	n/N (%)	aOR (95% CI)		n/N (%)	n/N (%)	aOR (95% CI)		n/N (%)	n/N (%)	aOR (95% CI)	
**Time to treatment**				+				+				+
< 6 hours	n/N (%)	n/N (%)	aOR (95% CI)		n/N (%)	n/N (%)	aOR (95% CI)		n/N (%)	n/N (%)	aOR (95% CI)	
6-12 hours	n/N (%)	n/N (%)	aOR (95% CI)		n/N (%)	n/N (%)	aOR (95% CI)		n/N (%)	n/N (%)	aOR (95% CI)	
12-24 hours	n/N (%)	n/N (%)	aOR (95% CI)		n/N (%)	n/N (%)	aOR (95% CI)		n/N (%)	n/N (%)	aOR (95% CI)	
**Treatment allocation to other treatment strata**				+				+				+
Paracetamol	-	-	-	-	n/N (%)	n/N (%)	aOR (95% CI)		n/N (%)	n/N (%)	aOR (95% CI)	
Metoclopramide	n/N (%)	n/N (%)	aOR (95% CI)		-	-	-	-	n/N (%)	n/N (%)	aOR (95% CI)	
Ceftriaxone	n/N (%)	n/N (%)	aOR (95% CI)		n/N (%)	n/N (%)	aOR (95% CI)		-	-	-	-

Data are n/N (%). aOR: adjusted odds ratio. Comparison by adjusted ordinal logistic regression with adjustment for an interaction term. This table will be presented as Forest plots in the final publication.

Supplement Table 1. Protocol violations in eligibility

	Paracetamol	Control	Metoclopramide	Control	Ceftriaxone	Control
	N	N	N	N	N	N
Other diagnosis than stroke	n (%)	n (%)	n (%)	n (%)	n (%)	n (%)
NIHSS score of ≤5	n (%)	n (%)	n (%)	n (%)	n (%)	n (%)
Age ≤65 years	n (%)	n (%)	n (%)	n (%)	n (%)	n (%)
Start treatment >24 hours	n (%)	n (%)	n (%)	n (%)	n (%)	n (%)
Inclusion with active infection requiring antibiotic treatment	n (%)	n (%)	n (%)	n (%)	n (%)	n (%)
Pre-stroke mRS ≥4	n (%)	n (%)	n (%)	n (%)	n (%)	n (%)
Death is imminent	n (%)	n (%)	n (%)	n (%)	n (%)	n (%)
Inclusion in treatment arm despite contra-indication	n (%)	n (%)	n (%)	n (%)	n (%)	n (%)

Data are n (%). mRS, modified Rankin Scale.

Supplement Table 2. Compliance and cross-over in first 7 days

	Paracetamol	Control	P	Metoclopramide	Control	P	Ceftriaxone	Control	P
	N	N		N	N		N	N	
Received all allocated dosages	n (%)	-	-	n (%)	-	-	n (%)	-	-
Received 75-99% of dosages	n (%)	-	-	n (%)	-	-	n (%)	-	-
Received 50-<75% of dosages	n (%)	-	-	n (%)	-	-	n (%)	-	-
Received 25-<50% of dosages	n (%)	-	-	n (%)	-	-	n (%)	-	-
Received 0-<25% of dosages	n (%)	-	-	n (%)	-	-	n (%)	-	-
Received any antibiotic drug	n (%)	n (%)		n (%)	n (%)		n (%)	n (%)	
Received any antipyretic drug	n (%)	n (%)		n (%)	n (%)		n (%)	n (%)	
Received any antipyretic drug for four days at least once	n (%)	n (%)		n (%)	n (%)		n (%)	n (%)	
Received any anti-emetic drug	n (%)	n (%)		n (%)	n (%)		n (%)	n (%)	

Received any anti-emetic drug for four days at least once	n (%)	n (%)	n (%)	n (%)	n (%)	n (%)

Data are n (%). Comparisons made by binary logistic regression**.**

Supplement Table 3. Secondary outcomes and treatment restrictions at 7 days

	Analysis	Paracetamol	Control	OR (95% CI)	Metoclopramide	Control	OR (95% CI)	Ceftriaxone	Control	OR (95% CI)
**mRS, median**										
All patients	aOLR	Median [IQR]	Median [IQR]	aOR (95% CI)	Median [IQR]	Median [IQR]	aOR (95% CI)	Median [IQR]	Median [IQR]	aOR (95% CI)
Ischemic stroke	aOLR	Median [IQR]	Median [IQR]	aOR (95% CI)	Median [IQR]	Median [IQR]	aOR (95% CI)	Median [IQR]	Median [IQR]	aOR (95% CI)
Haemorrhagic stroke	aOLR	Median [IQR]	Median [IQR]	aOR (95% CI)	Median [IQR]	Median [IQR]	aOR (95% CI)	Median [IQR]	Median [IQR]	aOR (95% CI)
Mortality at 7 days	aBLR	n (%)	n (%)	aOR (95% CI)	n (%)	n (%)	aOR (95% CI)	n (%)	n (%)	aOR (95% CI)
Any treatment restriction	-	n (%)	n (%)	-	n (%)	n (%)	-	n (%)	n (%)	-
**Infection**										
All infections	aBLR	n (%)	n (%)	aOR (95% CI)	n (%)	n (%)	aOR (95% CI)	n (%)	n (%)	aOR (95% CI)
Pneumonia	aBLR	n (%)	n (%)	aOR (95% CI)	n (%)	n (%)	aOR (95% CI)	n (%)	n (%)	aOR (95% CI)

Urinary tract infection	aBLR	n (%)	n (%)	aOR (95% CI)	n (%)	n (%)	aOR (95% CI)	n (%)	n (%)	aOR (95% CI)
Other infections	aBLR	n (%)	n (%)	aOR (95% CI)	n (%)	n (%)	aOR (95% CI)	n (%)	n (%)	aOR (95% CI)
**Infections based on expert panel**										
All infections	aBLR	n (%)	n (%)	aOR (95% CI)	n (%)	n (%)	aOR (95% CI)	n (%)	n (%)	aOR (95% CI)
Pneumonia	aBLR	n (%)	n (%)	aOR (95% CI)	n (%)	n (%)	aOR (95% CI)	n (%)	n (%)	aOR (95% CI)
Urinary tract infection	aBLR	n (%)	n (%)	aOR (95% CI)	n (%)	n (%)	aOR (95% CI)	n (%)	n (%)	aOR (95% CI)
Other infections	aBLR	n (%)	n (%)	aOR (95% CI)	n (%)	n (%)	aOR (95% CI)	n (%)	n (%)	aOR (95% CI)
**Antimicrobial use and resistance**										
3rd generation cephalosporin resistance	aBLR	n (%)	n (%)	aOR (95% CI)	n (%)	n (%)	aOR (95% CI)	n (%)	n (%)	aOR (95% CI)
Antimicrobial use during first 7 days*		DDD	DDD	-	DDD	DDD	-	DDD	DDD	-

mRS, modified Rankin Scale. Data are n (%) or median [IQR]. aOR: adjusted odds ratio. Comparison by adjusted ordinal logistic regression (aOLR) or binary logistic regression (aBLR).

* Converted to units of defined daily doses according to the classification of the WHO Anatomical Therapeutic Chemical Classification System with Defined Daily Doses (DDD) Index;

Supplement Table 4. Overview of safety

	Paracetamol	Control	P	Metoclopramide	Control	P	Ceftriaxone	Control	P
Infections diagnosed by physician	n (%)	n (%)		n (%)	n (%)		n (%)	n (%)	
- Pneumonia	n (%)	n (%)		n (%)	n (%)		n (%)	n (%)	
- Urinary tract infection									
- Other infection	n (%)	n (%)		n (%)	n (%)		n (%)	n (%)	
Pneumonia diagnosed by an independent adjudication committee	n (%)	n (%)		n (%)	n (%)		n (%)	n (%)	
Clostridium difficile infection of the gastro-intestinal tract	n (%)	n (%)		n (%)	n (%)		n (%)	n (%)	
Infection with a ceftriaxone resistant micro-organism	n (%)	n (%)		n (%)	n (%)		n (%)	n (%)	
Liver function disturbance or liver failure	n (%)	n (%)		n (%)	n (%)		n (%)	n (%)	
Allergic or hypersensitivity reaction	n (%)	n (%)		n (%)	n (%)		n (%)	n (%)	
**Other SAEs**:									
Total amount of SAEs	n (%)	n (%)		n (%)	n (%)		n (%)	n (%)	

Total amount of related SAEs (SARs or SUSARs)	n (%)	n (%)	n (%)	n (%)	n (%)	n (%)
Total amount of SUSARs	n (%)	n (%)	n (%)	n (%)	n (%)	n (%)

Data are n (%). SAE, Severe Adverse Event; SAR, Severe Adverse Reaction; SUSAR, Severe Unexpected Serious Adverse Reaction. Comparisons made by binary logistic regression**.**

Supplement Figure 1 a/b/c. Kaplan Meier of death for each intervention

Declarations

Ethics approval and consent to participate

The primary Ethics approval for the PRECIOUS trial has been provided by the Medical Ethics Committee of the University Medical Center Utrecht, Utrecht, the Netherlands (NL54304.041.13). We have obtained informed consent from all participants in the study

Consent for publication: Not applicable.

Availability of data and materials

The details of the study protocol have been published earlier (20). After publication of the trial, to promote the independent re-use of PRECIOUS data, a coded dataset will be made available in a public data repository within 18 months of the final follow-up of the last patient. Coded data will also be included in VISTA.

Competing interests

HBvdW served as a consultant to Boehringer Ingelheim, Bayer and LivaNova. PMB has served on advisory boards with DiaMedica, Moleac, Nestle, Phagenesis, Platelet Solutions and Sanofi

Funding

PRECIOUS is funded by the European Union’s Horizon, 2020 research and innovation programme (grant no. 634809).

Authors' contributions

HBvdW is the PRECIOUS coordinating investigator. All authors contributed to the design of the statistical analysis. JdJ wrote the first draft of the manuscript and all author authors reviewed the manuscript carefully. All authors read and approved the final version of the manuscript.

Acknowledgements: None.

List of abbreviations

BI	Barthel Index
BLR	Binary logistic regression
BP	Blood Pressure
CCMO	Centrale Commissie Mensgebonden Onderzoek
CONSORT	Consolidated Standards of Reporting Trials
CPHR	Cox proportional hazards regression
DIM	Difference in means
ESBL	Extended-Spectrum Beta-Lactamase
EQ-5D-5L	EuroQol 5D-5L
EQ-VAS	EQ-visual analogue scale
GCP	Good Clinical Practice
DDD	Daily Defined Dose
DSMB	Data and Safety Monitoring Board
HR	Hazard Ratio
ICH	International Conference on Harmonisation
IMP	Investigational Medicinal Product
IQR	Interquartile Range
MD	Medical Doctor
MLR	Multiple linear regression
MoCA	Montreal Cognitive Assessment
mRS	Modified Rankin Scale
NIHSS	National Institutes of Health Stroke Scale
NSTU	Nottingham Stroke Trial Unit
OLR	Ordinal logistic regression
OR	Odds ratio

PhD	Doctor of Philosophy
PCR	Polymerase chain reaction
PRECIOUS	PREvention of Complications to Improve OUtcome in elderly patients with acute Stroke
PROBE	Prospective randomised open blinded end-point
SAE	Serious Adverse Event
SAP	Statistical Analysis Plan
SAR	Serious Adverse Reaction
SD	Standard Deviation
SUSAR	Suspected Unexpected Serious Adverse Reaction
UMC	University Medical Center
UNOTT	University of Nottingham
VISTA	Virtual International Stroke Trials Archive
WHO	World Health Organisation

Supplementary appendix

List of PRECIOUS partners

**Affiliation**	**Investigator(s)**
Department of Neurology and Neurosurgery, Brain Center, University Medical Center Utrecht, Utrecht University, Utrecht, The Netherlands.	Hendrik Reinink, Jeroen C de Jonge, H Bart van der Worp
Stroke Trials Unit, Division of Clinical Neuroscience, University of Nottingham, Nottingham, United Kingdom	Philip M Bath
Department of Neurology, Academic Medical Center, Amsterdam Neuroscience, Amsterdam, The Netherlands	Diederik van de Beek
Department of Internal Medicine, Oslo, University Hospital, Oslo, Norway	Eivind Berge
Department of Neurology and Stroke Unit, ASST di Mantova, Mantua, Italy.	Alfonso Ciccone
Department of Neurology, University of Debrecen, Debrecen, Hungary	Laszlo Csiba
European Clinical Research Infrastructure Network (ECRIN), Paris, France	Jacques Demotes
Department of Neurology, Erasmus MC, University Medical Center, Rotterdam, The Netherlands	Diederik W Dippel
Department of Neurology and Neurosurgery, University of Tartu, Tartu, Estonia	Janika Korv

2nd Department of Neurology, Institute of Psychiatry and Neurology, Warsaw , Poland	Iwona Kurkowska-Jastrzebska
Institute of Cardiovascular and Medical sciences, University of Glasgow, Glasgow, United Kingdom	Kennedy R Lees
Division of Clinical Neurosciences, Centre for Clinical Brain Sciences, University of Edinburgh, Edinburgh, United Kingdom	Malcolm R Macleod
Department of Medicine, Larissa University Hospital, University of Thessaly, Larissa, Greece	George Ntaios
Stroke Alliance for Europe (SAFE), Brussels, Belgium	Gary Randall
Department of Neurology, Center for Clinical Neurosciences, University Medical Center Hamburg-Eppendorf, Hamburg, Germany.	Götz Thomalla

References

1. Kumar S, Selim MH, Caplan LR. Medical complications after stroke. Lancet Neurol. 2010;9(1):105–18.

2. Hesse K, Fulton RL, Abdul-Rahim AH, Lees KR, VISTA Collaborators. Characteristic adverse events and their incidence among patients participating in acute ischemic stroke trials. Stroke. 2014;45(9):2677–82.

3. Westendorp, Willeke; Vermeij, Jan-Dirk; Hilkens, Nina A; Brouwers, Matthijs C; Algra Ale; van der Worp, H Bart; Dippel, Diederik WJ; van de Beek, Diederik; Nederkoorn PJ. Development and internal validation of a prediction rule for post-stroke infection and post-stroke pneumonia in acute stroke patients. Eur Stroke J. 2018;0(0):1–9.

4. Westendorp WF, Nederkoorn PJ, Vermeij J-D, Dijkgraaf MG, de Beek D van. Post-stroke infection: A systematic review and meta-analysis. BMC Neurol. 2011;11(1):110.

5. den Hertog H, van der Worp B, van Gemert M, Dippel D. Therapeutic hypothermia in acute ischemic stroke. Expert Rev Neurother. 2007;7(2):155–64.

6. Greer DM, Funk SE, Reaven NL, Ouzounelli M, Uman GC. Impact of Fever on Outcome in Patients With Stroke and Neurologic Injury: A Comprehensive Meta-Analysis. Stroke. 2008;39(11):3029–35.

7. Saini M, Saqqur M, Kamruzzaman A, Lees KR, Shuaib A, VISTA Investigators. Effect of hyperthermia on prognosis after acute ischemic stroke. Stroke. 2009;40(9):3051–9.

8. Martino R, Foley N, Bhogal S, Diamant N, Speechley M, Teasell R. Dysphagia After Stroke: Incidence, Diagnosis, and Pulmonary Complications. Stroke. 2005;36(12):2756–63.

9. Geurts M, Scheijmans FE V, van Seeters T, Biessels GJ, Kappelle LJ, Velthuis BK, et al. Temporal profile of body temperature in acute ischemic stroke: relation to infarct size and outcome. BMC Neurol. 2016;16(1):233.

10. Cohen DL, Roffe C, Beavan J, Blackett B, Fairfield CA, Hamdy S, et al. Post-stroke dysphagia: A review and design considerations for future trials. Int J Stroke. 2016;11(4):399–411.

11. L.J. W, P. S, S. H, D.G. S, D.L. C, C. R, et al. Route of Feeding as a Proxy for Dysphagia After Stroke and the Effect of Transdermal Glyceryl Trinitrate: Data from the Efficacy of Nitric Oxide in Stroke Randomised Controlled Trial. Transl Stroke Res. 2018;9(2):120–9. l

12. den Hertog HM, van der Worp HB, van Gemert HMA, Algra A, Kappelle LJ, van Gijn J, et al. An early rise in body temperature is related to unfavorable outcome after stroke: data from the PAIS study. J Neurol. 2011;258(2):302–7.

13. Westendorp WF, Vermeij J-D, Vermeij F, Den Hertog HM, Dippel DW, van de Beek D, et al. Antibiotic therapy for preventing infections in patients with acute stroke. Cochrane Database Syst Rev. 2012: CD008530.

14. den Hertog HM, van der Worp HB, van Gemert HMA, Algra A, Kappelle LJ, van Gijn J, et al. The Paracetamol (Acetaminophen) In Stroke (PAIS) trial: a multicentre, randomised, placebo-controlled, phase III trial. Lancet Neurol. 2009;8(5):434–40.

15. Warusevitane A, Karunatilake D, Sim J, Lally F, Roffe C. Safety and effect of metoclopramide to prevent pneumonia in patients with stroke fed via nasogastric tubes trial.

Stroke. 2015;46(2):454–60.

16. Westendorp WF, Vermeij J-D, Zock E, Hooijenga IJ, Kruyt ND, Bosboom HJLW, et al. The Preventive Antibiotics in Stroke Study (PASS): a pragmatic randomised open-label masked endpoint clinical trial. Lancet. 2015;385(9977):1519–26.

17. Kalra L, Irshad S, Hodsoll J, Simpson M, Gulliford M, Smithard D, et al. Prophylactic antibiotics after acute stroke for reducing pneumonia in patients with dysphagia (STROKE-INF): a prospective, cluster-randomised, open-label, masked endpoint, controlled clinical trial. Lancet. 2015;386(10006):1835–44.

18. Steiner T, Al-Shahi Salman R, Beer R, Christensen H, Cordonnier C, Csiba L, et al. European Stroke Organisation (ESO) guidelines for the management of spontaneous intracerebral hemorrhage. Int J Stroke. 2014;9(7):840–55.

19. Ntaios G, Dziedzic T, Michel P, Papavasileiou V, Petersson J, Staykov D, et al. European Stroke Organisation (ESO) guidelines for the management of temperature in patients with acute ischemic stroke. Int J Stroke. 2015;10(6):941–9.

20. Gamble C, Krishan A, Stocken D, Lewis S, Juszczak E, Doré C, et al. Guidelines for the content of statistical analysis plans in clinical trials. JAMA - J Am Med Assoc. 2017;318(23):2337–43.

21. Reinink H, de Jonge JC, Bath PM, van de Beek D, Berge E, Borregaard S, et al. PRECIOUS: PREvention of Complications to Improve OUtcome in elderly patients with acute Stroke. Rationale and design of a randomised, open, phase III, clinical trial with blinded outcome assessment. Eur Stroke J. 2018;3(3):291–8.

22. Lees KR, Bath PMW, Schellinger PD, Kerr DM, Fulton R, Hacke W, et al. Contemporary Outcome Measures in Acute Stroke Research. Stroke. 2012;43(4):1163–70.

23. McArthur KS, Johnson PCD, Quinn TJ, Higgins P, Langhorne P, Walters MR, et al. Improving the Efficiency of Stroke Trials. Stroke. 2013;44(12):3422–8.

24. Bath PMW, Lees KR, Schellinger PD, Altman H, Bland M, Hogg C, et al. Statistical analysis of the primary outcome in acute stroke trials. Stroke. 2012;43(4):1171–8.
