# Supplementary material for: Prevention of infections and fever to improve outcome in older patients with acute stroke (PRECIOUS): a randomised, open, phase III, multifactorial, clinical trial with blinded outcome assessment
Source: Lancet Reg Health Eur. 2023 Dec 1;36:100782. doi: 10.1016/j.lanepe.2023.100782 (PMC10698669; doi:10.1016/j.lanepe.2023.100782)
Supplement: TLRHEUROPE-D-23-00354 study protocol.pdf [file mmc3.pdf]

Study protocol

PRECIOUS: PREvention of Complications to Improve OUtcome in elderly patients with acute Stroke. A randomised, open, phase III, clinical trial with blinded outcome assessment

For UMC Utrecht:

H. Bart van der Worp
Department of Neurology & Neurosurgery
Brain Center Rudolf Magnus
University Medical Center Utrecht
Heidelberglaan 100
3584 CX Utrecht
The Netherlands
P 088 7571833
F 030 2542100
E h.b.vanderworp@umcutrecht.nl

COMPLIANCE

The study will be conducted in compliance with this protocol, International Conference on Harmonisation (ICH) Good Clinical Practice (GCP), and applicable National and International regulatory requirements (including Declaration of Helsinki).

CONFIDENTIALITY STATEMENT

The information in this document contains information that is privileged or confidential and may not be disclosed unless such disclosure is required by applicable law and regulations. In any event, persons to whom the information is disclosed must be informed the information is privileged or confidential and may not be further disclosed by them. The same applies to photocopying the document or any part of it.

SIGNATURE PAGE

**Sponsor**<br><br>Professor Nicolette C. Notermans<br>Professor and Chairman of Neurology<br>Department of Neurology and Neurosurgery<br>Director Research and Education<br>Brain Center Rudolf Magnus<br>University Medical Center Utrecht<br>Heidelberglaan 100<br>3584 CX Utrecht<br>The Netherlands	Date:<br><br><br><br>Signature:
**Chief investigator**<br><br>H. Bart van der Worp<br>Department of Neurology & Neurosurgery<br>Brain Center Rudolf Magnus<br>University Medical Center Utrecht<br>Heidelberglaan 100<br>3584 CX Utrecht<br>The Netherlands	Date:<br><br><br><br>Signature:

OVERVIEW

**Protocol title**	PRECIOUS: PREvention of Complications to Improve OUtcome in elderly patients with acute Stroke. A randomised, open, phase III, clinical trial with blinded outcome assessment
**Protocol ID**	ToL54304
**Short title**	PRECIOUS trial
**Sponsor**	University Medical Center Utrecht
**EudraCT number**	2015-003179-32
**Version**	Version 4.1
**Date**	16 December 2020
**Funding**	European Union's Horizon 2020 Research and Innovation Programme under grant agreement No 634809
**Amendment history**	Protocol amendments:<br>- Amendment 1: Dose reduction of metoclopramide (protocol version 3.0)<br>- Amendment 2: IMP Labelling (protocol version 3.1)<br>- Amendment 3: Inclusion period of 24 hours (protocol version 4.0)<br>- Amendment 4: Extension of end date (protocol version 4.1)<br><br>Please see section number 16 'summary of changes' for a more detailed overview of each amendment.

CONTACT INFORMATION

**Chief Investigator**	H. Bart van der Worp MD PhD<br>Department of Neurology & Neurosurgery<br>Brain Center Rudolf Magnus<br>University Medical Center Utrecht<br>Heidelberglaan 100<br>3584 CX Utrecht<br>The Netherlands<br>h.b.vanderworp@umcutrecht.nl
**Sponsor**	University Medical Center Utrecht<br>Heidelberglaan 100<br>3584 CX Utrecht<br>The Netherlands
**Monitoring Organisation**	ECRIN-ERIC<br>Paris BioPark<br>5 rue Watt<br>75013 Paris<br>France
**Pharmacovigilance**	Dr. Saskia Borregaard<br>CTC-North GmbH & Co. KG<br>At the University Medical Center Hamburg-Eppendorf<br>Martinistrasse 64<br>20251 Hamburg<br>Germany
**Independent Expert(s)**	For the Netherlands:<br>Dr. W. Ludo van der Pol<br>Department of Neurology & Neurosurgery<br>Brain Center Rudolf Magnus<br>University Medical Center Utrecht<br>Heidelberglaan 100<br>3584 CX Utrecht<br>The Netherlands<br>w.l.vanderpol@umcutrecht.nl
**Data management**	Joost J. Schotsman<br>Department of Biostatistics and Research Support<br>Julius Center<br>University Medical Center Utrecht<br>Heidelberglaan 100<br>3584 CX Utrecht<br>The Netherlands
**Statistical analysis**	Professor Philip M.W. Bath BSc MB BS MD FRCPath FRCP FESO<br>Division of Clinical Neuroscience<br>Faculty of Medicine & Health Sciences<br>University of Nottingham<br>Room B51 Clinical Sciences Building<br>Nottingham City Hospital

	Hucknall Road<br>Nottingham NG5 1PB<br>United Kingdom
**Data & Safety Monitoring Board**	Professor Dr. med. Dr. h.c. Dipl. Psych. Werner Hacke (Chair)<br>Neurologische Klinik<br>Universität Heidelberg<br>Im Neuenheimer Feld 400<br>69120 Heidelberg<br>Germany
**Ethical Advisory Board**	Professor Kennedy R. Lees MD FRCP FESO FRSE (Chair)<br>Institute of Cardiovascular and Medical Sciences<br>University of Glasgow & Western Infirmary<br>44 Church Street<br>Glasgow G11 6NT<br>United Kingdom
**Central Laboratory [only for analysis of rectal swabs in a subpopulation of patients]**	Prof. dr. Diederik van de Beek MD PhD<br>Netherlands Reference Laboratory for Bacterial Meningitis<br>Subsection Neuroinfections<br>Location L1-114<br>Department of Medical Microbiology<br>P.O. Box 22660<br>1100 DD Amsterdam<br>The Netherlands
**Central Pharmacy**	NA

LIST OF ABBREVIATIONS

ADL	Activities of Daily Living
AE	Adverse Event
ALP	Alkaline Phosphatase
ALT	Alanine Aminotransferase
AST	Aspartate Aminotransferase
ATC	Anatomical Therapeutic Chemical
BI	Barthel Index
CONSORT	Consolidated Standards of Reporting Trials
CRP	C-Reactive Protein
CTC-North	Clinical trial Center North
CTCAE	Common Terminology Criteria for Adverse Events
DALY	Disability-Adjusted Life-Year
DAMOCLES	Data Monitoring Committees: Lessons, Ethics, Statistics
DSMB	Data and Safety Monitoring Board
ECG	Electrocardiogram
eCRF	Electronic Case Report Form
EAB	Ethical Advisory Board
ECRIN-ERIC	European Clinical Research Infrastructure Network-European Research Infrastructure Consortium
ESO	European Stroke Organisation
EU	European Union
EudraCT	European Union Drug Regulating Authorities Clinical Trials
EQ-5D-5L	EuroQoL 5-dimensions 5-level
ESBL	Extended-Spectrum Beta-Lactamase
GCP	Good Clinical Practice
GGT	Gamma-Glutamyl Transferase
ID	Identification
ICF	Informed Consent Form
ICH	International Conference on Harmonisation
IEC	Independent Ethics Committee
IMP	Investigational Medicinal Product
ISF	Investigator Site File
ISRCTN	International Standard Randomized Controlled Trial Number
LLT	Lowest Level Term
MedDRA	Medical Dictionary for Regulatory Activities
MoCA	Montreal Cognitive Assessment
mRS	Modified Rankin Scale
NADH	Nicotinamide Adenine Dinucleotide
NFU	Nederlandse Federatie van Universitair Medische Centra (Dutch Federation of University Medical Centres)
NIHSS	National Institutes of Health Stroke Scale
NSAID	Non-steroid Anti-Inflammatory Drug
PBP	Penicillin-Binding Protein
PCR	Polymerase Chain Reaction
PROBE	Prospective, Randomised, Blinded Endpoint
QA	Quality Assurance
QC	Quality Control

RA	Regulatory Authorities
RO	Research Online
SAE	Serious Adverse Event
SAP	Statistical Analysis Plan
SAR	Serious Adverse Reaction
SmPC	Summary of Product Characteristics
SOC	System Organ Class
SUSAR	Suspected Unexpected Serious Adverse Reaction
TMF	Trial Master File
WHO	World Health Organisation

SYNOPSIS

**Protocol ID**	ToL54304
**Title**	PRECIOUS: PREvention of Complications to Improve OUtcome in elderly patients with acute Stroke. A randomised, open, phase III, clinical trial with blinded outcome assessment
**Short title**	PRECIOUS trial
**EudraCT number**	2015-003179-32
**Introduction**	Every year, 1.3 million Europeans have a first stroke. One fifth to one third of stroke patients die in the first month after stroke, and one third remain dependent on the help of others. The annual costs for stroke care in Europe have been estimated at €64.1 billion. Stroke incidence increases almost exponentially with age, and the personal, societal, and economic burden of stroke is therefore largely driven by its frequent occurrence in the elderly. Elderly patients are at the highest risk of complications after stroke, such as infections and fever. These complications are strongly and independently associated with a higher risk of death or dependency. The risk of developing these complications can be reduced by very simple, safe, and cheap measures, such as metoclopramide for the management of dysphagia, antibiotics for the prevention of infections, and paracetamol for the prevention of fever, but it is uncertain whether these measures also improve functional outcome.
**Study Overview**	PRECIOUS will assess whether a pharmacological strategy to prevent common complications in elderly patients with acute stroke is more effective at reducing the risk of death and improving functional outcome than current clinical practice - recommended in guidelines - of waiting until these complications are manifest before initiating treatment.
**Planned Number of subjects**	3800
**Primary Objective**	To assess whether prevention of aspiration, infections, or fever with metoclopramide, ceftriaxone, paracetamol, or any combination of these in the first 4 days after stroke onset improves functional outcome at 90 days in elderly patients with acute stroke.
**Secondary Objectives**	▪ To assess the effect of prevention of aspiration, infections, or fever in the first days after stroke onset with metoclopramide, ceftriaxone, paracetamol, or any combination of these, on the following outcomes at 90 days:<br>▪ Death<br>▪ Death or dependency<br>▪ Quality of life<br>▪ Cognition<br>▪ Costs<br>▪ To detect specific populations classified by age, sex, stroke severity, body temperature, co-morbidities, and geographic region, in which the proposed treatments are particularly effective or not effective.<br>▪ To assess the effects of prophylactic treatment with ceftriaxone on antimicrobial resistance and the occurrence of infections with *Clostridium difficile*.

**Primary Outcome**	The primary outcome measure is the score on the modified Rankin Scale (mRS) at 90 days (± 14 days).
**Secondary Outcome**	At 7 days (± 1 day) or at discharge, if earlier:<br>▪ Infections in the first 7 days (± 1 day; frequency, type, and C. difficile overgrowth syndrome). Infections will be categorised as diagnosed by the clinician, and as judged by an independent adjudication committee (masked to treatment allocation);<br>▪ 3rd generation cephalosporin resistance in the first 7 days (± 1 day), detected as part of routine clinical practice;<br>▪ Antimicrobial use during the first 7 days (± 1 day), converted to units of defined daily doses according to the classification of the WHO Anatomical Therapeutic Chemical Classification System with Defined Daily Doses Index;<br>▪ In a subgroup of patients: presence of Extended-Spectrum Beta-Lactamase (ESBL)-producing bacteria as detected by PCR in a rectal swab.<br>At 90 days (± 14 days):<br>▪ Death;<br>▪ Unfavourable functional outcome, defined as mRS 3 to 6;<br>▪ Disability assessed with the score on the Barthel Index (BI);<br>▪ Cognition assessed with the Montreal Cognitive Assessment (MoCA);<br>▪ Quality of life assessed with the EuroQol 5D-5L (EQ-5D-5L);<br>▪ Home time: number of nights among the first 90 since stroke onset that are spent in the patient's own home or a relative's home over the first 90 days;<br>▪ Patient location over first 90 days (± 14 days): hospital; rehabilitation service; chronic nursing facility; home.<br>▪ SAEs in the first 7 days.
**Inclusion Criteria**	1. a clinical diagnosis of acute ischaemic stroke or intracerebral haemorrhage, confirmed with CT or MRI scan. A normal CT scan is considered compatible with ischaemic stroke;<br>2. a score on the National Institutes of Health Stroke Scale (NIHSS) ≥ 6, indicating moderately severe to severe stroke;<br>3. age 66 years or older;<br>4. the possibility to start treatment within 24 hours of symptom onset<br>5. written informed consent.
**Exclusion Criteria**	1. Active infection requiring antibiotic treatment, as judged by the treating physician;<br>2. Pre-stroke score on the mRS ≥4<br>3. Death appearing imminent at the time of assessment.<br><br>In addition, patients will be excluded from participation in the trial treatment strata for any of the following reasons:<br>**For the ceftriaxone stratum:**<br>1. Known hypersensitivity to beta-lactam antibiotics;<br>2. Clinical indication for antibiotic treatment. The use of an antibiotic before screening is not an exclusion criterion.<br>**For the paracetamol stratum:**

	1. Known hypersensitivity to paracetamol or any of the excipients;<br>2. Known severe hepatic insufficiency;<br>3. Chronic alcoholism.<br>4. Clinical indication for the use of paracetamol. Incidental use of paracetamol before screening is not an exclusion criterion.<br><br>**For the metoclopramide stratum:**<br>1. Hypersensitivity to metoclopramide or to any of the excipients;<br>2. Gastrointestinal haemorrhage, mechanical obstruction or gastro-intestinal perforation for which the stimulation of gastrointestinal motility constitutes a risk;<br>3. Confirmed or suspected pheochromocytoma;<br>4. History of neuroleptic or metoclopramide-induced tardive dyskinesia;<br>5. Epilepsy;<br>6. Parkinson's disease;<br>7. Use of levodopa or dopaminergic agonists;<br>8. Known history of methaemoglobinaemia with metoclopramide or of NADH cytochrome-b5 deficiency.<br>9. Clinical indication for the use of metoclopramide. Incidental use metoclopramide before screening is not an exclusion criterion.
**Study Design**	International, multi-centre, multi-factorial, randomised, controlled, open-label clinical trial with blinded outcome assessment (PROBE) of metoclopramide, ceftriaxone, paracetamol, or any combination of these, in 3800 elderly patients with acute ischaemic stroke or intracerebral haemorrhage. The trial will be performed according to ICH-GCP principles, the Declaration of Helsinki as most recently amended in 2013, and national and international regulatory requirements.
**Study treatment**	Patients will be randomly allocated in a 2*2*2 factorial design to any combination of open-label oral, rectal, or intravenous metoclopramide (10 mg thrice daily), intravenous ceftriaxone (2000 mg once daily), oral, rectal, or intravenous paracetamol (1000 mg four times daily), or usual care, started within 24 hours after symptom onset and continued for 4 days or until complete recovery or discharge from hospital, if earlier. In patients with moderate to severe renal impairment or with severe hepatic impairment, the dose of metoclopramide is reduced to 5 mg thrice daily, and in patients with end-stage renal disease to 2.5 mg thrice daily. Allocation will be based on proportional minimisation through a web-based allocation service. Investigators will have the opportunity to censor a single specific randomisation stratum in a specific patient before randomisation, for example in case of an allergy to one of the interventions.
**Statistical Analysis**	The primary effect estimate will be the difference in the mean mRS scores assessed using multiple regression, and will be expressed as a mean difference with 95% confidence interval. The statistical analyses will be performed according to the intention-to-treat principle and adjusted for relevant baseline characteristics and treatment allocation for the other two strata of the trial.

**Planned schedule**	First patient included by May 2016; final follow-up of the last patient by November 2022. Once enrolled, patients will receive trial treatment for four days. Data collection will take place daily up to and including day 7 (± 1 day, or until discharge, if earlier) and at day 90 (± 14 days) after randomisation.

SCHEDULE OF ACTIVITIES

ASSESSMENT/ACTIVITY	SCREENING	DAY 1	DAY 2	DAY 3	DAY 4	DAY 5	DAY 6	DAY 7	END OF TRIAL
**Visit Name**	**Screening**	**V1**	**V2**	**V3**	**V4**	**V5**	**V6**	**V7**	**FU**
**Day(s)[a]**	0	1	2	3	4	5	6	7	90
**Visit Window**								± 1 day	± 14 days
Informed Consent[b]	X								
Randomisation	X								
Demographics[c]	X								
Medical History[d]	X								
Pre-stroke mRS[e]	X								
Concurrent medication[f]	X	X	X	X	X	X	X	X	
Date and time of stroke onset[e]	X								
NIHSS[g]	X								
Vital signs[h]	X	X	X	X	X	X	X	X	
Laboratory[i]	X								
Imaging[j]	X								
Lesion location[k]	X								
Stroke type[l]	X								
Other stroke treatment[m]	X								
Trial treatment[n]		X	X	X	X				
Infections[o]	X	X	X	X	X	X	X	X	
Rectal swabs[p]	X							X	
Feeding[q]	X	X	X	X	X	X	X	X	
Treatment restrictions[r]	X	X	X	X	X	X	X	X	
mRS[s]								X	X
Patient location[t]	X	X	X	X	X	X	X	X	X
MoCA[u]									X
Barthel Index[v]									X
EuroQoL[w]									X
AE & SAE[x]		X	X	X	X	X	X	X	

a. Day 1 starts immediately after randomisation; day 2 at 24 hours after randomisation; etc.;
b. From the patient or a legal representative, or after approval of an independent physician, in accordance with local and national regulation;
c. Paragraph 6.1: age, sex;
d. Paragraph 6.1: atrial fibrillation; diabetes mellitus; hypertension;
e. Paragraph 6.1;
f. Paragraphs 6.1 and 6.7: use of any antiemetic, antibiotic, or antipyretic drug. Aspirin in any formulation and in a daily dose of up to 300 mg is NOT considered an antipyretic drug;
g. Paragraph 6.2;
h. Paragraphs 6.1 and 6.4: blood pressure; pulse; and body temperature at baseline and at 12-hour (± 3 hours) intervals during the hospital phase if assessed as part of routine clinical care;
i. Paragraph 6.5: If assessed as part of routine clinical care: leukocyte count and differential; serum glucose; glomerular filtration rate; C-reactive protein (CRP); alkaline phosphatase (ALP); gamma-glutamyl transferase (GGT); alanine aminotransferase (ALT); and aspartate aminotransferase (AST);
j. Paragraph 6.6: unenhanced CT or MRI as part of routine clinical care; results of chest X-ray if performed as part of routine clinical care;
k. Paragraph 6.1: expected or confirmed location of the lesion (left or right hemisphere; posterior fossa);
l. Paragraph 6.1: ischaemic stroke or intracerebral haemorrhage;
m. Paragraph 6.1: Intravenous thrombolysis or intra-arterial treatment;
n. Paragraph 6.7;
o. Paragraph 6.8: Infections, use of antibiotics, and selection of bacteria with increased antimicrobial resistance during the first 7 days (or until discharge, if earlier);
p. Paragraph 6.8: Only in patients who participate in the relevant substudy. If the patient is discharged before day 7, a swab should be taken on the day of discharge;
q. Paragraph 6.9: Feeding classified as 1. Normal food; 2. Oral, soft or fluids only; 3. Nasogastric tube; 4. Percutaneous endoscopic gastrostomy (PEG); 5. Intravenous only; 6. None. The assessment at baseline indicates the method of food intake before stroke;
r. Paragraph 6.10: Treatment restrictions classified as 1. Do not resuscitate; 2. Do not intubate and ventilate; 3. Withholding other treatments that may prolong life; 4. Withholding food; 5. Withholding fluids; and 6. Palliation;
s. Paragraph 6.3: Only the assessment at 90 days will be recorded using a digital video camera;
t. Paragraph 6.11: Location of the patient at noon of the relevant day;
u. Paragraph 6.12;
v. Paragraph 6.13;
w. Paragraph 6.14;
x. Chapter 9. Recording of all AEs grade 3 to 5 will begin after randomisation and end on day 7. Any SAE occurring during this time frame has to be reported within 24 hours of Investigator's first awareness about the event, with the exception of expected SAE as indicated in paragraph 9.2.2.

TABLE OF CONTENTS

SIGNATURE PAGE **2**
OVERVIEW **3**
CONTACT INFORMATION **4**
LIST OF ABBREVIATIONS **6**
SYNOPSIS **9**
SCHEDULE OF ACTIVITIES **13**
1. INTRODUCTION AND RATIONALE **18**
2. OBJECTIVES & OUTCOMES **24**
2.1. Study objectives *24*
2.2. Study outcomes *25*
2.2.1. Primary outcome 25
2.2.2. Secondary outcomes 25
3. STUDY DESIGN **26**
4. STUDY POPULATION **27**
4.1. Subject Selection *27*
4.2. Inclusion Criteria *27*
4.3. Exclusion Criteria *27*
4.4. Sample Size *28*
5. TREATMENT OF SUBJECTS **29**
5.1. Study phases *29*
5.1.1. Screening Phase 29
5.1.2. Hospital Phase 29
5.1.3. Follow-up Phase 29
5.2. Treatment allocation *29*
5.3. Description of investigational medicinal products *30*
5.3.1. Metoclopramide 30
5.3.2. Ceftriaxone 32
5.3.3. Paracetamol 33
5.4. Pregnancy and contraception *34*
5.5. Concomitant medications and treatments *34*
6. STUDY PROCEDURES **35**
6.1. Baseline assessment *35*
6.2. National Institutes of Health Stroke Scale (NIHSS) *35*
6.3. modified Rankin Scale (mRS) *35*
6.4. blood pressure, pulse, and body temperature *36*
6.5. laboratory tests *36*
6.6. imaging *36*
6.7. medication *36*

6.8. *Infections and resistance* 36
6.9. *Feeding* 37
6.10. *Treatment restrictions* 37
6.11. *patient location* 37
6.12. *indirect societal costs* 37
6.13. *Montreal Cognitive Assessment (MoCA)* 38
6.14. *Barthel index (BI)* 38
6.15. *EuroQol (EQ-5D-5L)* 38
6.16. *safety* 38
7. STATISTICAL METHODS & CONSIDERATIONS 39
7.1. *Statistical analysis Plan* 39
7.2. *Sample Size* 39
7.3. *Analysis of Primary Endpoint* 39
7.4. *Analysis of Secondary Endpoints* 40
7.5. *Subgroup analyses* 40
7.6. *Interim analyses* 40
7.7. *Procedures for missing, unused, and spurious data* 40
7.8. *definitions of populations analysed* 40
8. DATA COLLECTION, HANDLING AND RECORD RETENTION 42
8.1. *Recording of Data – Source Documentation and Electronic Case Report Forms* 42
8.2. *Data Quality Assurance* 42
8.3. *Data Management* 43
8.4. *Record Retention* 43
8.5. *Monitoring the Study* 43
8.6. *Audits and Inspections* 44
8.7. *Confidentiality of Study Documents and Subject Records* 44
9. SAFETY AND AES 46
9.1. *Safety Definition* 46
9.1.1. Adverse Event (AE) 46
9.1.2. Serious Adverse Event (SAE) and Serious Adverse Reaction (SAR) 46
9.1.3. Suspected Unexpected Serious Adverse Reactions (SUSARs) 46
9.1.4. AE Severity 46
9.1.5. Relatedness of AE to IMP 47
9.1.6. Outcome categorisation 47
9.2. *Documentation and reporting procedures* 48
9.2.1. Reporting of AEs and SAEs 48
9.2.2. Reporting SUSARS 49
10. END OF STUDY, SUBJECT WITHDRAWAL AND PREMATURE TERMINATION 50
10.1. *End of Study* 50

10.2. Subject Discontinuation or Withdrawal ... 50

10.3. Replacement of Subject ... 51

10.4. Premature Termination of Study ... 51

10.4.1. Criteria for Premature Termination or Suspension of the study ... 51

10.4.2. Criteria for Premature Termination or Suspension of investigational Sites ... 51

10.4.3. Procedures for Premature Termination or Suspension of study or Investigational Sites ... 51

11. DEVIATION & VIOLATION ... 52

11.1. Deviation ... 52

11.2. Violation ... 52

11.3. Follow-up of Deviations and Violations ... 52

12. ETHICAL CONSIDERATIONS & PROTECTION OF HUMAN SUBJECTS ... 53

12.1. Regulation Statement ... 53

12.2. Regulatory Authority/IECs ... 53

12.3. Informed Consent ... 53

12.4. Amendments ... 54

12.5. End of Study report ... 54

12.6. Compensation for Injury ... 54

12.7. Incentives ... 54

13. PUBLICATION ... 55

14. REFERENCES ... 56

15. APPENDICES ... 60

Appendix 1 – Common Terminology Criteria for AEs (CTCAE) version 4.03 ... 60

Appendix 2 – National Institutes of Health Stroke Scale (NIHSS) ... 61

Appendix 3 – modified Rankin Scale (mRS) ... 65

Appendix 4 – Montreal Cognitive Assessment (MoCa) ... 66

Appendix 5 – Barthel Index (BI) ... 67

Appendix 6 – EuroQoL ... 69

Appendix 7 – Known side effects of the study medication ... 72

Appendix 8 – common (potentially) serious complications after stroke ... 74

Appendix 9 – protocol signature sheet local principal investigator ... 76

16. SUMMARY OF CHANGES ... 77

16.1. Amendment 1 ... 77

1. INTRODUCTION AND RATIONALE

Stroke is the second most common cause of death and the third most common cause of loss of disability-adjusted life-years (DALYs) worldwide.[1, 2] Two types of stroke can be distinguished: 80% are ischaemic, most often caused by occlusion of a cerebral artery or arteriole. The other 20% are haemorrhagic, most often caused by a rupture of an artery.[3, 4] Every year, some 1.3 million Europeans have a first stroke.[5] Twenty to 35% of the patients die in the first month after stroke, and around one third remain dependent on the help of others.[6, 7] Even in patients who survive a stroke with minor disabilities, quality of life is often substantially impaired.[8, 9]

Across Europe, stroke accounts for over one million deaths each year.[10] The large majority of these deaths are in elderly patients.[11] However, death rates vary substantially between countries, and are more than three times higher in central and eastern Europe than in northern, southern, and western Europe.[10] Although early stroke mortality rates have decreased in the past two decades, the burden of stroke in terms of the absolute number of people affected, related deaths, and DALYs lost are great and increasing, with most of the burden in low-income and middle-income European countries. If these trends in stroke incidence, mortality, and DALYs continue, by 2030 the numbers of stroke deaths, stroke survivors, and lost DALYs will have doubled.[11]

Stroke and the elderly

Stroke incidence increases almost exponentially with age, and about three quarters of stroke patients are older than 65 years.[7, 12] In addition, advanced age is also the most important predictor of death or dependency after stroke, next to the stroke size and the severity of symptoms.[13, 14] The personal, societal, and economic burden of stroke is therefore largely driven by its frequent occurrence in the elderly.

Acute stroke treatment

Treatment options for patients with ischaemic stroke or intracerebral haemorrhage are limited.[15-18] For patients with ischaemic stroke, aspirin has a small benefit but can be used in a large number of patients;[19] thrombolysis with alteplase has a modest benefit and can be used in a modest number of patients;[20] mechanical thrombectomy with a retrievable stent has a large benefit but can be used in a small number of patients;[21-25] and hemicraniectomy has a large benefit in patients aged 60 years or younger, but can be used in a very small number of patients with large, space-occupying infarction.[26] In addition, its benefit in patients older than 60 years is questionable. In these patients, hemicraniectomy reduces the risk of death, at the cost of a large increase in the risk of long-term dependency in activities of daily living.[27] For all strokes, organised care in a designated stroke unit has a modest effect on outcome and can be used in a large number of patients, but it is not clear which components of this stroke unit care drive the reduction in mortality and long-term dependency.[28] There are no other treatment options for patients with intracerebral haemorrhage of proven benefit, but early blood pressure lowering may improve outcome in a small number of patients.[29]

It is also illustrative that in randomised trials, the risk of a poor outcome (death or dependency) in patients treated with intravenous thrombolysis within 3 hours of symptom onset was still 59%,[20] and in a large observational study 45%.[30] In the highly selected patient populations included in recent trials of endovascular treatment for acute ischaemic stroke, the risk of a poor outcome in patients randomized to the intervention arm ranged from 29 to 67%.[21-25] For these reasons, there is a clear need for additional effective treatment options that can be applied in broad populations of patients with acute stroke.

Complications after stroke

In the first days after stroke, about half of all patients develop complications, including infections and fever. Advanced age and pre-existing co-morbidities increase the risk of developing these events. In addition, patients with severe, disabling strokes are particularly vulnerable.[31, 32] These complications can impede functional recovery, prolong hospital admissions, and are independently associated with an increased risk of death or long-term dependency.[31, 33-37]

Dysphagia is present in a quarter to half of patients with acute stroke, and has been associated with an increased risk of aspiration pneumonia, dehydration, malnutrition, prolonged hospital stays, and death.[31, 37] In a meta-analysis of 87 studies on post-stroke **infection**, involving 137,817 patients, the overall pooled infection rate was 30% (95% CI, 24 to 36%). Rates of pneumonia and urinary tract infection were 10% (95% CI, 9 to 10%) and 10% (95% CI, 9 to 12%), respectively. Infections in general, and pneumonia in particular, were associated with an increased risk of death (OR for pneumonia, 3.6; 95% CI, 2.8 to 4.7).[33] In a prospective observational study, most post-stroke infections occurred in the first three days after hospital admission.[38] **Subfebrile temperatures or fever** are observed in one third to half of the patients in the first days after stroke, and have also consistently been associated with an increased risk of a poor outcome or death.[34-36] The temporal profile of this relationship has remained uncertain. Earlier studies suggested that this relation was limited to the first 12 or 24 hours,[34, 39] but more recent studies have suggested that the risk of a poor outcome is greater with an increase in body temperature during the first 24 hours of admission,[40] or with increased temperatures in the first days after stroke.[36] In an analysis of data of 5305 patients in acute stroke trials, the hazard ratios for poor outcome in relation to increased body temperatures were 1.2 (95% CI, 1.0 to 1.4) at study inclusion, 1.5 (95% CI, 1.2 to 1.9) at one day, 2.0 (95% CI, 1.5 to 2.6) at two days, 2.2 (95% CI, 1.7 to 2.9) at three days, and 2.7 (2.0 to 3.8) at 7 days.[36]

Systematic reviews have strongly suggested that the benefits of stroke unit care are mediated through the prevention and early treatment of complications.[41, 42]

Recommendations in guidelines

American guidelines for the treatment of patients with acute ischaemic stroke advocate screening for dysphagia; the use antibiotics in patients with infections; and antipyretic drugs such as paracetamol in patients with subfebrile temperatures or fever.[16] These recommendations are based not on findings in randomised clinical trials, but on expert opinion, based on the findings in observational studies mentioned above.[33-37] Recent guidelines of the European Stroke Organisation (ESO) concluded that there is insufficient evidence from randomised trials to make strong recommendations on whether, when, and for whom preventive or early antibiotic or antipyretic treatment should be given after ischaemic stroke or intracerebral haemorrhage.[18, 43] The guideline for ischaemic stroke suggested against routine prevention of fever with antipyretics as a means to improve outcome, based on insufficient evidence. The authors called for new randomised trials to allow for better-informed recommendations in the future.[43] Possibly because of this limited evidence, care on the stroke unit is often not consistent with these recommendations.[44] In addition, interventions are usually started late; for example, antibiotics are usually given to the patient several hours to days after they have manifested the first signs of an infection.

Prevention of complications

The risk of some frequent complications after stroke can be reduced by very simple, safe, and cheap measures, such as metoclopramide for the management of dysphagia; antibiotics for the prevention of infections; and paracetamol for the prevention of fever. In generally small randomised controlled trials, preventive treatment with these drugs not only convincingly reduced the risks of aspiration, infections, and fever by one third to one half, but has also been associated with clear trends towards lower case fatality and improved outcomes.[40, 45-47]

In the randomised phase II Metoclopramide to prevent Pneumonia in Stroke patients fed via nasogastric tubes (MAPS) trial of the **management of dysphagia**, 60 patients were randomised to metoclopramide 30 mg daily or placebo within 7 days of stroke onset and within 48 hours of insertion of a nasogastric tube. The trial medication was continued for 21 days or until nasogastric feeds were discontinued. Twenty six (87%) of the patients randomised to placebo and 8 (27%) of the patients randomised to metoclopramide developed pneumonia (adjusted rate ratio, 5.24; 95% CI, 2.43 to 11.27; p<0.001). Twelve (40%) patients in the placebo group and 8 (27%) in the metoclopramide group died within 30 days after start of treatment (adjusted estimate, 1.85; 95% CI, 0.59 to 5.80; p=0.292).[47] The trial did not assess the effect of metoclopramide on functional outcome. There were no differences in adverse events (AEs) between the two groups. Oculogyric crises, dystonic reactions, tardive dyskinesia, and galactorrhoea were not observed.

In a Cochrane meta-analysis of five phase II trial testing the effect of the **prevention of infections** in patients with acute stroke, prophylactic antibiotics reduced the risk of infections from 36% to 22% (RR, 0.58; 95% CI, 0.43 to 0.79) and were associated with a trend towards a reduced risk of dependency: RR, 0.67; 95% CI, 0.32 to 1.43.[45]

In the recent Preventive Antibiotics in Stroke Study (PASS) not included in this meta-analysis, 2550 patients with acute stroke were randomly assigned to intravenous ceftriaxone at a daily dose of 2 g, given every 24 h intravenously for 4 days, or to standard care.[48] Treatment was started within 24 hours of stroke onset. Preventive ceftriaxone reduced the incidence of infections from 7% to 3% (OR, 0.44; 95% CI, 0.30 to 0.65; p<0·0001), mainly through a reduction in the occurrence of urinary tract infections, but did not have a statistically significant benefit on the incidence of pneumonia (3% vs. 2%; OR, 0.67; 95% CI, 0.39 to 1.15; p=0.18). The low incidence of infections may be explained by the inclusion of patients with a generally mild stroke: the median score on the NIHSS was 5 (IQR, 3 to 9). Preventive ceftriaxone did not have an effect on functional outcomes at 90 days: adjusted common odds ratio, 0.95; 95% CI, 0.82 to 1.09; p=0.46. The benefit of ceftriaxone appeared to be greater in patients aged 75 years or older than in younger patients (OR, 0.85; 95% CI, 0.67 to 1.06), but there was no significant interaction between the age groups. Post-hoc analysis suggested that ceftriaxone reduced the risk of a poor outcome in patients treated with alteplase from 40% to 33% (adjusted OR, 0.77; 95% CI 0.61 to 0.99; p=0·04). Preventive treatment with ceftriaxone was safe. Overgrowth infection with *Clostridium difficile* occurred in two patients (0.2%) and infection by a ceftriaxone-resistant microorganism in one patient (0.1%) in the ceftriaxone group; both were not observed in the control group.

In the recent multicentre, cluster-randomised, open-label controlled trial with masked endpoint assessment STROKE-INF,[49] 48 stroke units in the UK (and 1224 patients clustered within the units) were randomly assigned to give either prophylactic antibiotics for 7 days plus standard stroke unit care or standard stroke unit care alone. Patients were eligible for participation if they had stroke and dysphagia, and if prophylactic antibiotic treatment could be started within 48 hours of stroke onset. Eleven units and 7 patients withdrew after randomisation, leaving 1217 patients in 37 units for the intention-to-treat analysis (615 patients in the antibiotics group, 602 in control). Both the type and dose of antibiotic at intervention centres conformed to local antibiotic policies. Of the patients in the intervention group, 78% received amoxicillin or co-amoxiclav together with clarithromycin and 2% a cephalosporin. Prophylactic antibiotics did not affect the incidence of algorithm-defined post-stroke

pneumonia (13% in the antibiotics group vs 10% in the control group; marginal adjusted OR, 1.21; 95% CI, 0.71 to 2.08). Infections unrelated to post-stroke pneumonia were less frequent in the prophylactic antibiotics group (4% vs 7%; OR, 0.55; 95% CI, 0.32 to 0.92; p=0.02). There were no differences in mortality rates between the treatment groups or in the percentage of patients with good functional outcomes (mRS 0 to 2) but the distribution of mRS scores shifted towards worse outcomes at 90 days in the intervention group (adjusted OR, 1.26; 95% CI, 1.01 to 1.57; p=0.039). Treatment with prophylactic antibiotics was not associated with an increase of serious adverse events. Diarrhoea positive for *C. difficile* occurred in two patients (<1%) in the antibiotics group and four (<1%) in the control group.

There are several explanations for the lack of benefit of prophylactic antibiotics in STROKE-INF. First, preventive treatment was started late (up to 48 h after stroke onset). Second, a considerable proportion of patients in the treatment group received a limited number of antibiotic doses, while 34% of the patients in the control group received an antibiotic at least once during the first 7 days. Finally, only a small number of patients were randomised per participating centre over an extended period of time; in a cluster-randomized study, this may induce selection bias decreasing the discriminative power.

Important differences of STROKE-INF as compared to PRECIOUS are its cluster-randomised design, which may lead to selection bias; the time window to start of treatment of 48 hours, which might be too long; and the heterogeneity in the types, doses, and routes of administration of antibiotics. Results of STROKE-INF can therefore not be extrapolated to PRECIOUS. Moreover, the results of PASS and STROKE-INF support the concept that post-stroke pneumonia might also be a respiratory syndrome resulting from complex bacterial, chemical, and immunological causes that might not be prevented by antibiotics alone.[49, 50]

As compared with STROKE-INF, the strengths of PRECIOUS are the randomization of each individual patient; the short time window for start of treatment of 24 hours; the restriction to a single antibiotic in a prespecified dose; and its multifactorial design. Especially the combination of metoclopramide and ceftriaxone may lead to a larger reduction in the risk of pneumonia than antibiotics alone. In addition, the short window for start of treatment may result in inclusion of a high proportion of alteplase-treated patients, a subgroup identified within PASS that may potentially benefit from preventive ceftriaxone treatment.

Prevention of fever has been tested in three small phase II trials[51] and in one published, larger phase III trial: the Paracetamol (Acetaminophen) in Stroke (PAIS) trial, a double-blind, placebo-controlled, randomised clinical trial of 1400 patients with acute stroke.[46] In PAIS, treatment with paracetamol 6 g daily for three days, started within 12 hours of stroke onset, reduced the risk of subfebrile temperatures or fever at 24 hours of start of treatment from 30% to 15%.[40] Patients treated with paracetamol had better functional outcomes as assessed with the mRS at 90 days than those treated with placebo, but this difference was just not statistically significant (adjusted odds ratio (aOR), 1.21; 95% CI, 0.97 to 1.51).[46] In a post-hoc analysis of patients with a baseline body temperature of 37 to 39°C, 40% of the patients in the paracetamol group improved beyond expectation compared with 31% in the placebo group (adjusted OR, 1.43; 95% CI, 1.02 to 1.97), but a stratified Mantel-Haenszel test did not formally indicate heterogeneity in the effect of paracetamol (p=0.12). In PAIS, treatment with high-dose paracetamol was safe. Results of the Paracetamol (Acetaminophen) in Stroke trial 2 (PAIS 2) that was terminated prematurely after inclusion of 300 patients, against a target of 1500, are not known yet. The reason for the premature termination was lack of funding.

Selection of treatments for PRECIOUS

Metoclopramide is the only anti-emetic tested for the prevention of infections in patients with acute stroke and proved to be safe for this indication.[47] The **antibiotics** levofloxacin, penicillin, moxifloxacin, minocycline, mezlocillin, and ceftriaxone have each been used in trials for the prevention of infections in patients with acute stroke, but experience with ceftriaxone is greatest and this has been shown safe in PASS.[45, 52] **Ceftriaxone** is an off-patent, broad-spectrum, antibiotic with proven efficacy against bacteria most frequently causing infection in patients with acute stroke.[52] PRECIOUS will use paracetamol for the prevention of increases in body temperature because this was safe in doses up to 6 gram per day for three days in randomised clinical trials in patients with acute stroke, reduced the risk of subfebrile temperatures or fever by 50%, and was associated with a trend towards an improvement in functional outcome in the PAIS trial. [40, 46] This trial was underpowered to detect a benefit on functional outcome because this was terminated prematurely due to lack of funding after inclusion of 1400 patients, against a target of 2500.[46] One small, randomised trial compared the effects of ibuprofen and paracetamol on body temperature and AEs. As compared with paracetamol, ibuprofen had a smaller effect on body temperature and was associated with a higher risk of gastritis.[53] For PRECIOUS we have selected a maximum daily dose of 4 g to comply with the drug's SmPC.

Duration of treatment

There is no information on the temporal profile of the associations between infections or dysphagia and poor outcome, but the large majority of infections occur during the first week[48] and dysphagia is also most prevalent in this time period.[31, 37] As described under 'complications after stroke' the temporal profile of the association between higher body temperatures and poor outcome is uncertain. Most evidence is available for the first two days after stroke,[34-36 40] but one large study found a strong association during the entire first week.[36] In the previous studies MAPS, PASS, and PAIS trial medication was continued for periods of 21, 4, or 3 days, respectively, and found to be safe.[46-48] Because preventive treatment with ceftriaxone has been shown safe and not associated with an increase in antimicrobial resistance when given for a period of 4 days, and because compliance with the study protocol of this pragmatic trial is likely to be largest when all three study drugs are given for the same duration, all study medication in PRECIOUS will be continued for 4 days, or until discharge, if earlier.

Assessment of risks and benefits

As outlined under 'complications after stroke' about half of all patients develop complications, including infections and fever. Advanced age and pre-existing co-morbidities increase the risk of developing these events. In addition, patients with severe, disabling strokes are particularly vulnerable.[31] These complications can impede functional recovery, prolong hospital admissions, and are independently associated with an increased risk of death or long-term dependency.[31, 33-37] Earlier trials have suggested that the prevention of these complications may improve outcomes after stroke.[46-48]

The medications tested in PRECIOUS has been used for decades in patients with acute stroke to treat nausea and vomiting (metoclopramide), infections (ceftriaxone), and fever (paracetamol). As outlined in paragraph 5.3, serious side effects of these drugs are uncommon when used according to their marketing authorisations. As outlined above, their use in clinical trials on the prevention of complications in patients with acute stroke was safe and not associated with an increase in serious adverse events (SAEs).[46-48] According to the Dutch NFU guideline, the risk associated with participation in this study is therefore small.

Given the potential benefit of the prevention of complications to the included subjects, future stroke patients, their caregivers, and society on the one hand and the limited risks to trial subjects on the

other hand, the trial's Steering Committee and Ethics Advisory board conclude on a risk-benefit balance strongly in favour of conducting this clinical trial.

Inclusion of patients with incapacity

A vital criterion for valid consent by the patient for inclusion in a clinical trial is the patient's decision-making capacity. The criteria for assessing decision making capacity vary from country to country, but generally include four interrelated capacities: to understand relevant information, to appreciate the current situation and consequences of decisions, to use sufficient reasoning to make decisions, and to communicate a choice.[54, 55] PRECIOUS will include elderly patients with moderately severe to severe stroke (NIHSS ≥ 6) because these patients are at the highest risk of complications and of a poor outcome.[13, 31, 56] The large majority of patients with moderately severe to severe stroke have a diminished decision-making capacity because of a reduced level of consciousness, aphasia, or another cognitive disorder. Patients with an NIHSS ≥ 6 but with a maintained capacity to provide informed consent will have considerably smaller infarcts and will have a stroke severity at the less severe end of the spectrum. Results of trials obtained in patients with the capacity to provide consent can therefore not be extrapolated to patients who cannot give consent. For these reasons, the trial Steering Committee and Ethical Advisory Board feel that it is ethically appropriate to include incapacitated patients in this trial if the informed consent of their legally designated representative has been obtained. Depending on local and national regulations, an incapacitated patient may also be included after approval by an independent physician, followed at a later point in time by informed consent from the patient or his/her legal representative.

Incapacitated subjects will be provided study information in a way that is adequate in view of their capacity to understand it. The explicit wish of an incapacitated subject who is capable of forming an opinion to refuse participation in, or to withdraw from, PRECIOUS at any time, will be respected by the investigator. No incentives or financial inducements will be given to the patients or their legally designated representatives.

This policy is in line with 'regulation No 536/2014 of the European Parliament and of the Council of 16 April 2014 on clinical trials on medicinal products for human use' because data of comparable validity cannot be obtained in clinical trials on persons able to give informed consent, or by other research methods; PRECIOUS relates directly to the medical condition from which the patient suffers; and there are scientific grounds for expecting that participation in the clinical trial may produce a direct benefit to the incapacitated subject outweighing the risks and burdens involved if the patient will receive active trial treatment. On participants not receiving active trial treatment, PRECIOUS will impose minimal burden.

Incapacitated patients will be provided with trial information and will be asked to give informed consent as soon as they will have regained their decision-making capacity.

Rules and regulations for the inclusion of incapacitated patients in clinical trials differ between the participating countries. In each country, the specific rules and regulations will be followed.

What PRECIOUS will add

As outlined above, the prevention of complications with the treatments proposed in PRECIOUS was safe in previous trials and not associated with an increased risk of SAEs.[46-48] However, all available studies with the exception of PASS and STROKE-INF were underpowered to detect clinically relevant effects on long-term outcomes. The lack of a benefit observed in PASS may be explained by the inclusion of many patients with very mild strokes and the associated low incidence of pneumonia. As mentioned above, strengths of PRECIOUS are the short time window for start of treatment of 24 hours and its multifactorial design, with combination treatment possibly leading to a larger reduction in the risk of complications than the individual treatments alone. The above-mentioned trials therefore strongly suggest that the pharmacological prevention of complications is likely to have a larger benefit than current clinical practice of starting treatment when complications have already developed. PRECIOUS will be able to detect a 5% absolute reduction in the risk of death or long-term dependency because of its sample size of 3800 patients and because the population of elderly patients with moderately severe to severe stroke included in PRECIOUS has a considerably higher baseline risk of both complications and a poor outcome than the more general populations assessed in earlier studies.[31, 57] The results of PRECIOUS may be implemented rapidly throughout Europe, because the trial will provide high-quality evidence on the effects of the prevention of complications after stroke that will be broadly generalisable, and because the drugs tested are cheap, safe, and generally available throughout Europe.

2. OBJECTIVES & OUTCOMES

2.1. STUDY OBJECTIVES

Primary objective

To assess whether prevention of aspiration, infections, or fever with metoclopramide, ceftriaxone, paracetamol, or any combination of these in the first four days after stroke onset improves functional outcome at 90 days in elderly patients with acute stroke.

Secondary objectives

- To assess the effect of prevention of aspiration, infections and fever in the first days after stroke onset with metoclopramide, ceftriaxone, paracetamol, or any combination of these, on the following outcomes at 90 days.
  - Death
  - Death or dependency
  - Quality of life
  - Cognition
  - Costs
- To detect specific populations classified by age, sex, stroke severity, body temperature, co-morbidities, and geographic region, in which the proposed treatments are particularly effective or not effective.
- To assess the effects of prophylactic treatment with ceftriaxone on antimicrobial resistance and the occurrence of infections with Clostridium difficile.

2.2. STUDY OUTCOMES

2.2.1. Primary outcome

The primary outcome measure is the score on the modified Rankin Scale (mRS)[58] at 90 days (± 14 days). The mRS is the preferred disability parameter for clinical trials in stroke.[59] The mRS is an ordinal hierarchical scale incorporating six categories from 0 up to and including 5, and describes the range of disability encountered post stroke. 'Death' is assigned a score of 6.

2.2.2. Secondary outcomes

At 7 days (± 1 day) or at discharge, if earlier:

- Infections in the first 7 days (± 1 day; frequency, type, and *C. difficile* infections). Infections will be categorised as diagnosed by the clinician, and as judged by an independent adjudication committee (masked to treatment allocation);
- 3rd generation cephalosporin resistance in the first 7 days (± 1 day), detected as part of routine clinical practice;
- Antimicrobial use during the complete hospital admission for stroke, converted to units of defined daily doses according to the classification of the WHO Anatomical Therapeutic Chemical Classification System with Defined Daily Doses Index (http://www.whocc.no/);
- SAEs in the first 7 days.
- In a subgroup of patients: presence of Extended-Spectrum Beta-Lactamase (ESBL)-producing bacteria as detected by PCR in a rectal swab at day 7 (± 1 day, or at discharge, if earlier).

At 90 days (± 14 days):

- Death;
- Unfavourable functional outcome, defined as mRS 3 to 6;
- Disability assessed with the score on the Barthel Index[60] (BI);
- Cognition assessed with the Montreal Cognitive Assessment[61] (MoCA);
- Quality of life assessed with the EuroQol 5D-5L (EQ-5D-5L);
- Home time: the number of nights among the first 90 since stroke onset that are spent in the patient's own home or a relative's home. Resource use will be censored at 90 days. Where final follow-up occurs earlier, the last known placement will be extrapolated to 90 days;[62]
- Patient location over first 90 days (± 14 days): hospital; rehabilitation service; chronic nursing facility; home.

3. STUDY DESIGN

This is an international, multi-centre, multi-factorial, randomised, controlled, open-label clinical trial with blinded outcome assessment (PROBE) of ceftriaxone, paracetamol, and metoclopramide, or any combination of these, in 3800 elderly patients with acute ischaemic stroke or intracerebral haemorrhage. Patients will be recruited in about 80 hospitals in about 9 European countries over a period of about seven years. To increase the generalisability of the findings, these countries are proportionally distributed over Europe, and include Estonia, Germany, Greece, Hungary, Italy, the Netherlands, Norway, Poland, and the United Kingdom. For the same reason, the trial will recruit patients both in academic and regional hospitals. The trial will be performed according to ICH-GCP principles, the Declaration of Helsinki as most recently amended in 2013, and national and international regulatory requirements.

Before the opening of their site for patient recruitment, each local investigator will receive training in the relevant trial-specific procedures. Investigators will be trained in outcome assessment using a validated web-based training programme hosted at Glasgow University.

4. STUDY POPULATION

4.1. SUBJECT SELECTION

The study population will consist of patients aged 66 years or older who are hospitalised with a clinical diagnosis of acute ischaemic stroke or intracerebral haemorrhage.

4.2. INCLUSION CRITERIA

In order to be eligible to participate in this study, a subject must meet all of the following criteria:
1. A clinical diagnosis of acute ischaemic stroke or intracerebral haemorrhage, confirmed with CT or MRI scan. A normal CT scan is considered compatible with ischaemic stroke;
2. A score on the National Institutes of Health Stroke Scale[63] (NIHSS) ≥ 6, indicating moderately severe to severe stroke;
3. Age 66 years or older;
4. The possibility to start treatment within 24 hours of symptom onset;
5. Written informed consent.

4.3. EXCLUSION CRITERIA

A subject who meets any of the following criteria will be excluded from participation in this study:
1. Active infection requiring antibiotic treatment, as judged by the treating physician;
2. Pre-stroke score on the mRS ≥4
3. Death appearing imminent at the time of assessment.

In addition, patients will be excluded from participation in the trial treatment strata for any of the following reasons, which are standard for use of the interventions in clinical routine:
<u>For the metoclopramide stratum:</u>
1. Hypersensitivity to metoclopramide or to any of the excipients;
2. Gastrointestinal haemorrhage, mechanical obstruction or gastro-intestinal perforation for which the stimulation of gastrointestinal motility constitutes a risk;
3. Confirmed or suspected pheochromocytoma;
4. History of neuroleptic or metoclopramide-induced tardive dyskinesia;
5. Epilepsy;
6. Parkinson's disease;
7. Use of levodopa or dopaminergic agonists;
8. Known history of methaemoglobinaemia with metoclopramide or of NADH cytochrome-b5 deficiency.
9. Clinical indication for the use of metoclopramide. Incidental use metoclopramide before screening is not an exclusion criterion.

<u>For the ceftriaxone stratum:</u>
1. Known hypersensitivity to beta-lactam antibiotics;
2. Clinical indication for antibiotic treatment. Use of an antibiotic before screening is not an exclusion criterion.

<u>For the paracetamol stratum:</u>
1. Known hypersensitivity to paracetamol or any of the excipients;
2. Known severe hepatic insufficiency;
3. Chronic alcoholism;

4. Clinical indication for the use of paracetamol. Incidental use of paracetamol before screening is not an exclusion criterion.;

4.4. SAMPLE SIZE

PRECIOUS is powered to detect a statistically significant shift in the difference in the proportion of patients with mRS 0 to 2 at 90 days, assuming an effect that leads to a 5% absolute increase (from 36 to 41%)[64] in the cumulative proportion of patients with mRS 0 to 2 in any intervention group, compared with controls. The chance of 36% of survival without dependency is based on findings in IST-3, a trial including 3035 patients with acute ischaemic stroke, of whom 89% were 61 years of age or older; 80% had a baseline NIHSS of 6 or greater; and 94% was included in Europe. The effect size of 5% is based on previous smaller studies and/or meta-analyses thereof, performed in more general stroke populations.[45-48] In addition, we presume that 5% absolute risk reduction will be sufficient to change clinical practice across Europe. We assume that each of the three active interventions will be censored in 20% of the patients and we conservatively assume that the use of covariate adjustment will increase statistical efficiency by 25%.[65, 66] Based on a binary analysis, a total study size of 3719 evaluable patients then allows for a power of 90% to detect a significant difference in the scores on the mRS for each of the intervention groups compared with the control group at a 5% significance level. To allow for loss to follow-up, this is rounded to 3800 patients.

5. TREATMENT OF SUBJECTS

5.1. STUDY PHASES

5.1.1. Screening Phase

Written, signed, and dated informed consent by the patient or a patient representative or approval by an independent physician, as agreed upon with the local Ethics Committee and in accordance with national requirements must be obtained by an investigator prior to the performance of any study-related procedure. The investigator will provide the patient or the patient representative with information of the study and explain the nature of the study.

Prior to enrolment, all patients will receive usual care as recommended by national and/or international guidelines and/or local protocols. This may include thrombolysis and endovascular treatment for acute ischaemic stroke, and treatment of hypertension for intracerebral haemorrhage. Demographic and clinical parameters may be collected as part of usual care and used to screen patients for participation in the present study, but no study-specific procedures will be performed before written informed consent has been obtained. Because trial treatment should be started within 24 hours of symptom onset, no screening activities will be performed after this time window.

5.1.2. Hospital Phase

The time of randomisation is defined as the start of the Hospital Phase and as the beginning of Day 1. This phase will therefore start within 24 hours of symptom onset, and continue for 7 days (± 1 day) or until discharge, if earlier. This also applies to patients who terminate the trial treatment prematurely.

5.1.3. Follow-up Phase

The Follow-up Phase will start on Day 8 or on the day after discharge, if earlier, and will continue until the final follow-up visit at 90 (± 14) days.

5.2. TREATMENT ALLOCATION

Patients will be randomly allocated in a 2*2*2 factorial design to any combination of open-label oral, rectal, or intravenous metoclopramide (10 mg thrice daily), intravenous ceftriaxone (2000 mg once daily), oral, rectal, or intravenous paracetamol (1000 mg four times daily), or usual care, started within 24 hours after symptom onset and continued for 4 days or until complete recovery or discharge from hospital, if earlier. In patients with moderate to severe renal impairment or with severe hepatic impairment, the dose of metoclopramide is reduced to 5 mg thrice daily, and in patients with end-stage renal disease to 2.5 mg thrice daily. Allocation will be based on proportional minimisation through a web-based allocation service (Figure 1). Investigators will have the opportunity to censor a single randomisation stratum in a specific patient before randomisation, for example in case of an allergy to one of the interventions. Reasons for censoring a treatment will be recorded in the electronic case report form (eCRF). Treatment allocation will be stratified by country and will include the following minimisation factors for balance in baseline characteristics: age (66 – 75 years vs. > 75 years); sex (male vs. female); stroke type (ischaemic stroke vs. intracerebral haemorrhage); stroke severity (NIHSS 6 – 12 vs. > 12); and diabetes mellitus (yes vs. no).

Figure 1. Treatment allocation options in PRECIOUS

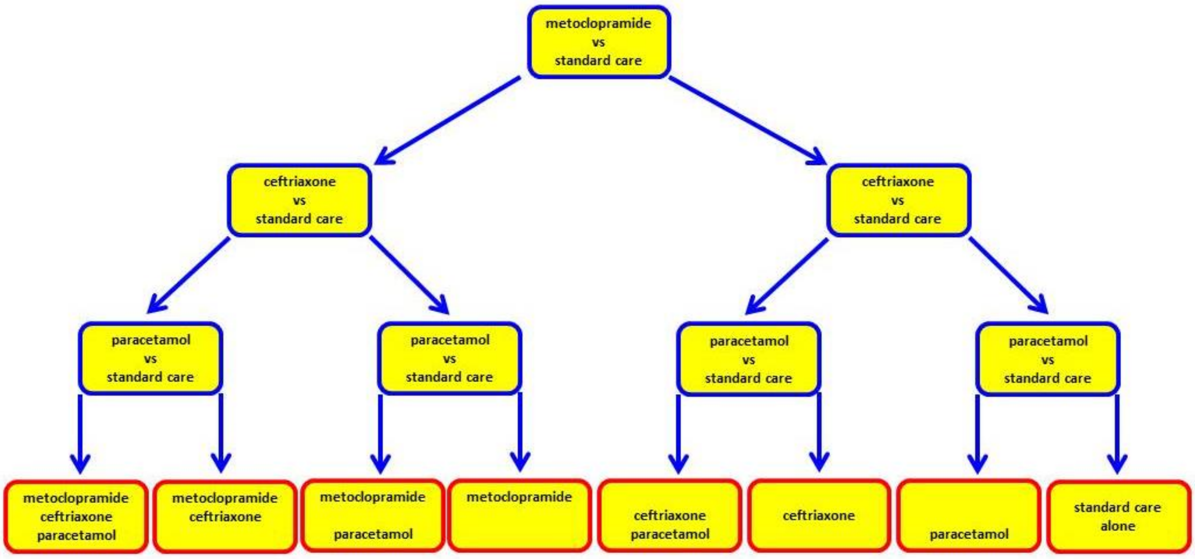

Treatment allocation will be based on proportional minimisation. Investigators will have the opportunity to censor a single randomisation stratum in a specific patient before randomisation.

5.3. DESCRIPTION OF INVESTIGATIONAL MEDICINAL PRODUCTS

Metoclopramide, ceftriaxone, and paracetamol defined by their active substance will be administered as study drugs after randomisation to one of the treatment strata.
The investigational medicinal product (IMP) is defined as the medication administered according to the corresponding treatment stratum. If one of the three active substances (metoclopramide, ceftriaxone, or paracetamol) is administered as part of routine clinical care and not as a treatment according to the protocol, the product is by definition not an IMP.

5.3.1. Metoclopramide

ATC code: A03F A01.

Properties
Metoclopramide is a substituted benzamide. It is used for, among other things, its anti-emetic properties. Besides a central action, metoclopramide has a stimulating effect on the digestive motor system via a peripheral mechanism of action. There is an antidopaminergic effect and an enhancement of the action of acetylcholine. This results in accelerated emptying of the stomach and increased pressure of the lower oesophageal sphincter. Metoclopramide has no influence on gastric secretion. For details we refer to the relevant SmPCs. Note that for this off-patent drug, a wide range of SmPCs is available, with subtle differences. The SmPCs submitted for review by Competent Authorities and Research Ethics Committees should therefore be considered examples. Annual review of the SmPCs will only lead to amendments to the study protocol in case of new clinical findings that are of direct relevance to the study population.

Contraindications
- Hypersensitivity to the active substance or to any of the excipients listed in the SmPC

- Gastrointestinal haemorrhage, mechanical obstruction or gastro-intestinal perforation for which the stimulation of gastrointestinal motility constitutes a risk;
- Confirmed or suspected pheochromocytoma, due to the risk of severe hypertension episodes;
- History of neuroleptic or metoclopramide-induced tardive dyskinesia;
- Epilepsy (increased crises frequency and intensity);
- Parkinson's disease
- Combination with levodopa or dopaminergic agonists;
- Known history of methaemoglobinaemia with metoclopramide or of NADH cytochrome-b5 deficiency;
- For the suppository: recent history of proctitis or rectal bleeding

Undesirable effects
We refer to the SmPCs of metoclopramide and to appendix 7. Common side effects (occurring in one or more of 100 patients) include:
- Diarrhoea
- Asthenia
- Extrapyramidal disorders (particularly in children and young adults and/or when the recommended dose is exceeded; this risk appears lower in elderly patients included in the present trial)
- Depression

Dose
Metoclopramide will be administered as 10 mg tablets, 10 mg suppositories, or 10 mg intravenous infusions in a daily dose of 3 times 10 mg for a period of 4 days or until discharge, if earlier. In patients with end-stage renal disease (creatinine clearance ≤ 15 ml/min), the daily dose should be reduced to 3 times 2.5 mg; in patients with moderate to severe renal impairment (creatinine clearance 15 to 60 ml/min), the dose should be reduced to 3 times 5 mg. In patients with severe hepatic impairment (liver cirrhosis), the dose should be reduced to 3 times 5 mg.

Interaction with other medicinal products

General
Due to the prokinetic effect of metoclopramide, the absorption of certain drugs may be modified.

Anticholinergics and morphine derivatives
Anticholinergics and morphine derivatives may have both a mutual antagonism with metoclopramide on the digestive tract motility.

Central nervous system depressants (morphine derivatives, anxiolytics, sedative H1 antihistamines, sedative antidepressants, barbiturates, clonidine and related)
Sedative effects of central nervous system depressants and metoclopramide are potentiated.

Neuroleptics
Metoclopramide may have an additive effect with other neuroleptics on the occurrence of extrapyramidal disorders.

Serotonergic drugs

The use of metoclopramide with serotonergic drugs such as SSRIs may increase the risk of serotonin syndrome.

Digoxin
Metoclopramide may decrease digoxin bioavailability. Careful monitoring of digoxin plasma concentration is required.
Cyclosporine
Metoclopramide increases cyclosporine bioavailability (Cmax by 46% and exposure by 22%). Careful monitoring of cyclosporine plasma concentration is required. The clinical consequence is uncertain.

Mivacurium and suxamethonium
Metoclopramide injection may prolong the duration of neuromuscular block (through inhibition of plasma cholinesterase).

Strong CYP2D6 inhibitors
Metoclopramide exposure levels are increased when co-administered with strong CYP2D6 inhibitors such as fluoxetine and paroxetine. Although the clinical significance is uncertain, patients should be monitored for adverse reactions.

Preparation and labelling
Study medication is defined by active substance and will be labelled according to national rules and regulations. Metoclopramide will be prescribed, dispensed and used in the same way as in routine clinical practice. Possible interaction with non-study medication will be carefully checked.

Drug accountability
No specific drugs accountability will be needed, as metoclopramide will be prescribed, dispensed and used in the same way as in routine clinical practice. However, the daily dose and the time of the first administration will be recorded in the eCRF, with 100% source data verification.

5.3.2. Ceftriaxone

ATC-Code: J01DD04

Properties
Ceftriaxone is a parenterally administered, bactericidally acting β–Lactam-antibiotic of the pharmacotherapeutic group of cephalosporins. The bactericidal activity of ceftriaxone results from the inhibition of bacterial cell wall synthesis (during the period of growth) caused by an inhibition of Penicillin-binding proteins (PBPs) like transpeptidases. For details we refer to the relevant SmPCs. Note that for this off-patent drug, a wide range of SmPCs is available, with subtle differences. The SmPC submitted for review by Competent Authorities and Research Ethics Committees should therefore be considered an example. Annual review of the SmPC will only lead to amendments to the study protocol in case of new clinical findings that are of direct relevance to the study population.

Contraindications
- Hypersensitivity to ceftriaxone, the active substance, or to other cephalosporins.
- Previous immediate and/or severe hypersensitivity reaction to a penicillin or to any other beta-lactam medicinal products.

Undesirable effects
We refer to the SmPCs of ceftriaxone and to appendix 7. Common side effects (occurring in one or more of 100 patients) include:

- Phlebitis following intravenous administration that can be minimised by slow injection (over 2-4 minutes).
- Pain at the site of injection.
- Allergic reaction (e.g. dermatitis, urticaria, exanthema), pruritus, oedematous swelling of skin and joints.
- Drug fever, shivering.
- Elevated liver enzymes in serum (AST, ALT, alkaline phosphatase).

Dose
Ceftriaxone will be administered by slow intravenous infusion after reconstitution of the solution according to the directions given in the SmPCs in a once-daily dose of 2000 mg for a period of 4 days or until discharge, if earlier.

Preparation and labelling
Study medication is defined by active substance and will be labelled according to national rules and regulations. Ceftriaxone will be prescribed, dispensed and used in the same way as in routine clinical practice. Possible interaction with non-study medication will be carefully checked.

Drug accountability
No specific drugs accountability will be needed, as the ceftriaxone will be prescribed, dispensed and used in the same way as in routine clinical practice. However, the daily dose and the time of the first administration will be recorded in the eCRF, with 100% source data verification.

5.3.3. Paracetamol

ATC code: N02BE01.

Properties
Paracetamol has analgesic and antipyretic actions, but it has no anti-inflammatory properties. The mechanism of action of paracetamol has not been fully clarified. The effect seems to be based on inhibition of the enzyme prostaglandin synthetase, but this does not explain the lack of anti-inflammatory actions.
Distribution of paracetamol throughout the body and thus the location of the inhibition of prostaglandin synthetase may also be of importance. The benefit of paracetamol lies in the fact that some of the adverse effects characteristic of NSAIDs are completely or largely absent. For details we refer to the relevant SmPCs. Note that for this off-patent drug, a wide range of SmPCs is available, with subtle differences. The SmPCs submitted for review by Competent Authorities and Research Ethics Committees should therefore be considered examples. Annual review of the SmPC will only lead to amendments to the study protocol in case of new clinical findings that are of direct relevance to the study population.

Contraindication

- Hypersensitivity to the active substance or to any of the excipients.

Undesirable effects

We refer to the SmPCs of paracetamol and to appendix 7. Few adverse effects occur with therapeutic dosages. The SmPCs list no side effects that occur in one or more of 100 patients.

Dose

Paracetamol will be administered as 500 mg or 1000 mg tablets, 1000 mg suppositories, or 1000 mg intravenous infusions in a daily dose of 4 times 1000 mg for a period of 4 days or until discharge, if earlier. In Italy and Norway, the daily dose of 4000 mg daily is higher than the maximum daily dose of 3000 mg recommended in the SmPCs in these countries, but a daily dose of 4000 mg has been approved by the Italian and Norwegian competent authorities for use in PRECIOUS. Paracetamol will only be given intravenously if oral or rectal administration is impossible, impractical, or refused by the patient.

Preparation and labelling

Study medication is defined by active substance and will be labelled according to national rules and regulations. Paracetamol will be prescribed, dispensed and used in the same way as in routine clinical practice. Possible interaction with non-study medication will be carefully checked.

Drug accountability

No specific drug accountability will be needed, as the IMP will be prescribed, dispensed and used in the same way as in routine clinical practice. However, the daily dose and the time of first administration will be recorded in the eCRF, with 100% source data verification.

5.4. PREGNANCY AND CONTRACEPTION

Not applicable: the lowest age for inclusion is 66 years.

5.5. CONCOMITANT MEDICATIONS AND TREATMENTS

Patients in each study group will be treated according to national and international guidelines and local protocols. If fever or other signs of infection occur during treatment with trial medication, the source of fever will be ascertained and treated. In all treatment groups, the administration of any antiemetic, antibiotic, or antipyretic drug during the first 7 days (or until discharge, if earlier) will be registered in the eCRF, including type, timing (day), and daily dose. The total dose of the study medication should never exceed the maximum recommended daily dose for that drug.

6. STUDY PROCEDURES

6.1. BASELINE ASSESSMENT

The following baseline characteristics will be assessed during the screening phase as part of usual clinical care:

- Demographics: age; sex; ethnicity
- Comorbidities/medical history: atrial fibrillation; diabetes mellitus; hypertension; pre-stroke mRS
- Concurrent drugs: use of any antipyretic, antibiotic, or antiemetic drug in the 3 days before randomisation. Aspirin in any formulation and in a daily dose of up to 300 mg is NOT considered an antipyretic drug.
- Way of food intake on the day before the stroke (see 6.9);
- Treatment restrictions (see 6.10)
- Dates and times: stroke onset, hospital admission
- Vital signs: blood pressure; pulse; body temperature;
- Neurological examination: NIHSS (see 6.3); expected location of the lesion (left or right hemisphere; posterior fossa);
- Laboratory examinations (see 6.5);
- Results of chest X-ray and urine analysis if performed as part of routine clinical practice;
- Imaging results: stroke type: ischaemic stroke or intracerebral haemorrhage (see 6.6).
- Previous treatment: intravenous thrombolysis with alteplase; intra-arterial treatment.

6.2. NATIONAL INSTITUTES OF HEALTH STROKE SCALE (NIHSS)

The NIHSS is an ordinal hierarchical scale to evaluate the severity of stroke by assessing a patient's performance (Appendix 2).[63] Scores range from 0 to 42, with higher scores indicating a more severe deficit. A web-based training programme using video clips will be available via the PRECIOUS website. At each site, at least one Investigator must be certified in the assessment of the NIHSS.

6.3. MODIFIED RANKIN SCALE (MRS)

The mRS is the preferred functional outcome measure in clinical stroke studies.[58] The mRS is an ordinal hierarchical scale that describes the range of disabilities encountered post stroke, incorporating six categories from 0 (complete recovery) up to and including 5 (severe disability). 'Death' is assigned a score of 6 (Appendix 3). Despite its apparent simplicity, assessment of the score on the mRS has been associated with considerable inter-observer variability, especially in multicentre studies. This inter-observer variability may affect trial power and treatment effect size. In addition, knowledge of treatment allocation can influence outcome assessment, and unblinded trials like PRECIOUS are therefore subject to a major potential for detection bias. In PRECIOUS, these two major issues are minimised through 1) online training and certification of outcome assessors with a link via the PRECIOUS website; and 2) central outcome adjudication by three independent and blinded raters based on digital video recordings of the 90-day outcome interviews. This central adjudication offers many benefits:

1. blinding is assured;
2. standardisation is possible across multiple regions and cultures;
3. statistical power is enhanced through the use of three repeated assessments;
4. the estimate of treatment effect size is restored (since statistical noise leads to underestimation);

5. it provides independent validation of the information that is collected, thereby minimising the risk of fraud;
6. site staff perform to a higher standard when aware that there will be review or audit of their activity.

The mRS assessments will be performed at 7 (± 1) and 90 (± 14) days in a standardised fashion according to each centre's normal practice. The assessment at 90 days will be recorded using a digital video camera. During this recording, no reference to the treatment allocation will be made. The videos will be uploaded to a secure server at UGLA where they will be checked, stored and distributed for independent scoring by three certified expert raters from the same country as the patient, with committee discussion where appropriate. Where relevant for participants with impaired communication or insight, a care giver can be included in the recorded mRS assessment alongside the participant to supplement or confirm responses. Where video recording of the patient is not possible, a video recording of an interview with a nurse or caregiver will be uploaded. The reason for not being able to record the interview with the patient should be noted in the eCRF. In case of death before day 90, the patient will be assigned an mRS score of 6.

6.4. BLOOD PRESSURE, PULSE, AND BODY TEMPERATURE

Blood pressure, pulse, and body temperature will be assessed in the Screening Phase. Where assessed as part of routine clinical practice during the hospital phase, blood pressure, pulse, and body temperature will be recorded at 12-hour (± 3 hours) intervals. Both rectal and tympanic thermometry are allowed, but the method of thermometry will be noted in the eCRF.

6.5. LABORATORY TESTS

If assessed at baseline as part of routine clinical practice, results from the following laboratory tests will be collected: serum glucose; glomerular filtration rate; C-reactive protein (CRP); alkaline phosphatase (ALP); gamma-glutamyl transferase (GGT); alanine aminotransferase (ALT); and aspartate aminotransferase (AST); leucocyte count and differential.

6.6. IMAGING

Brain CT or MRI will be performed as part of routine clinical care before randomisation. Results (visible ischaemic damage; intracerebral haemorrhage) will noted in the eCRF. If a chest X-ray has been performed before randomisation as part of routine clinical care results will also be noted in the eCRF. No additional imaging will be performed as part of PRECIOUS, and images will not be sent to the central trial office.

6.7. MEDICATION

The time of the first dose of each study medication will be recorded. During the first 7 days (± 1 day, or up to discharge, if earlier), the total daily dose of the study medication will be recorded, as well as that of any other antipyretic, antibiotic, or antiemetic drug. Aspirin in any formulation and in a daily dose of up to 300 mg is NOT considered an antipyretic drug.

6.8. INFECTIONS AND RESISTANCE

Infections, use of antibiotics, and selection of bacteria with increased antimicrobial resistance during the first 7 days (or until discharge, if earlier) will be recorded in the eCRF by the local investigators. Antimicrobial use during hospital stay converted to units of defined daily doses according to the

classification of the WHO Anatomical Therapeutic Chemical Classification System with Defined Daily Doses Index (http://www.whocc.no/).
Infections will be categorised as diagnosed by the clinician, and as judged by an independent adjudication committee (masked to treatment allocation) according to modified Centers for Disease Control and Prevention criteria.[67] The scoring algorithms for infections that will be used by this committee have been described previously,[68] and are in line with recommendations of the Pneumonia in Stroke Consensus Group.[69] *Clostridium difficile* infection will be defined as diarrhoea in combination with a positive *C. difficile* toxin test.
To detect selection of bacteria with 3rd generation cephalosporin resistance caused by increased antibiotic pressure, a nested case-control substudy will be performed in 1000 patients in 30 centres in different participating countries. The presence of ESBL-producing bacteria will be assessed with PCR. With this purpose two rectal swabs will be collected in each patient, after specific informed consent, on admission and at day 7 (± 1 day, or at discharge, if earlier) and sent to the central laboratory at the AMC in Amsterdam, the Netherlands. We aim to be able to detect a difference of at least 5% in 3rd generation cephalosporin resistance between the two treatment groups (ceftriaxone vs. control) at day 7. The sample size is based on resistance rates in E. coli, because of the high carriage rate of *Escherichia coli* in the population in general. Nevertheless, resistance in other Enterobacteriaceae (such as *Klebsiella pneumoniae*) and enterococci will also be investigated. A two group Chi^2 test with a 0.05 two-sided significance level will have 80% power to detect this change (odds ratio of 2.2) when the sample size in each group is 278. However, we cannot assume a 100% detection of E. coli in all samples; also mortality during the first week has to be taken into account. This substudy will therefore be performed in 500 patients in each group, and in 1000 patients in total.

6.9. FEEDING

The method of feeding on the day before the stroke and at noon of the relevant day during the Hospital Phase will be recorded and classified as 1. normal food; 2. oral, soft or fluids only; 3. nasogastric tube; 4. percutaneous endoscopic gastrostomy (PEG); 5. intravenous only; 6. none.

6.10. TREATMENT RESTRICTIONS

The presence of any treatment restriction will be recorded at baseline and during the Hospital Phase, and will be classified as 1. Do not resuscitate; 2. Do not intubate and ventilate; 3. Withholding other treatments that may prolong life; 4. Withholding food; 5. Withholding fluids; and 6. Palliation with morphine or a benzodiazepine. Any combination of these strategies is possible.

6.11. PATIENT LOCATION

The location of the patient at noon of the relevant day during the Hospital Phase and follow-up phase will be recorded and classified as: hospital; rehabilitation service; chronic nursing facility; home (own or relative's). 'Home time,' defined as the number of nights among the first 90 since stroke onset that are spent in the patient's own home or a relative's home is a secondary outcome, for which the resource use will be censored at 90 days. Where final follow-up occurs earlier, the last known placement will be extrapolated to 90 days.[62]

6.12. INDIRECT SOCIETAL COSTS

As an estimate of indirect societal costs of stroke, the number of days relatives and other caregivers have been absent from work in the first 90 days after stroke will be recorded.

6.13. MONTREAL COGNITIVE ASSESSMENT (MOCA)

The Montreal Cognitive Assessment (MoCA) is a short cognitive test for use in patients with stroke and other neurological conditions.[61] It has a high sensitivity for cognitive impairment as defined using more extensive neuropsychological test batteries.[70, 71] Test scores range from 0 to 30, with higher scores indicating better cognitive performance. The test is currently available in 46 languages and dialects [www.mocatest.org]. An English example is provided in Appendix 4. The MoCA will be administered at the end-of-trial visit at 90 days (± 14 days).

It is conceivable that the MoCA cannot be assessed reliably in some patients, for example because of severe aphasia or severe comorbidities. This will not be considered a protocol deviation or violation if the reason for not assessing the test is provided in the eCRF.

6.14. BARTHEL INDEX (BI)

The BI is an ordinal scale used to measure performance in 10 activities of daily living (ADL; Appendix 5).[60, 72, 73] Test scores range from 0 to 100, with higher scores indicating better performance in these activities. The BI will be assessed at the end-of-trial visit at 90 days (± 14 days).

6.15. EUROQOL (EQ-5D-5L)

The EuroQoL 5-dimensions 5-level (EQ-5D-5L) questionnaire is a standardised measure of health outcome that has been used extensively in patients with stroke (Appendix 6).[21, 25, 74] It is easy to understand and takes a few minutes to complete. The questionnaire is primarily designed for self-completion by patients, but if the patient is impaired in writing, the EQ-5D-5L may be completed by a representative of the patient or by the trial nurse, both as instructed by the patient. If the patient will not be able to complete the questionnaire because of aphasia or cognitive impairment, the patient's representative will do this instead of the patient. The EQ-5D-5L will be assessed at the end-of-trial visit at 90 days (± 14 days).

6.16. SAFETY

See chapter 9.

7. STATISTICAL METHODS & CONSIDERATIONS

7.1. STATISTICAL ANALYSIS PLAN

The analysis and reporting of the trial will be in accordance with CONSORT guidelines. Before follow-up will have been completed, a statistical analysis plan (SAP) will be developed that will specify: (i) Hypotheses to be tested; (ii) Treatment effects to be estimated in order to satisfy the primary and secondary objectives of the PRECIOUS trial; (iii) Technical description of the statistical methodology and procedures for performing the statistical analysis of outcome measures and AE data; (iv) Primary, secondary, and sensitivity analyses; and (v) Subgroup analyses. The SAP will be signed off by the trial Steering Committee and then submitted for publication prior to data lock and final analysis.

Three processes will be performed to test and validate the analysis system once about 100 patients have been recruited and followed to 90 days: (i) Data transfer from trial database to statistician; (ii) Statistical analysis to ensure column totals match; (iii) Check analyses between master and mirror code. This does not constitute an interim analysis as the sample size is small compared with the overall sample size, and risk of random error so high that no inferences of the intervention effect would be possible. This pilot is performed not as an analysis per se but to ensure the feasibility, efficiency and adequate responsiveness of the developed statistical systems. The pilot will be performed blinded to treatment allocation.

The final statistical analysis will be performed once recruitment has ceased, final follow-up has been completed, final data have been checked and any errors corrected, and the database has been locked. To meet the primary objective of the PRECIOUS trial, statistical analysis will assess whether any of the three interventions improves functional outcome at 90 days in patients with acute stroke. To meet the secondary objective of the PRECIOUS trial, statistical analysis will assess whether one or more particular patient groups are more or less likely to benefit from each of the interventions. All secondary, per-protocol analyses and sensitivity analyses will be considered as hypothesis-generating and will not be the basis for any recommendations on whether to use any of the interventions in patients with stroke. The analyses will be carried out according to the pre-defined SAP.

7.2. SAMPLE SIZE

See paragraph 4.4.

7.3. ANALYSIS OF PRIMARY ENDPOINT

The primary effect estimate will be the difference in the mean mRS scores obtained through centralised adjudications and assessed using multiple regression, and will be expressed as a mean difference with 95% confidence interval. The statistical analyses will be performed according to the intention-to-treat principle and adjusted for the minimisation factors mentioned in paragraph 5.2, other relevant baseline characteristics, and treatment allocation for the other two strata of the trial. Three separate primary analyses will be performed, looking at the main effects of each of the three interventions compared with their respective controls. Although the study is not powered to detect interactions between the three interventions, such interactions will be investigated in secondary analyses. Two sensitivity analyses will be performed in which all patients who are lost to follow-up will be classified as having the worst possible outcome (death) or the best possible outcome (mRS = 0), respectively.

7.4. ANALYSIS OF SECONDARY ENDPOINTS

Binary logistic regression will be used for binary outcomes, including death, unfavourable outcome, and SAEs. Ordinal logistic regression will be used for ordered categorical data and multiple regression will for continuous outcomes. Wilcoxon rank sum test will be used for continuous outcome measures which are not normally distributed.

7.5. SUBGROUP ANALYSES

Comparison of the effect of the three intervention groups vs. their respective controls on the primary outcome will be performed in the following pre-specified subgroups (assuming sufficient numbers in each subgroup) with assessment of interaction between treatment and the minimisation factors

- Age (≤75, > 75 years);
- Sex (male, female);
- Stroke type (ischaemic stroke, intracerebral haemorrhage);
- Stroke severity (NIHSS 6 – 12, ≥13);
- Diabetes mellitus (yes, no);

and assessment of interaction between treatment and the other baseline factors

- Presence of atrial fibrillation (yes, no);
- Pre-stroke mRS score (0-2, ≥3);
- Treatment with alteplase (yes, no);
- Time to treatment (<6, ≥6 hours, <12 hours, ≥12 hours);
- Treatment allocation for the other two trial strata (paracetamol – active, control; ceftriaxone – active, control; metoclopramide – active, control).

7.6. INTERIM ANALYSES

An independent Data and Safety Monitoring Board (DSMB) will be established to oversee the safety of patients and the efficacy of the interventions in the trial. The DSMB will work in accordance with a dedicated charter and will follow processes recommended by the DAMOCLES statement. With respect to safety, the DSMB will conduct unblinded interim analyses after 600, 1200, 1800, 2400, and 3000 patients have completed follow-up. With respect to efficacy, the DSMB will conduct unblinded interim analyses after 2400 patients had their final follow-up. DSMB members will receive listings of all SAE reports as well as unblinded aggregate summaries of data by treatment groups for review in closed meetings. Feedback, blind to treatment, will be provided in open meetings and in written conclusions to the sponsor and the PRECIOUS chief investigator.

7.7. PROCEDURES FOR MISSING, UNUSED, AND SPURIOUS DATA

Imputation of missing data will not be performed. The primary analysis will be performed on all randomised patients with a valid mRS score at 90 days.

7.8. DEFINITIONS OF POPULATIONS ANALYSED

The following population definitions will be used:

- Intention-to-treat - primary safety analysis: All randomised participants with a vital status recorded at 90 days.
- Intention-to-treat - primary efficacy analysis: All randomised participants who received any study medication and with a valid mRS score recorded at 90 days.
- Per-protocol: All participants in the intention-to-treat population who are deemed to have no major protocol violations that could interfere with the objectives of the study.

All efficacy analyses will be performed on the intention-to-treat population; the robustness of the primary and key secondary analyses will be assessed in the per-protocol population. Safety analyses will be performed on the safety population.

8. DATA COLLECTION, HANDLING AND RECORD RETENTION

8.1. RECORDING OF DATA – SOURCE DOCUMENTATION AND ELECTRONIC CASE REPORT FORMS

Clinical data will be captured using an eCRF, developed with the electronic data capture system Research Online (RO). RO is a fully web-based system that meets all ICH-GCP requirements for electronic data entry with respect to safeguarding data integrity and data security. Required data for this study are to be obtained from the subject's medical notes/source documents where the information was first recorded and then entered into the eCRF. Data from the eCRF will be encoded and stored in a study database. Only authorised and trained site staff will be allowed to enter data into the eCRF and make changes to eCRF data.

All eCRF data should be verifiable to a source at the investigational site or accessible by the site staff.

Data points that are not considered part of the eCRF (e.g. derived data points and administrative data points) will be automatically calculated or entered by authorised staff of the Sponsor or its designee.

All changes made to the eCRF data will be captured via an electronic audit trail, indicating at least date and time of change, the reason for changing the data, the individual that made the change and the old and new data value.

Source documents may include but are not limited to the following original documents, data, and records:

- Hospital records
- Medical histories and narrative statements relating to the subject's progress
- Clinical and office charts
- Operative reports
- Laboratory notes/reports
- Memoranda and telephone notes/records
- ECG recording
- Pharmacy dispensing records
- Recorded data from automated instruments
- Copies of transcriptions certified after verification as being accurate copies

On request the Investigator shall provide the Sponsor access to any required background data from the study documentation or clinical records. This is particularly important when errors in data transcription are suspected. In case of special problems and/or regulatory queries or requests for audit or inspections, it is also necessary to have access to the complete study records, provided that subject confidentiality is protected.

8.2. DATA QUALITY ASSURANCE

The Investigator(s) will be responsible for ensuring eCRF data completeness and accuracy. During data entry RO detects missing data, out-of-range values and data inconsistencies based on implemented validation checks and will then show an error message. The error must be solved immediately at the time of entry or be marked as query in order to be solved later. Show and jump rules will ensure that only applicable information will appear during data entry. The eCRFs will be reviewed by a monitor from the Sponsor or its designee for completeness and accuracy. Source document verification will be performed. The eCRF data will also be reviewed internally by the Sponsor's Data Management, Medical and Scientific staff or their designee and, if necessary, the investigational sites will be queried for corrections and/or clarifications. Once data are concluded to be complete and accurate, the eCRF data will be locked, meaning that the data will become read-only. The Investigator(s) are required to

approve the eCRF data of their site through provisioning of an (electronic) signature before the data is used for final analysis. The Sponsor will ensure that eCRF data is accessible and verifiable by the investigational site and install adequate back-up and security measures to prevent loss of data or unauthorised access to the data.

Copies of pertinent records in connection with the study, including eCRFs and queries, as well as subject charts, laboratory data, etc., will be maintained at the investigational site (see Section 8.4, Record Retention).

8.3. DATA MANAGEMENT

The format and content of the eCRFs will be approved by the Sponsor or its designee prior to the start of the study. The Sponsor or its designee will be responsible for database creation, and coordinating the transfer of data from other data systems (e.g. central laboratory database) into the clinical database.

Prior to finalizing and locking the database, all decisions concerning the inclusion or exclusion of data for each subject in the analyses will be determined by appropriate clinical and statistical personnel. Any exclusion of subject data will be documented as appropriate.

8.4. RECORD RETENTION

In compliance with ICH/GCP guidelines, Investigator(s) will maintain all eCRFs and all source documents supporting data collected from each subject, as well as all study documents as specified in ICH/GCP Section 8, Essential Documents for the conduct of a Study, and all study documents as specified by the applicable regulatory requirement(s). Subject records or other source data must be kept for the maximum period of time mandated by the hospital, institution, or private practice, but for at least 15 years. If off-site archiving is used, all records should be retrieved and made available for review at the time of an audit or regulatory authority inspection.

Video recordings of patients and caregivers represent especially sensitive information and will be stored at the site only until one year after successful upload for central scoring has been confirmed, or such other duration as required by local regulations and IEC approval. For the same reason, video recordings and any related audio file will be stored in the secure central database at the University of Glasgow until only 2 years after database lock. Scores and related comments recorded by the independent raters will be regarded as the source data for mRS and will be retained for at least 15 years.

If it becomes necessary for the Sponsor or the appropriate regulatory authority to review any documentation relating to this study, the Investigator must permit access to such reports.

8.5. MONITORING THE STUDY

PRECIOUS will be monitored by the European Clinical Research Infrastructure Network - European Research Infrastructure Consortium (ECRIN-ERIC) and its partners according to ICH-GCP guidelines and all relevant national and international regulations. Before initiation of the trial, the monitors will receive training from the sponsor UMC about stroke and stroke-associated comorbidities and complications, the study protocol, and the eCRF. This training will also include training from ECRIN-ERIC for the site initiation visit (SIV) procedures and monitoring forms to be used during the study conduct. Trained monitors will perform monitoring activities according to the Monitoring Manual and supervise the maintenance of the Investigator Site File (ISF).

Monitoring of the trials will be done according to the criteria laid down in the PRECIOUS Monitoring Manual, which will be developed by ECRIN in collaboration with UMC and CTC-North. This will include a detailed monitoring plan. All monitoring activities except the initiation visit will be on-site. All sites

will be initiated by telephone interview to ensure that all national, international and study-related requirements are met. Each of the active sites (i.e., open and enrolled at least one patient in the previous year) will be visited at least once a year during the course of the trial. On-site data monitoring will include the verification of data with source documents, considering critical aspects of the trial such as: the informed consent, inclusion and exclusion criteria, primary and secondary endpoints, and (serious) AEs. A monitoring report will be written at the end of each monitoring visit. The last monitoring visit will also be the close-out visit. In addition, continuous remote monitoring with telephone and web-based monitoring visits will be performed in order to assure resolution of all queries.

8.6. AUDITS AND INSPECTIONS

An audit may be performed at any time during or after completion of the study by the Sponsor or their designee. All study-related documentation must be made available to the designated auditor.

In addition, a representative of a regulatory authority may choose to inspect a study site at any time prior to, during, or after completion of the study. A Sponsor or designee representative will be available to assist in the preparation for such an inspection. All pertinent study data should be made available to the regulatory authority for verification, audit, or inspection purposes.

It is important that the Investigator and relevant site staff are available during the audits or inspections and that sufficient time is devoted to the process.

If the Investigator is contacted by any regulatory authority regarding an inspection, the Investigator should contact the Sponsor or their designee immediately.

8.7. CONFIDENTIALITY OF STUDY DOCUMENTS AND SUBJECT RECORDS

The investigator must assure that subjects' anonymity will be maintained and that their identities are protected from unauthorised parties. On eCRFs, other documents and specimens submitted to the Sponsor, including rectal swabs, subjects should not be identified by their names, but by a patient study number. No initials will be used in the eCRF. The investigator will keep a patient identification code list showing codes and names. The investigator will maintain documents not for submission to Sponsor, e.g. subjects' written informed consent forms, in strict confidence.

Video recordings will show the patient's face but will not identify the participant by name. These videos will be checked during adjudication soon after upload to the central adjudication of Rankin scores (CARS) database, to confirm that anonymity was preserved and if necessary may be edited to remove identifying material. The original video recording will be deleted as soon as it is secure to do so, within one year after successful upload has been confirmed; and the centrally held recording will also be deleted within 2 years after database lock. Until then, only qualified certified raters will have access to the video at the time of and for the purpose of adjudication, and such access will be logged.

Retention of video recordings will be fully documented and incorporate a full audit trail. Records will be compliant to relevant GCP guidelines and will be developed and validated in accordance with Computerised Systems for Clinical Research guidelines and 21 CRF Part 11 – Electronic Records and Electronic Signatures. Dates and times will be recorded at both local and central database levels and will be compliant with ISO 8601: 1988 (E) (Data elements and interchange formats – Information interchange – Representation of dates and times). The web pages will only be accessible using secure socket layer (SSL) communication which will utilize a validation certificate created for a particular server within a specific domain. This will enable authentication from the server to the user's browser and will encrypt all traffic between their local computer and the authenticated host server. The web

server will be secured by VeriSign, the BT Trust Services Global Server Certificate program and will be firewall-protected.

9. SAFETY AND AES

In accordance with Art 107(1) of Directive 2001/83/EC, it is the responsibility of the Sponsor and Investigator to report all safety information required to the Regulatory Authorities (RA) and Independent Ethics Committee (IEC) as specified in this Directive for studies.

9.1. SAFETY DEFINITION

9.1.1. Adverse Event (AE)

An Adverse Event (AE) is any untoward medical occurrence in a subject enrolled in this study and which does not necessarily have a causal relationship with this treatment. An AE is therefore any unfavourable and unintended sign (including an abnormal laboratory finding), symptom or disease, whether or not related to the IMP or protocol-related procedures.
An unexpected AE is one of a type not identified in nature, severity, or frequency in the current respective SmPC or of greater severity or frequency than expected based on the information in the respective SmPC.

9.1.2. Serious Adverse Event (SAE) and Serious Adverse Reaction (SAR)

An SAE is defined as any AE occurring at any dose that results in any of the following outcomes:

- death
- a life-threatening adverse experience
- in subject hospitalization or prolongation of existing hospitalization
- a persistent or significant disability/incapacity
- a congenital anomaly/birth defect

Other important medical events may also be considered an SAE when, based on appropriate medical judgment, they jeopardize the subject or require intervention to prevent one of the outcomes listed.
A SAR is an SAE where a causal relationship between the IMP and the SAE is at least a reasonable possibility, i.e., the relationship cannot be ruled out.

9.1.3. Suspected Unexpected Serious Adverse Reactions (SUSARs)

AEs are SUSARs if the following three conditions are met:

- The event must be serious (see Section 9.1.2);
- There must be a certain degree of probability that the event is a harmful and an undesirable reaction to the IMP (see section 9.1.5), regardless of the administered dose;
- The adverse reaction must be unexpected, that is to say, the nature and severity of the adverse reaction are not in agreement with the product information as recorded in the Summary of Product Characteristics.

9.1.4. AE Severity

The National Cancer Institute’s Common Terminology Criteria for AEs (CTCAE) Version 4.03 should be used to assess and grade AE severity, including laboratory abnormalities judged to be clinically significant. The guidelines shown in Table 2 below should be used to grade severity. It should be pointed out that the term “severe” is a measure of intensity and that a severe AE is not necessarily serious.

Table 2. AE Severity Grading

Severity (Toxicity Grade)	Description
Mild (1)	Asymptomatic or mild symptoms; clinical or diagnostic observations only; intervention not indicated.
Moderate (2)	Moderate; minimal, local or non-invasive intervention indicated; limiting age-appropriate instrumental ADL*.
Severe (3)	Severe or medically significant but not immediately life-threatening; hospitalisation or prolongation of hospitalization indicated; disabling; limiting self-care ADL**.
Life-threatening (4)	Life-threatening consequences; urgent intervention indicated.
Death (5)	Death related to AE.

Activities of Daily Living (ADL)
*Instrumental ADL refer to preparing meals, shopping for groceries or clothes, using the telephone, managing money, etc.
**Self-care ADL refer to bathing, dressing and undressing, feeding self, using the toilet, taking medications, and not bedridden.

9.1.5. Relatedness of AE to IMP

The relationship of an AE to the IMP should be assessed using the following guidelines:

RELATED (Probable, Possible and/or Not-assessable are within the scope of Related)

An AE will be assessed as Related if any of the following applies to it:

1) An AE that is listed as a possible adverse reaction and cannot be reasonably explained by an alternative explanation (e.g.: concomitant drug, concomitant disease); and the relationship in time is very suggestive (e.g.: it is confirmed by dechallenge and rechallenge).
2) An AE that might be due to the use of the drug; the relationship in time is suggestive (e.g.: confirmed by dechallenge); and an alternative explanation is less likely (e.g.: concomitant drug, concomitant disease).
3) An AE that might be due to the drug; an alternative explanation (e.g.: concomitant drug, concomitant disease) is inconclusive; and the relationship in time is reasonable; therefore, the causal relationship cannot be excluded.
4) Not assessable.

NOT RELATED
An AE that is not related to the use of the IMP Treatment.

NOT APPLICABLE
Relationship to AE is not applicable if the patient has not received the substance as an IMP according to the corresponding treatment arm.

9.1.6. Outcome categorisation

The outcome of AEs must be recorded during the course of the study in the eCRF. Outcomes are as follows:

- Recovered/resolved
- Recovering/resolving

- Not recovered/not resolved/ ongoing
- Recovered/ resolved with sequelae
- Fatal
- Unknown

9.2. DOCUMENTATION AND REPORTING PROCEDURES

9.2.1. Reporting of AEs and SAEs

The Investigator will probe, via discussion with the subject, for the occurrence of AEs up to the visit of day 7. AEs will be described by duration (start and stop dates), severity, outcome, treatment and relation to the IMP, or if unrelated, the cause.

All AEs grade 3 to 5 will be documented in the site´s source documents and subject eCRF (whether or not related to the IMP). AEs grade 1 or 2 do not have to be documented in the eCRF or reported to the Study Safety Desk.

The recording and reporting period for all AEs (including SAEs) as defined in section 9.1.1 will begin after randomisation and end on day 7, except for SARs and SUSARs. Any AE that occurs for the first time after day 7 is highly unlikely to be related to any of the IMPs. However, any SAE occurring between day 7 and the end of follow-up on day 90 (± 14 days) for which a causal relationship between the IMP and the SAE is considered at least a reasonable possibility (i.e., SARs and SUSARs) should be reported as other SAEs (see below). In order to prevent redundant data entry, outcome events and endpoints of the clinical trial (e.g. death and infection) will be documented only as outcome events in the eCRF and not as AEs. 'Death' occurring before the final follow-up at day 90 (± 14) is a study outcome and should always be documented in the eCRF.

All SAEs, except for expected SAEs (see below), occurring between randomisation and day 7 have to be reported within 24 hours of Investigator's first awareness about the event, to the Study Safety Desk:

CTC North Safety Desk
pharmacovigilance@ctc-north.com

Expected SAEs are events that are known to occur in the condition under study or with the IMPs used in the trial as defined in their SmPCs. Expected SAEs are defined in the protocol (Appendices 7 and 8). Expected SAEs as well as study endpoints are excluded from expedited reporting but should be documented in the eCRF and reported to the Safety Desk within 7 days of the Investigator's first awareness about the event.

The report of the SAE must include an assessment of whether there is a reasonable possibility that the IMP caused the event.

All SAE follow-up reports must also be sent to the Study Safety Monitoring Department. For Follow-up reports a new SAE form should be filled in and sent to the Safety Monitoring Department as soon as possible. The Investigator must complete (in English language), sign, and date the SAE Form and report to the Study Safety Desk.

Death is an outcome of an event and must not be reported as an SAE term. The event causing the death must be reported as SAE. When the cause of death is not known, the Investigator should report these cases as "death of unknown cause".

In case of death related to the IMP, anonymised copies of the death certificate and of the autopsy report, when available, will be sent to the Study Safety Desk. Any other anonymised source documents related to the death should also be provided. In the event that no source documents are available, the investigator is required to describe the circumstances of the subject's death in a letter, email, or other written documentation.

The Sponsor shall ensure that all SAEs are reported to the Regulatory Authority and to the IEC according to local and national regulations. The Sponsor shall keep detailed records of all SAEs which are reported by the investigator or investigators for at least 15 years.

9.2.2. Reporting SUSARS

Depending on local and national regulations, the Investigator at each participating site is responsible for providing the local IEC with reports of any SUSAR from the study. The Sponsor will provide this information to the other Investigators so that he/she can meet these reporting requirements.
The Sponsor shall ensure that all relevant information about SUSARs that are fatal or life-threatening is recorded and reported as soon as possible to the Regulatory Authority and to the IEC, and in any case no later than 7 calendar days after knowledge by the Sponsor of such a case, and that relevant follow-up information is subsequently communicated within an additional 8 calendar days. All other SUSARs shall be reported to the Regulatory Authority and to the IEC as soon as possible but within a maximum of 15 calendar days of first knowledge by the Sponsor. The Sponsor shall also inform all Investigators.

10. END OF STUDY, SUBJECT WITHDRAWAL AND PREMATURE TERMINATION

10.1. END OF STUDY

If the Sponsor, the Investigator(s), or Regulatory Authorities discover any conditions during the study that indicate that the study or study site should be terminated, this action may be taken after appropriate consultation between the Sponsor and the Investigator(s).The Sponsor has the right to terminate the participation of either an individual site, or the study at any time. This action may be taken based on the recommendation of the independent DSMB.

Reasons for terminating the study may include, but are not limited to, the following:

- The incidence and severity of AEs in this or other studies indicates a potential health hazard to subjects.
- Subject enrolment is unsatisfactory.
- Data recording is inaccurate or incomplete.
- Investigator(s) do not adhere to the protocol or applicable regulatory guidelines in conducting this study.
- Submission of knowingly false information from the study site to the Sponsor or regulatory authorities.
- Results of a planned interim analysis support terminating the study.
- In the event that the study is terminated early, the Sponsor will provide specific guidance to investigational sites regarding the end-of-study procedures.

10.2. SUBJECT DISCONTINUATION OR WITHDRAWAL

The primary reason for discontinuation or withdrawal of the subject from the study should be recorded in the eCRF using the following categories:

- Death.
- Major protocol deviation. The discovery post-enrolment that the subject failed to meet protocol entry criteria or did not adhere to protocol requirements.
- Lost to follow-up: The subject did not return the clinic and attempts to contact the subject were unsuccessful. Attempts to contact the subject must be documented in the subject source.
- Voluntary withdrawal. The subject (or subject's legally acceptable representative) wishes to withdraw from the study. The reason for withdrawal, if provided, should be recorded in the eCRF. NOTE: All attempts should be made to determine the underlying reason for the withdrawal and, where possible, the primary underlying reason should be recorded (e.g. withdrawal due to an AE or lack of efficacy, confirmed by the investigator, should not be recorded in the "voluntary withdrawal" category).
- Study termination. The Sponsor, IEC, or regulatory agency terminates the study.
- Other. NOTE: the specific reasons should be recorded in the "specify" field of the eCRF.

The investigator may terminate a subject's study participation at any time during the study when the subject meets the study withdrawal criteria described above. In addition, subject may discontinue his or her participation without giving a reason at any time during the study. Should a subject's participation be discontinued, the primary criterion for termination must be recorded. In addition, efforts should be made to perform all procedures scheduled for the End of Follow-Up Visit (day 90 ± 14 days). An attempt should be undertaken to perform a Vital Status evaluation in all withdrawn subjects. A subject may choose to withdraw from the treatment or early assessment period of the study, or her/his proxy may choose that s/he be withdrawn, but be willing for the final follow-up assessment to be retained at 90 days, or even just to be contacted to assess vital status. A subject may

also choose to withdraw consent for taking and analysis of a rectal swab. In case of the last, any swab that has already been taken will be destroyed. If so, this will be documented and maximum information collected within the terms of consent.

10.3. REPLACEMENT OF SUBJECT

Subjects who withdraw from the study will not be replaced.

10.4. PREMATURE TERMINATION OF STUDY

If the Sponsor, the Investigator(s), or regulatory authorities discover any conditions during the study that indicate that the study or investigational site should be terminated, this action may be taken after appropriate consultation between the Sponsor and the Investigator(s). The Sponsor has the right to terminate the participation of either an individual site or the study at any time.

10.4.1. Criteria for Premature Termination or Suspension of the study

The study will be completed as planned unless one or more of the following criteria are satisfied that require temporary suspension or early termination of the study.

- New information or other evaluation regarding safety or efficacy of the IP indicates a change in the known risk/benefit profile for the compound; such the risk/benefit is no longer acceptable for subjects participating in the study.
- Significant violation of ICH-Good Clinical Practices (GCP) compromising the ability to achieve the primary study objectives or compromises subject's safety.
- In case of an unexpected high rate of related SAEs or SUSARs in planned or unplanned reviews of the DSMB may suggest to stop the trial or enrolment in one of the strata of the trial.

10.4.2. Criteria for Premature Termination or Suspension of investigational Sites

The study may be terminated prematurely if the site (including the investigator) is found in significant violation of ICH-GCP, protocol, or contractual agreement, is unable to ensure adequate performance of the study, or as otherwise permitted by the contractual agreement.

10.4.3. Procedures for Premature Termination or Suspension of study or Investigational Sites

In the case the Sponsor, an IEC (IEC) or regulatory authority elects to terminate or suspend the study or the participation of an investigational site, a study-specific procedure for early termination or suspension will be provided by the Sponsor; the procedure will be followed by applicable investigational site during the course or termination or study suspension.

11. DEVIATION & VIOLATION

11.1. DEVIATION

A deviation is an accidental or unintentional change to, or non-compliance with the research protocol that does not increase risk or decrease benefit or; does not have a significant effect on the subject's rights, safety or welfare and/or on the integrity of the data. Deviations may result from the action of the subject, researcher, or research staff.

11.2. VIOLATION

A violation is an accidental or unintentional change to, or non-compliance with the IEC approved protocol without prior Sponsor and IEC approval. Violations generally increase risk or decrease benefit, affect the subject's rights, safety, or welfare, or the integrity of the data. A violation is also present in cases of wilful or knowing misconduct and in cases of serious or continuing non-compliance with ICH-GCP, legislation and regulations.

11.3. FOLLOW-UP OF DEVIATIONS AND VIOLATIONS

In case of a deviation or violation that increases the risk for the subject or that affects the subject's rights or welfare or the integrity of the data, the investigator enters a comment in the source documents and the non-compliance will be documented in a Monitoring Visit Report by the CRA. All of this serious non-compliance will be followed up and reported to RA and IEC as per local regulations. These serious violations will also be captured on a Violation Form and signed off by the Sponsor and investigator. In parallel, corrective and/or preventive actions will be undertaken and documented, including any retraining of the investigator and site staff.

12. ETHICAL CONSIDERATIONS & PROTECTION OF HUMAN SUBJECTS

12.1. REGULATION STATEMENT

The investigator will ensure that this study is conducted in full conformance with the principles of the "Declaration of Helsinki" (64th WMA General Assembly, Fortaleza, Brazil, October 2013) or with the laws and regulations of the country in which the research is conducted, whichever affords the greater protection to the individual. The study must fully adhere to the principles outlined in "Guideline for Good Clinical Practice" ICH Tripartite Guideline or with local law if this affords greater protection to the subject. As this study is conducted in the EU/EEA countries, the investigator will ensure compliance with the EU Clinical Trial Directive [2001/20/EC].

12.2. REGULATORY AUTHORITY/IECS

The protocol and any accompanying material provided to the subject (such as subject information sheets or descriptions of the study used to obtain informed consent as well as any recruitment materials or compensation given to the subject) will be submitted to the RA and IEC.
Approval from the committee must be obtained before starting the study, and should be documented in a letter to the investigator specifying the date on which the committee met and granted the approval.
Any modifications made to the protocol after receipt of the RAs/IECs approval must be re-submitted in the EEA member states in accordance with local procedures and regulatory requirements.

12.3. INFORMED CONSENT

Informed consent will be obtained in accordance with the Declaration of Helsinki, ICH-GCP, the Data Protection Directive (Directive 95/46/EC), and national and local regulations. The Sponsor will prepare the informed consent form (ICF) and provide the documents to the RA and IEC for approval.
Before enrolment in the study, the investigator or an authorised member of the investigational staff must explain to potential study subjects and/or his/her legal representative the aims, methods, reasonably anticipated benefits, and potential hazards of the study, and any discomfort participation in the study may entail. Subjects will be informed their participation is voluntary and they may withdraw consent to participate at any time. They will be informed choosing not to participate will not affect the care the subject will receive for the treatment of his or her disease. Subjects will be told that alternative treatment strategies are available if they refuse to take part and that such refusal will not prejudice future treatments. Finally, they will be told the investigator will maintain a subject identification register for the purposes of long-term follow-up if needed and their medical records may be accessed by health authorities and authorised Sponsor staff without violating the confidentiality of the subject, to the extent permitted by the applicable law(s) or regulation.
By signing the ICF the subject is authorizing such access, and agrees to allow his or her study physician to re-contact the subject for the purpose of obtaining consent for additional safety evaluations if needed, or to obtain information about his or her vital status.
The subject and/or his/her legal representative or an independent physician (depending on national and local regulations) will be given sufficient time to read the ICF and the opportunity to ask questions. However, this time period is limited because trial treatment should be started within 24 hours of stroke onset. After this explanation and before entry into the study, consent should be appropriately recorded by means of the subject's or the legal representative's personally dated signature and

authorised study staff's personally dated signature. After obtaining the consent, a copy of the ICF must be given to the subject.

Copies or a second original of the signed ICF will be given to the subject and the original will be maintained with the subject's records. If new safety information results in significant changes in the risk/benefit assessment, the ICF should be reviewed and updated if necessary. All subjects should be informed of the new information and give their consent to continue the study.

12.4. AMENDMENTS

Protocol and/or ICF modifications or changes may not be initiated without prior written approval by the RA (where applicable) and IEC, except when necessary to eliminate immediate hazards to the subjects or when the change(s) involves only logistical or administrative aspects of the study. Such modifications will be submitted to the RA and IEC. A written verification that the modification was submitted and subsequently approved should be obtained.
The RA and IEC must be informed of revisions to other documents originally submitted for review; new information that may affect adversely the safety of the subjects of the conduct of the study, an annual update and/or request for re-approval.

12.5. END OF STUDY REPORT

The Sponsor will notify the IEC and the RA (if applicable) of the end of the study within a period of 90 days. The end of the study is defined as the last subject's last visit. In case the study is ended prematurely, the Sponsor will notify the IEC and the RA (if applicable) within 15 calendardays, including the reasons for the premature termination. Within one year after the end of the study, the investigator/Sponsor will submit a final study report with the results of the study, including any publications/abstracts of the study, to the IEC and the RA (if applicable).

12.6. COMPENSATION FOR INJURY

The Sponsor has insurance which is in accordance with the legal requirements. This insurance provides cover for damage to research subjects through injury or death caused by the study.

12.7. INCENTIVES

No incentives will be given to subjects for participation in this study. Reimbursement for travel to the investigational site and/or additional costs (including food and drink) incurred by the subject caused directly by the study or its procedures may be reimbursed at reasonable and fair terms.

13. PUBLICATION

The trial has been registered at the European Union Drug Regulating Authorities Clinical Trials (EudraCT) and the International Standard Randomized Controlled Trial Number (ISRCTN) registry before recruitment of patients started. This registry has been approved by the International Committee of Medical Journal Editors.

The results of the study will be reported in a Study Report generated by the Sponsor or Designee and will contain eCRF data from all investigational sites participating in the study. The research data will be publicly disclosed and published independent of the outcome of the study in scientific, peer-reviewed, international journals and at international conferences. The Sponsor reserves the right to analyse data for development and intellectual property purposes before public disclosure.

The contribution of ECRIN and its partners will be fairly described in the main publications of the results of the trial.

To promote the independent re-use of PRECIOUS data and to save the costs of unnecessarily compiling new datasets, access to a clean, anonymous, and well-annotated dataset will be made publicly available via the PRECIOUS website and in a public data repository within 18 months of the final follow-up of the last patient. Anonymous data will also be included in the Virtual International Stroke Trials Archive (VISTA; http://www.vista.gla.ac.uk/). This will allow the PRECIOUS investigators sufficient time to explore the datasets, balanced by the public interest of timely access. This sharing of participant-level data will provide others the opportunity to examine new research questions and will therefore increase the impact of PRECIOUS.

14. REFERENCES

1. Lozano R, Naghavi M, Foreman K et al. Global and regional mortality from 235 causes of death for 20 age groups in 1990 and 2010: a systematic analysis for the Global Burden of Disease Study 2010. Lancet 2012;380(9859):2095-2128.
2. Murray CJ, Vos T, Lozano R et al. Disability-adjusted life years (DALYs) for 291 diseases and injuries in 21 regions, 1990-2010: a systematic analysis for the Global Burden of Disease Study 2010. Lancet 2012;380(9859):2197-2223.
3. Van der Worp HB, Van Gijn J. Clinical practice. Acute ischemic stroke. N Engl J Med 2007;357(6):572-579.
4. Donnan GA, Fisher M, Macleod M, Davis SM. Stroke. Lancet 2008;371(9624):1612-1623.
5. Truelsen T, Piechowski-Jozwiak B, Bonita R, Mathers C, Bogousslavsky J, Boysen G. Stroke incidence and prevalence in Europe: a review of available data. Eur J Neurol 2006;13(6):581-598.
6. Feigin VL, Lawes CM, Bennett DA, Barker-Collo SL, Parag V. Worldwide stroke incidence and early case fatality reported in 56 population-based studies: a systematic review. Lancet Neurol 2009;8(4):355-369.
7. Heuschmann PU, Wiedmann S, Wellwood I et al. Three-month stroke outcome: the European Registers of Stroke (EROS) investigators. Neurology 2011;76(2):159-165.
8. Patel MD, Tilling K, Lawrence E, Rudd AG, Wolfe CD, McKevitt C. Relationships between long-term stroke disability, handicap and health-related quality of life. Age Ageing 2006;35(3):273-279.
9. Sprigg N, Selby J, Fox L, Berge E, Whynes D, Bath PM. Very low quality of life after acute stroke: data from the Efficacy of Nitric Oxide in Stroke trial. Stroke 2013;44(12):3458-3462.
10. Nichols M, Townsend N, Scarborough P, Rayner M. Cardiovascular disease in Europe: epidemiological update. Eur Heart J 2013;34(39):3028-3034.
11. Feigin VL, Forouzanfar MH, Krishnamurthi R et al. Global and regional burden of stroke during 1990-2010: findings from the Global Burden of Disease Study 2010. Lancet 2014;383(9913):245-254.
12. Feigin VL, Lawes CM, Bennett DA, Anderson CS. Stroke epidemiology: a review of population-based studies of incidence, prevalence, and case-fatality in the late 20th century. Lancet Neurol 2003;2(1):43-53.
13. Rabinstein A, Rundek T. Prediction of outcome after ischemic stroke: the value of clinical scores. Neurology 2013;80(1):15-16.
14. Poon MT, Fonville AF, Al-Shahi SR. Long-term prognosis after intracerebral haemorrhage: systematic review and meta-analysis. J Neurol Neurosurg Psychiatry 2014;85(6):660-667.
15. Guidelines for management of ischaemic stroke and transient ischaemic attack 2008. Cerebrovasc Dis 2008;25(5):457-507.
16. Jauch EC, Saver JL, Adams HP, Jr. et al. Guidelines for the early management of patients with acute ischemic stroke: a guideline for healthcare professionals from the American Heart Association/American Stroke Association. Stroke 2013;44(3):870-947.
17. Morgenstern LB, Hemphill JC, III, Anderson C et al. Guidelines for the management of spontaneous intracerebral hemorrhage: a guideline for healthcare professionals from the American Heart Association/American Stroke Association. Stroke 2010;41(9):2108-2129.
18. Steiner T, Al-Shahi SR, Beer R et al. European Stroke Organisation (ESO) guidelines for the management of spontaneous intracerebral hemorrhage. Int J Stroke 2014;9(7):840-855.

19. Sandercock PA, Counsell C, Tseng MC, Cecconi E. Oral antiplatelet therapy for acute ischaemic stroke. Cochrane Database Syst Rev 2014;3:CD000029.
20. Wardlaw JM, Murray V, Berge E, del Zoppo GJ. Thrombolysis for acute ischaemic stroke. Cochrane Database Syst Rev 2014;7:CD000213.
21. Berkhemer OA, Fransen PS, Beumer D et al. A randomized trial of intraarterial treatment for acute ischemic stroke. N Engl J Med 2015;372(1):11-20.
22. Saver JL, Goyal M, Bonafe A et al. Stent-Retriever Thrombectomy after Intravenous t-PA vs. t-PA Alone in Stroke. N Engl J Med 2015;372:2285-2295.
23. Campbell BC, Mitchell PJ, Kleinig TJ et al. Endovascular Therapy for Ischemic Stroke with Perfusion-Imaging Selection. N Engl J Med 2015;372:1009-1018.
24. Jovin TG, Chamorro A, Cobo E et al. Thrombectomy within 8 Hours after Symptom Onset in Ischemic Stroke. N Engl J Med 2015;372:2296-2306.
25. Goyal M, Demchuk AM, Menon BK et al. Randomized Assessment of Rapid Endovascular Treatment of Ischemic Stroke. N Engl J Med 2015;372:1019-1030.
26. Hofmeijer J, Kappelle LJ, Algra A, Amelink GJ, van GJ, Van der Worp HB. Surgical decompression for space-occupying cerebral infarction (the Hemicraniectomy After Middle Cerebral Artery infarction with Life-threatening Edema Trial [HAMLET]): a multicentre, open, randomised trial. Lancet Neurol 2009;8(4):326-333.
27. Juttler E, Unterberg A, Woitzik J et al. Hemicraniectomy in older patients with extensive middle-cerebral-artery stroke. N Engl J Med 2014;370(12):1091-1100.
28. Organised inpatient (stroke unit) care for stroke. Cochrane Database Syst Rev 2013;9:CD000197.
29. Anderson CS, Chalmers J, Stapf C. Blood-pressure lowering in acute intracerebral hemorrhage. N Engl J Med 2013;369(13):1274-1275.
30. Wahlgren N, Ahmed N, Davalos A et al. Thrombolysis with alteplase for acute ischaemic stroke in the Safe Implementation of Thrombolysis in Stroke-Monitoring Study (SITS-MOST): an observational study. Lancet 2007;369(9558):275-282.
31. Kumar S, Selim MH, Caplan LR. Medical complications after stroke. Lancet Neurol 2010;9(1):105-118.
32. Hesse K, Fulton RL, Abdul-Rahim AH, Lees KR. Characteristic Adverse Events and Their Incidence Among Patients Participating in Acute Ischemic Stroke Trials. Stroke 2014;45:2677-2682.
33. Westendorp WF, Nederkoorn PJ, Vermeij JD, Dijkgraaf MG, van de Beek D. Post-stroke infection: a systematic review and meta-analysis. BMC Neurol 2011;11:110.
34. den HH, van der WB, van GM, Dippel D. Therapeutic hypothermia in acute ischemic stroke. Expert Rev Neurother 2007;7(2):155-164.
35. Greer DM, Funk SE, Reaven NL, Ouzounelli M, Uman GC. Impact of fever on outcome in patients with stroke and neurologic injury: a comprehensive meta-analysis. Stroke 2008;39(11):3029-3035.
36. Saini M, Saqqur M, Kamruzzaman A, Lees KR, Shuaib A. Effect of hyperthermia on prognosis after acute ischemic stroke. Stroke 2009;40(9):3051-3059.
37. Martino R, Foley N, Bhogal S, Diamant N, Speechley M, Teasell R. Dysphagia after stroke: incidence, diagnosis, and pulmonary complications. Stroke 2005;36(12):2756-2763.
38. Vermeij FH, Scholte op Reimer WJ, de MP et al. Stroke-associated infection is an independent risk factor for poor outcome after acute ischemic stroke: data from the Netherlands Stroke Survey. Cerebrovasc Dis 2009;27(5):465-471.
39. Reith J, Jørgensen S, Pedersen PM et al. Body temperature in acute stroke: relation to stroke severity, infarct size, mortality, and outcome. Lancet 1996;347:422-425.

40. den Hertog HM, Van der Worp HB, van Gemert HM et al. An early rise in body temperature is related to unfavorable outcome after stroke: data from the PAIS study. J Neurol 2011;258(2):302-307.
41. Govan L, Langhorne P, Weir CJ. Does the prevention of complications explain the survival benefit of organized inpatient (stroke unit) care?: further analysis of a systematic review. Stroke 2007;38(9):2536-2540.
42. Ciccone A, Celani MG, Chiaramonte R, Rossi C, Righetti E. Continuous versus intermittent physiological monitoring for acute stroke. Cochrane Database Syst Rev 2013;5:CD008444.
43. Ntaios G, Dziedzic T, Michel P et al. European Stroke Organisation (ESO) guidelines for the management of temperature in patients with acute ischemic stroke. Int J Stroke 2015;10(6):941-949.
44. Middleton S, McElduff P, Ward J et al. Implementation of evidence-based treatment protocols to manage fever, hyperglycaemia, and swallowing dysfunction in acute stroke (QASC): a cluster randomised controlled trial. Lancet 2011;378(9804):1699-1706.
45. Westendorp WF, Vermeij JD, Vermeij F et al. Antibiotic therapy for preventing infections in patients with acute stroke. Cochrane Database Syst Rev 2012;1:CD008530.
46. den Hertog HM, Van der Worp HB, van Gemert HM et al. The Paracetamol (Acetaminophen) In Stroke (PAIS) trial: a multicentre, randomised, placebo-controlled, phase III trial. Lancet Neurol 2009;8(5):434-440.
47. Warusevitane A, Karunatilake D, Sim J, Lally F, Roffe C. Safety and effect of metoclopramide to prevent pneumonia in patients with stroke fed via nasogastric tubes trial. Stroke 2015;46(2):454-460.
48. Westendorp WF, Vermeij JD, Zock E et al. The Preventive Antibiotics in Stroke Study (PASS): a pragmatic randomised open-label masked endpoint clinical trial. Lancet 2015;385:1519-1526.
49. Kalra L, Irshad S, Hodsoll J et al. Prophylactic antibiotics after acute stroke for reducing pneumonia in patients with dysphagia (STROKE-INF): a prospective, cluster-randomised, open-label, masked endpoint, controlled clinical trial. Lancet 2015.
50. Meisel A, Smith CJ. Prevention of stroke-associated pneumonia: where next? Lancet 2015.
51. den Hertog HM, Van der Worp HB, Tseng MC, Dippel DW. Cooling therapy for acute stroke. Cochrane Database Syst Rev 2009;(1):CD001247.
52. van de Beek D, Wijdicks EF, Vermeij FH et al. Preventive antibiotics for infections in acute stroke: a systematic review and meta-analysis. Arch Neurol 2009;66(9):1076-1081.
53. van Breda EJ, van der Worp B, van GM et al. PISA. The effect of paracetamol (acetaminophen) and ibuprofen on body temperature in acute stroke: protocol for a phase II double-blind randomised placebo-controlled trial [ISRCTN98608690]. BMC Cardiovasc Disord 2002;2:7.
54. Appelbaum PS, Grisso T. Assessing patients' capacities to consent to treatment. N Engl J Med 1988;319(25):1635-1638.
55. Sessums LL, Zembrzuska H, Jackson JL. Does this patient have medical decision-making capacity? JAMA 2011;306(4):420-427.
56. Hemphill JC, III, Bonovich DC, Besmertis L, Manley GT, Johnston SC. The ICH score: a simple, reliable grading scale for intracerebral hemorrhage. Stroke 2001;32(4):891-897.
57. Sanossian N, Ovbiagele B. Prevention and management of stroke in very elderly patients. Lancet Neurol 2009;8(11):1031-1041.
58. van Swieten JC, Koudstaal PJ, Visser MC, Schouten HJ, Van Gijn J. Interobserver agreement for the assessment of handicap in stroke patients. Stroke 1988;19(5):604-607.
59. Lees KR, Bath PM, Schellinger PD et al. Contemporary outcome measures in acute stroke research: choice of primary outcome measure. Stroke 2012;43(4):1163-1170.

60. Mahoney FI, Barthel DW. Functional evaluation. The Barthel Index. MD State Med J 1965;14:61-65.
61. Nasreddine ZS, Phillips NA, Bedirian V et al. The Montreal Cognitive Assessment, MoCA: a brief screening tool for mild cognitive impairment. J Am Geriatr Soc 2005;53(4):695-699.
62. Quinn TJ, Dawson J, Lees JS, Chang TP, Walters MR, Lees KR. Time spent at home poststroke: "home-time" a meaningful and robust outcome measure for stroke trials. Stroke 2008;39(1):231-233.
63. Brott T, Adams HP, Jr., Olinger CP et al. Measurements of acute cerebral infarction: a clinical examination scale. Stroke 1989;20:864-870.
64. Sandercock P, Wardlaw JM, Lindley RI et al. The benefits and harms of intravenous thrombolysis with recombinant tissue plasminogen activator within 6 h of acute ischaemic stroke (the third international stroke trial [IST-3]): a randomised controlled trial. Lancet 2012;379(9834):2352-2363.
65. Murray GD, Barer D, Choi S et al. Design and analysis of phase III trials with ordered outcome scales: the concept of the sliding dichotomy. J Neurotrauma 2005;22(5):511-517.
66. Bath PM, Lees KR, Schellinger PD et al. Statistical analysis of the primary outcome in acute stroke trials. Stroke 2012;43(4):1171-1178.
67. Horan TC, Andrus M, Dudeck MA. CDC/NHSN surveillance definition of health care-associated infection and criteria for specific types of infections in the acute care setting. Am J Infect Control 2008;36(5):309-332.
68. Westendorp WF, Vermeij JD, Dippel DW et al. Update of the Preventive Antibiotics in Stroke Study (PASS): statistical analysis plan. Trials 2014;15:382.
69. Smith CJ, Kishore AK, Vail A et al. Diagnosis of Stroke-Associated Pneumonia: Recommendations From the Pneumonia in Stroke Consensus Group. Stroke 2015;46(8):2335-2340.
70. Pendlebury ST, Mariz J, Bull L, Mehta Z, Rothwell PM. MoCA, ACE-R, and MMSE versus the National Institute of Neurological Disorders and Stroke-Canadian Stroke Network Vascular Cognitive Impairment Harmonization Standards Neuropsychological Battery after TIA and stroke. Stroke 2012;43(2):464-469.
71. Lees R, Selvarajah J, Fenton C et al. Test accuracy of cognitive screening tests for diagnosis of dementia and multidomain cognitive impairment in stroke. Stroke 2014;45(10):3008-3018.
72. Granger CV, Dewis LS, Peters NC, Sherwood CC, Barrett JE. Stroke rehabilitation: analysis of repeated Barthel index measures. Arch Phys Med Rehabil 1979;60(1):14-17.
73. Sulter G, Steen C, De KJ. Use of the Barthel index and modified Rankin scale in acute stroke trials. Stroke 1999;30(8):1538-1541.
74. Dorman PJ, Slattery J, Farrell B, Dennis MS, Sandercock PA. A randomised comparison of the EuroQol and Short Form-36 after stroke. United Kingdom collaborators in the International Stroke Trial. B M J 1997;315(7106):461.
75. Balami JS, Buchan AM. Complications of intracerebral haemorrhage. Lancet Neurol 2012;11(1):101-118.
76. Balami JS, Chen RL, Grunwald IQ, Buchan AM. Neurological complications of acute ischaemic stroke. Lancet Neurol 2011;10(4):357-371.

15. APPENDICES

Appendix 1 – Common Terminology Criteria for AEs (CTCAE) version 4.03

Quick Reference
The NCI Common Terminology Criteria for AEs is a descriptive terminology which can be utilised for AE (AE) reporting. A grading (severity) scale is provided for each AE term.

Components and Organisation
System Organ Class (SOC), the highest level of the Medical Dictionary for Regulatory Activities (MedDRA) hierarchy, is identified by anatomical or physiological system, aetiology, or purpose (e.g. SOC Investigations for laboratory test results). CTCAE terms are grouped by MedDRA Primary SOCs. Within each SOC, AEs are listed and accompanied by descriptions of severity (Grade).

CTCAE Terms
An AE (AE) is any unfavourable and unintended sign (including an abnormal laboratory finding), symptom, or disease temporally associated with the use of a medical treatment or procedure that may or may *not* be considered related to the medical treatment or procedure. An AE is a term that is a unique representation of a specific event used for medical documentation and scientific analyses. Each CTCAE v4.03 term is a MedDRA LLT (Lowest Level Term).

Definitions
A brief definition is provided to clarify the meaning of each AE term.

Grades
Grade refers to the severity of the AE. The CTCAE displays Grades 1 through 5 with unique clinical descriptions of severity for each AE based on this general guideline:
Grade 1 Mild; asymptomatic or mild symptoms; clinical or diagnostic observations only; intervention not indicated.
Grade 2 Moderate; minimal, local or non-invasive intervention indicated; limiting age-appropriate instrumental ADL*.
Grade 3 Severe or medically significant but not immediately life-threatening; hospitalisation or prolongation of hospitalisation indicated; disabling; limiting self-care ADL**.
Grade 4 Life-threatening consequences; urgent intervention indicated.
Grade 5 Death related to AE.

Activities of Daily Living (ADL)
*Instrumental ADL refer to preparing meals, shopping for groceries or clothes, using the telephone, managing money, etc.
**Self-care ADL refer to bathing, dressing and undressing, feeding self, using the toilet, taking medications, and not bedridden.
The complete CTCAE document can be found on the following website and will be provided to sites:
http://evs.nci.nih.gov/ftp1/CTCAE/About.html

Appendix 2 – National Institutes of Health Stroke Scale (NIHSS)

The NIHSS is an ordinal hierarchical scale to evaluate the severity of stroke by assessing a patient's performance.[63] Scores range from 0 to 42, with higher scores indicating a more severe deficit.

Administer stroke scale items in the order listed. Record performance in each category after each subscale exam. Do not go back and change scores. Follow directions provided for each exam technique. Scores should reflect what the patient does, not what the clinician thinks the patient can do. The clinician should record answers while administering the exam and work quickly. Except where indicated, the patient should not be coached (i.e. repeated requests to patient to make a special effort).

Instructions	Scale definition	Score
**1a. Level of consciousness**. The investigator must choose a response if a full evaluation is prevented by such obstacles as an endotracheal tube, language barrier, orotracheal trauma/bandages. A 3 is scored only if the patient makes no movement (other than reflexive posturing) in response to noxious stimulation.	0 = **Alert**; keenly responsive.<br>1 = **Not alert**; but arousable by minor stimulation to obey, answer, or respond.<br>2 = **Not alert**; required repeated stimulation to attend, or is obtunded and requires strong or painful stimulation to make movements (not stereotyped).<br>3 = Responds only with reflex motor or autonomic effects or totally unresponsive, flaccid and areflexic.	------------
**1b. LOC Questions**: The patient is asked the month and his/her age. The answer must be correct – there is not partial credit for being close. Aphasic and stuporous patients who do not comprehend the questions will score 2. Patients unable to speak because of endotracheal intubation, orotracheal trauma, severe dysarthria from any cause, language barrier, or any other problem not secondary to aphasia are given a 1. It is important that only the initial answer be graded and that the examiner not "help" the patient with verbal or non-verbal clues.	0 = **Answers** both questions correctly.<br>1 = **Answers** one question correctly.<br>2 = **Answers** neither question correctly.	------------
**1c. LOC Commands**: The patient is asked to open and close the eyes and then to grip and release the non-paretic hand. Substitute another one step command if the hands cannot be used. Credit is given if an unequivocal attempt is made but not completed due to weakness. If the patient does not respond to command, the task should be demonstrated to him or her (pantomime), and the result scored (i.e. follows none, one or two commands). Patients with trauma, amputation, or other physical impediments should be given suitable one-step commands. Only the first attempt is scored.	0 = **Performs** both tasks correctly.<br>1 = **Performs** one task correctly.<br>2 = **Performs** neither task correctly.	------------

**2. Best Gaze:** Only horizontal eye movements will be tested. Voluntary or reflexive (oculocephalic) eye movements will be scored, but caloric testing is not done. If the patient has a conjugate deviation of the eyes that can be overcome by voluntary or reflexive activity, the score will be a 1. If a patient has an isolated peripheral nerve paresis (CN III, IV or VI), score a 1. Gaze is testable in all aphasic patients. Patients with ocular trauma, bandages, pre-existing blindness, or other disorder of visual acuity or fields should be tested with reflexive movements, and a choice made by the investigator. Establishing eye contact and then moving about the patient from side to side will occasionally clarify the presence of a partial gaze palsy.	0 = **Normal**.<br><br>1 = **Partial gaze palsy**; gaze is abnormal in one or both eyes, but forced deviation or total gaze paresis is not present.<br><br>2 = **Forced deviation;** or total gaze paresis not overcome by the oculocephalic maneuver.	------------
**3. Visual**. Visual fields (upper and lower quadrants) are tested by confrontation, using finger counting or visual threat, as appropriate. Patients may be encouraged, but if they look at the side of the moving finger appropriately, this can be scored as normal. If there is unilateral blindness or enucleation, visual fields in the remaining eye are scored. Score 1 only if a clear-cut asymmetry, including quadrantanopia, is found. If patient is blind from any cause, score 3. Double simultaneous stimulation is performed at this point. If there is extinction, patients receives a 1, and the results are used to respond to item 11.	0 = **No visual loss**.<br><br>1 = **Partial hemianopia**.<br><br>2 = **Complete hemianopia**.<br><br>3 = **Bilateral hemianopia** (blind including cortical blindness)	------------
**4. Facial Palsy**: Ask – or use pantomime to encourage – the patient to show teeth or raise eyebrows and close eyes. Score symmetry of grimace in response to noxious stimuli in the poorly response or non-comprehending patient. If facial trauma/bandages, orotracheal tube, tape or other physical barriers obscure the face, these should be removed to the extent possible.	0 = **Normal** symmetrical movements.<br>1 = **Minor paralysis** (flattened nasolabial fold, asymmetry on smiling)<br>2 = **Partial paralysis** (total or near-total paralysis of lower face)<br>3 = **Complete paralysis** of one or both sides (absence of facial movement in the upper and lower face).	------------
**5. Motor arm**: The limb is placed in the appropriate position: extend the arms (palms down) 90 degrees (if sitting) or 45 degrees (if supine). Drift is scored if the arm falls before 10 seconds. The aphasic patient is encouraged using urgency in the voice and pantomime, but not noxious stimulation. Each limb is tested in turn, beginning with the non-paretic arm. Only in the case of amputation or joint fusion at the shoulder, the examiner should record the score as untestable (UN), and clearly write the explanation for this choice.	0 = **No drift**; limb holds 90 (or 45) degrees for full 10 seconds.<br>1 = **Drift**; limb holds 90 (or 45) degrees, but drifts down before full 10 seconds; does not hit bed or other support.<br>2 = **Some effort against gravity**; limb cannot get to or maintain (if cued) 90 (or 45) degrees, drifts down to bed, but has some effort against gravity.<br>3 = **No effort against gravity**; limb falls.<br>4 = **No movement**.<br>UN = **Amputation** or joint fusion: explain:<br><br>**5a = Left Arm**.<br><br>**5b = Right arm**.	------------<br><br>------------

**6. Motor leg**: The limb is placed in the appropriate position: hold the leg at 30 degrees (always tested supine). Drift is scored if the leg falls before 5 seconds. The aphasic patient is encouraged using urgency in the voice and pantomime, but not noxious stimulation. Each limb is tested in turn, beginning with the non-paretic leg. Only in the case of amputation or joint fusion at the hip, the examiner should record the score as untestable (UN), and clearly write the explanation for this choice.	0 = **No drift**; leg holds 30-degree position for full 5 seconds.<br>1 = **Drift**; leg falls by the end of the 5-second period but does not hit bed.<br>2 = **Some effort against gravity**; leg falls to bed by 5 seconds, but has some effort against gravity.<br>3 = **No effort against gravity**; leg falls to bed immediately.<br>4 = No movement.<br>UN = Amputation or joint fusion, explain:<br><br>**6a. Left Leg**<br><br>**6b. Right Leg.**	------------<br><br>------------
**7. Limb ataxia**: This item is aimed at finding evidence of a unilateral cerebellar lesion. Test with eyes open. In case of visual defect, ensure testing is done in intact visual field. The finger-nose-finger and heel-shin tests are performed on both sides, and ataxia is scored only if present out of proportion to weakness. Ataxia is absent in the patient who cannot understand or is paralyzed. Only in the case of amputation or joint fusion, the examiner should record the score as untestable (UN), and clearly write the explanation for this choice. In case of blindness, test by having the patient touch nose from extended arm position.	0 = **Absent**.<br><br>1 = **Present in one limb**.<br><br>2 = **Present in two limbs**.<br><br>UN = **Amputation** or joint fusion, explain:	------------
**8. Sensory**: Sensation or grimace to pinprick when tested, or withdrawal from noxious stimulus in the obtunded or aphasic patient. Only sensory loss attributed to stroke is scored as abnormal and the examiner should test as many body areas (arms [not hands], legs, trunk, face) as needed to accurately check for hemisensory loss. A score of 2, 'severe or total sensory loss', should only be given when a severe or total loss of sensation can be clearly demonstrated. Stuporous and aphasic patients will, therefore, probably score 1 or 0. The patient with brainstem stroke who has bilateral loss of sensation is scored 2. If the patient does not respond and is quadriplegic, score 2. Patients in a coma (item 1a=3) are automatically given a 2 on this item.	0 = **Normal**; no sensory loss.<br><br>1 = **Mild-to-moderate sensory loss**; patients feels pinprick is less sharp or is dull on the affected side; or there is a loss of superficial pain with pinprick, but patient is aware of being touched.<br><br>2 = **Severe to total sensory loss**; patient is not aware of being touched in the face, arm and leg.	------------

**9. Best language**: A great deal of information about comprehension will be obtained during the preceding sections of the examination. For this scale item, the patient is asked to describe what is happening in the attached picture, to name the items on the attached naming sheet and to read from the attached list of sentences. Comprehension is judged from responses here, as well as to all of the commands in the preceding general neurological exam. If visual loss interferes with the tests, ask the patient to identify objects placed in the hand, repeat, and produce speech. The intubated patient should be asked to write. The patient in a coma (item 1a=3) will automatically score 3 on this item. The examiner must choose a score for the patient with stupor or limited cooperation, but a score of 3 should be used only if the patient is mute and follows no one-step commands.	0 = **No aphasia**; normal<br>1 = **Mild-to-moderate aphasia**; some obvious loss of fluency or facility of comprehension, without significant limitation on ideas expressed or form of expression. Reduction of speech and/or comprehension, however, makes conservation about provided materials difficult or impossible. For example, in conversation about provided materials, examiner can identify picture or naming card content from patient's response.<br>2 = **Severe aphasia**; all communication is through fragmentary expression; great need for inference, questioning, and guessing by the listener. Range of information that can be exchanged is limited; listener carries burden of communication. Examiner cannot identify materials provided from patient response.<br>3 = **Mute, global aphasia**: no usable speech or auditory comprehension.	------------
**10. Dysarthria**: If patient is thought to be normal, an adequate sample of speech must be obtained by asking patient to read or repeat words from the attached list. If the patient has severe aphasia, the clarity of articulation of spontaneous speech can be rated. Only if patient is intubated or has other physical barriers to producing speech, the examiner should record the score as untestable (UN), and clearly write an explanation for this choice. Do not tell the patient why he or she is being tested.	0 = **Normal**.<br>1 = **Mild-to-moderate dysarthria**; patient slurs at least some words and, at worst, can be understood by some difficulty.<br>2 = **Severe dysarthria**: patient's speech is so slurred as to be unintelligible in the absence of or out of proportion to any dysphasia, or is mute/anarthric.<br>UN = **Intubated** or other physical barrier. Explain:	------------
**11. Extinction and Inattention (formerly Neglect)**: Sufficient information to identify neglect may be obtained during the prior testing. If the patient has a severe visual loss preventing visual double simultaneous stimulation, and the cutaneous stimuli are normal, the score is normal. If the patient has aphasia but does appear to attend to both sides, the score is normal. The presence of visual spatial neglect or anosagnosia may also be taken as evidence of abnormality. Since the abnormality is scored only if present, the item is never untestable.	0 = **No abnormality**.<br>1 = **Visual, tactile, auditory, spatial, or personal inattention** or extinction to bilateral simultaneous stimulation in one of the sensory modalities.<br>2 = **Profound hemi-inattention or extinction to more than one modality**; does not recognize own hand or orients to only one side of space.	------------

Appendix 3 – modified Rankin Scale (mRS)

The mRS is an ordinal hierarchical scale ranging from 0 to 5, with higher scores indicating more severe disability. A score of 6 has been added to signify death.

0. No symptoms.
1. No significant disability. Able to carry out all usual activities despite some symptoms.
2. Slight disability. Able to look after own affairs without assistance, but unable to carry out all previous activities.
3. Moderate disability. Requires some help, but able to walk unassisted.
4. Moderately severe disability. Unable to attend to own body needs without assistance and unable to walk unassisted.
5. Severe disability. Requires constant nursing care and attention, bedridden, incontinent.
6. Dead.

Appendix 4 – Montreal Cognitive Assessment (MoCa)

The Montreal Cognitive Assessment (MoCA) is a short cognitive test for use in patients with stroke and other neurological conditions.[61] Test scores range from 0 to 30, with higher scores indicating better cognitive performance. The test is available in 46 languages and dialects [www.mocatest.org]. An English example is provided below.

MONTREAL COGNITIVE ASSESSMENT (MOCA)
Version 7.1 Original Version

NAME :
Education : Date of birth :
Sex : DATE :

VISUOSPATIAL / EXECUTIVE

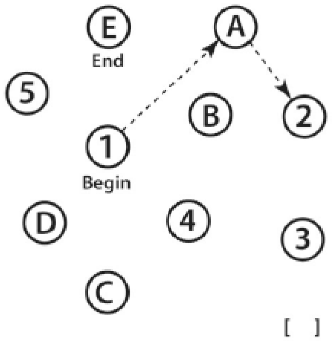

[ ]

Copy cube

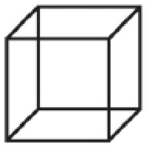

[ ]

Draw CLOCK (Ten past eleven) (3 points)

[ ] Contour [ ] Numbers [ ] Hands

POINTS: ___/5

NAMING

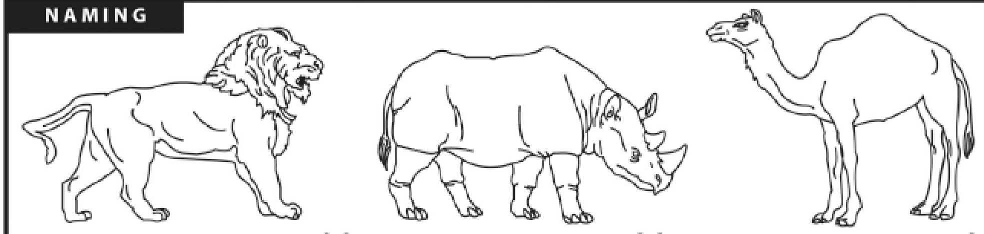

[ ] [ ] [ ] ___/3

MEMORY Read list of words, subject must repeat them. Do 2 trials, even if 1st trial is successful. Do a recall after 5 minutes.

	FACE	VELVET	CHURCH	DAISY	RED
1st trial					
2nd trial					

No points

ATTENTION Read list of digits (1 digit/ sec.). Subject has to repeat them in the forward order [ ] 2 1 8 5 4
Subject has to repeat them in the backward order [ ] 7 4 2 ___/2

Read list of letters. The subject must tap with his hand at each letter A. No points if ≥ 2 errors
[ ] F B A C M N A A J K L B A F A K D E A A A J A M O F A A B ___/1

Serial 7 subtraction starting at 100 [ ] 93 [ ] 86 [ ] 79 [ ] 72 [ ] 65
4 or 5 correct subtractions: **3 pts**, 2 or 3 correct: **2 pts**, 1 correct: **1 pt**, 0 correct: **0 pt** ___/3

LANGUAGE Repeat : I only know that John is the one to help today. [ ]
The cat always hid under the couch when dogs were in the room. [ ] ___/2

Fluency / Name maximum number of words in one minute that begin with the letter F [ ] ____ (N ≥ 11 words) ___/1

ABSTRACTION Similarity between e.g. banana - orange = fruit [ ] train – bicycle [ ] watch - ruler ___/2

DELAYED RECALL	Has to recall words WITH NO CUE	FACE [ ]	VELVET [ ]	CHURCH [ ]	DAISY [ ]	RED [ ]	Points for UNCUED recall only	___/5
Optional	Category cue							
	Multiple choice cue							

ORIENTATION [ ] Date [ ] Month [ ] Year [ ] Day [ ] Place [ ] City ___/6

© Z.Nasreddine MD www.mocatest.org Normal ≥26 / 30 TOTAL ___/30
Administered by: ______________________
Add 1 point if ≤ 12 yr edu

Appendix 5 – Barthel Index (BI)

The Barthel Index (BI) is an ordinal scale used to measure performance in 10 activities of daily living (ADL).[60, 72, 73] Test scores range from 0 to 100, with higher scores indicating better performance in these activities.

FEEDING
0 = unable
5 = needs help cutting, spreading butter, etc., or requires modified diet
10 = independent
BATHING
0 = dependent
5 = independent (or in shower)
GROOMING
0 = needs to help with personal care
5 = independent face/hair/teeth/shaving (implements provided)
DRESSING
0 = dependent
5 = needs help but can do about half unaided
10 = independent (including buttons, zips, laces, etc.)
BOWELS
0 = incontinent (or needs to be given enemas)
5 = occasional accident
10 = continent
BLADDER
0 = incontinent, or catheterized and unable to manage alone
5 = occasional accident
10 = continent
TOILET USE
0 = dependent
5 = needs some help, but can do something alone
10 = independent (on and off, dressing, wiping)
TRANSFERS (BED TO CHAIR AND BACK)
0 = unable, no sitting balance
5 = major help (one or two people, physical), can sit
10 = minor help (verbal or physical)
15 = independent
MOBILITY (ON LEVEL SURFACES)
0 = immobile or < 50 yards
5 = wheelchair independent, including corners, > 50 yards
10 = walks with help of one person (verbal or physical) > 50 yards
15 = independent (but may use any aid; for example, stick) > 50 yards
STAIRS
0 = unable
5 = needs help (verbal, physical, carrying aid)
10 = independent

Guidelines

1. The index should be used as a record of what a patient does, not as a record of what a patient could do.
2. The main aim is to establish degree of independence from any help, physical or verbal, however minor and for whatever reason.
3. The need for supervision renders the patient not independent.
4. A patient's performance should be established using the best available evidence. Asking the patient, friends/relatives and nurses are the usual sources, but direct observation and common sense are also important. However direct testing is not needed.
5. Usually the patient's performance over the preceding 24-48 hours is important, but occasionally longer periods will be relevant.
6. Middle categories imply that the patient supplies over 50 per cent of the effort.
7. Use of aids to be independent is allowed.

Appendix 6 – EuroQoL

The EuroQoL 5-dimensions 5-level (EQ-5D-5L) questionnaire is a standardised measure of health outcome that has been used extensively in patients with stroke.[21, 25, 74]

Under each heading, please tick the ONE box that best describes your health TODAY.

MOBILITY

I have no problems in walking about ❑
I have slight problems in walking about ❑
I have moderate problems in walking about ❑
I have severe problems in walking about ❑
I am unable to walk about ❑

SELF-CARE

I have no problems washing or dressing myself ❑
I have slight problems washing or dressing myself ❑
I have moderate problems washing or dressing myself ❑
I have severe problems washing or dressing myself ❑
I am unable to wash or dress myself ❑

USUAL ACTIVITIES *(e.g. work, study, housework, family or leisure activities)*

I have no problems doing my usual activities ❑
I have slight problems doing my usual activities ❑
I have moderate problems doing my usual activities ❑
I have severe problems doing my usual activities ❑
I am unable to do my usual activities ❑

PAIN / DISCOMFORT

I have no pain or discomfort ❑
I have slight pain or discomfort ❑
I have moderate pain or discomfort ❑
I have severe pain or discomfort ❑

I have extreme pain or discomfort ❑

ANXIETY / DEPRESSION

I am not anxious or depressed ❑

I am slightly anxious or depressed ❑

I am moderately anxious or depressed ❑

I am severely anxious or depressed ❑

I am extremely anxious or depressed ❑

We would like to know how good or bad your health is TODAY.

This scale is numbered from 0 to 100.

100 means the best health you can imagine.
0 means the worst health you can imagine.

Mark an X on the scale to indicate how your health is TODAY.

Now, please write the number you marked on the scale in the box below.

YOUR HEALTH TODAY = [ ]

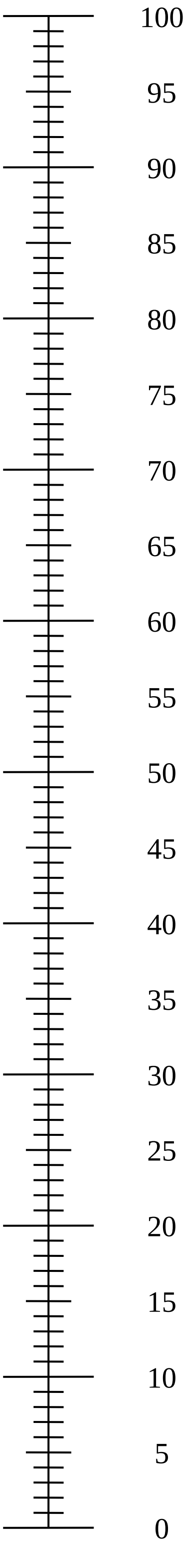

Appendix 7 – Known side effects of the study medication

The side effects below are based on the SmPCs of the IMPs

Metoclopramide
Very common (≥1/10): somnolence
Common (>1/100 - <1/10): extrapyramidal disorders (parkinsonism, akathisia); asthenia; reduced energy levels; depression; hypotension; diarrhea.
Uncommon (>1/1000 - <1/100): bradycardia; amenorrhea; hyperprolactinaemia; dystonia; dyskinesia; hallucinations; hypersensitivity; depressed level of consciousness.
Rare (>1/10.000 - <1/1000): galactorrhoea; convulsions; confusional state, anxiety, restlessness, dizziness.
Very rare (<1/10.000): constipation; swelling of the tongue; .
Not known: methaemoglobinaemia; sulfhaemoglobinaemia; cardiac conduction disturbances (cardiac arrest, atrioventricular block, QT prolongation; Torsade de Pointes); gynaectomastia; anaphylactic reaction; malignant neuroleptic syndrome; tardive dyskinesia; dyspnea; suicidal ideation; acute hypertension in patients with phaeochromocytoma.
In the case of intravenous administration: pain or discomfort at infusion site; phlebitis.

Ceftriaxone
Common (>1/100 - <1/10): diarrhea, nausea, vomiting, stomatitis, glossitis; anemia, haemolytic anemia, granulocytopenia, leucopenia, neutropenia, thrombocytopenia and eosinophilia; skin rashes (maculopapular rash or exanthema, urticarial), pruritus, edema.
Uncommon (>1/1000 - <1/100): allergic reaction.
Rare (>1/10.000 - <1/1000): pain or discomfort at infusion site; phlebitis; abdominal pain; drug fever; shivering; bronchospasm; headache, vertigo, dizziness; glycosuria, oliguria, haematuria, increase in serum creatinine; hepatitis, cholestatic jaundice, increase in liver enzymes; acute renal tubular necrosis; prolongation prothrombin time.
Very rare (<1/10.000): severe cutaneous adverse reactions (erythema multiforme, Stevens Johnson Syndrome and Lyell's Syndrome/toxic epidermal necrolysis); coagulation disorders; very low number of white blood cells; pancreatitis; renal precipitate formation.
Unknown: agranulocytosis; immune mediated anemia; convulsions; fatal haemolysis; bleeding; bruising; precipitates of calcium ceftriaxone in the gall bladder.
Remaining: Overgrowth syndrome of *Clostridium difficile*. The risk of developing C. Difficile overgrowth was very low in previous studies with ceftriaxone (risk <1%). Infection with Clostridium difficile during treatment with ceftriaxone may cause pseudomembranous colitis. Therefore, the possibility of the disease should be considered in patients who present with diarrhea following antibacterial agent use. Superinfection caused by other microorganisms non-susceptible to ceftriaxone such as yeasts, fungi (mycosis of the genital tract) or other resistant microorganisms may also develop.

Paracetamol
Rare (>1/10.000 - <1/1000): elevated transaminases; hypersensitivity; skin rash; hypotension; malaise.
Very rare (<1/10.000): allergic reaction; serious skin reaction; trombocytopenia, neutropenia, pancytopenia, leukopenia, agranulocytosis,
Unknown: tachycardia; pruritus.

In the case of intravenous administration: pain or discomfort at infusion site; phlebitis.

Appendix 8 – common (potentially) serious complications after stroke

Based on: [31, 32, 75, 76]

Cardiac

Angina
Arrhythmia
Atrial fibrillation
Angina pectoris
Bradycardia
Cardiac arrest
Cardiomyopathy
Heart failure
Myocardial infarction
Tachycardia

Central nervous system

Brain oedema
Cerebral herniation
Cerebellar herniation
Delirium
Depression
Epileptic seizure
Haemorrhagic transformation of the infarct
Headache
Haematoma expansion
Hydrocephalus
Intraventricular extension of haemorrhage
Increased intracranial pressure
Progressive stroke / Stroke in evolution
Recurrent ischaemic stroke
Recurrent intracerebral haemorrhage
Recurrent stroke
Retinal ischaemia
Sleep disorder
Status epilepticus
Transient ischaemic attack
Transient monocular blindness

Gastro-intestinal

Constipation
Dysphagia
Faecal incontinence
Gastro-intestinal haemorrhage

Ileus
Melena
Mucosal irritation
Nausea
Rectal haemorrhage
Stress ulcer
Vomiting

General / other

Anaemia
Arterial hypertension
Arterial hypotension
Deep vein thrombosis
Dehydration
Fall (and consequences)
Fatigue
Fever
Haematuria
Hip fracture
Hyperglycaemia
Infections
Pain
Pressure sore
Renal failure
Sepsis
Syncope
Undernutrition
Urinary incontinence
Urinary tract infection

Pulmonary

Aspiration
Bronchitis
Central periodic breathing
Chronic obstructive pulmonary disease
Dyspnoea
Obstructive sleep apnoea
Oxygen desaturation
Pneumonia
Pulmonary embolism
Pulmonary oedema
Respiratory failure / arrest
Respiratory tract infection

Appendix 9 – protocol signature sheet local principal investigator

Protocol version 4.1, dated 16 December 2020

I agree to the conditions relating to this study as set out in the above named protocol. I fully understand that any changes instituted by the Investigator(s) without previous discussion with the appropriate sponsor personnel would constitute a violation of the protocol, including any ancillary studies or procedures performed on study subjects (other than those procedures necessary for the well–being of the subjects).

I acknowledge that I have read the above-named protocol and agree to carry out all of its terms in accordance with the applicable regulations and law, to follow ICH GCP guidelines for good clinical practice, to obtain the required regulatory approvals prior to implementation, to allow direct access to source documents where required by rules and regulation, and agree to inspection by auditors from regulatory authorities, as required by ICH GCP. I will assure that the investigational product(s) will be used only as described in the above-named protocol.

To be signed by Principal Investigator and filed in the local ISF.

Site Name:	
Full name Principal Investigator:	
Signature:	
Date:	

16. SUMMARY OF CHANGES

16.1. AMENDMENT 1

Version number: 3.0
Version date: 19 January 2017

Section number: N/A	*Section title:* Signature page	*Page number:* 2
Old text	N/A	
New text	[Text not copied here]. A signature page with details of the representative of the Study Sponsor and the Chief Investigator has been added.	
Rationale for change	Comply with GCP.	
Section number: N/A	*Section title:* Synopsis	*Page number:* 10
Old text	Patients will be randomly allocated in a 2*2*2 factorial design to any combination of open-label oral, rectal, or intravenous metoclopramide (10 mg thrice daily), intravenous ceftriaxone (2000 mg once daily), oral, rectal, or intravenous paracetamol (1000 mg four times daily), or usual care, started within 12 hours after symptom onset and continued for 4 days or until complete recovery or discharge from hospital, if earlier.	
New text	Patients will be randomly allocated in a 2*2*2 factorial design to any combination of open-label oral, rectal, or intravenous metoclopramide (10 mg thrice daily), intravenous ceftriaxone (2000 mg once daily), oral, rectal, or intravenous paracetamol (1000 mg four times daily), or usual care, started within 12 hours after symptom onset and continued for 4 days or until complete recovery or discharge from hospital, if earlier. In patients with moderate to severe renal impairment or with severe hepatic impairment, the dose of metoclopramide is reduced to 5 mg thrice daily, and in patients with end-stage renal disease to 2.5 mg thrice daily.	
Rationale for change	The dose of metoclopramide is now in line with the dose recommended in the most recent SmPCs.	
Section number: 5.2	*Section title:* Treatment allocation	*Page number:* 27
Old text	Patients will be randomly allocated in a 2*2*2 factorial design to any combination of open-label oral, rectal, or intravenous metoclopramide (10 mg thrice daily), intravenous ceftriaxone (2000 mg once daily), oral, rectal, or intravenous paracetamol (1000 mg four times daily), or usual care, started within 12 hours after symptom onset and continued for 4 days or until complete recovery or discharge from hospital, if earlier.	
New text	Patients will be randomly allocated in a 2*2*2 factorial design to any combination of open-label oral, rectal, or intravenous metoclopramide (10 mg thrice daily), intravenous ceftriaxone (2000 mg once daily), oral, rectal, or intravenous paracetamol (1000 mg four times daily), or usual care, started within 12	

	hours after symptom onset and continued for 4 days or until complete recovery or discharge from hospital, if earlier. In patients with moderate to severe renal impairment or with severe hepatic impairment, the dose of metoclopramide is reduced to 5 mg thrice daily, and in patients with end-stage renal disease to 2.5 mg thrice daily.	
Rationale for change	The dose of metoclopramide is now in line with the dose recommended in the most recent SmPCs, and the dose of paracetamol with the maximum daily dose recommended in Italy.	
Section number: 5.3.1	Section title: Metoclopramide	Page number: 28
Old text	For details we refer to the relevant SmPCs.	
New text	For details we refer to the relevant SmPCs. Note that for this off-patent drug, a wide range of SmPCs is available, with subtle differences. The SmPCs submitted for review by Competent Authorities and Research Ethics Committees should therefore be considered examples. Annual review of the SmPCs will only lead to amendments to the study protocol in case of new clinical findings that are of direct relevance to the study population.	
Rationale for change	Clarification that there are several versions of the SmPCs available, with subtle differences.	
Section number: 5.3.1.	*Section title:* Metoclopramide	*Page number:* 29
Old text	Metoclopramide will be administered as 10 mg tablets, 10 mg suppositories, or 10 mg intravenous infusions in a daily dose of 3 times 10 mg for a period of 4 days or until discharge, if earlier.	
New text	Metoclopramide will be administered as 10 mg tablets, 10 mg suppositories, or 10 mg intravenous infusions in a daily dose of 3 times 10 mg for a period of 4 days or until discharge, if earlier. In patients with end-stage renal disease (creatinine clearance ≤ 15 ml/min), the daily dose should be reduced to 3 times 2.5 mg; in patients with moderate to severe renal impairment (creatinine clearance 15 to 60 ml/min), the dose should be reduced to 3 times 5 mg. In patients with severe hepatic impairment (liver cirrhosis), the dose should be reduced to 3 times 5 mg.	
Rationale for change	The dose of metoclopramide is now in line with the dose recommended in the most recent SmPCs.	
Section number: 5.3.1.	*Section title:* Metoclopramide	*Page number:* 29 and 30
Old text	N/A	
New text	**Interaction with other medicinal products**<br><br>*General*<br>Due to the prokinetic effect of metoclopramide, the absorption of certain drugs may be modified.<br><br>*Anticholinergics and morphine derivatives*	

	Anticholinergics and morphine derivatives may have both a mutual antagonism with metoclopramide on the digestive tract motility.<br><br>*Central nervous system depressants (morphine derivatives, anxiolytics, sedative H1 antihistamines, sedative antidepressants, barbiturates, clonidine and related)*<br>Sedative effects of central nervous system depressants and metoclopramide are potentiated.<br><br>*Neuroleptics*<br>Metoclopramide may have an additive effect with other neuroleptics on the occurrence of extrapyramidal disorders.<br><br>*Serotonergic drugs*<br>The use of metoclopramide with serotonergic drugs such as SSRIs may increase the risk of serotonin syndrome.<br><br>*Digoxin*<br>Metoclopramide may decrease digoxin bioavailability. Careful monitoring of digoxin plasma concentration is required.<br><br>*Cyclosporine*<br>Metoclopramide increases cyclosporine bioavailability (Cmax by 46% and exposure by 22%). Careful monitoring of cyclosporine plasma concentration is required. The clinical consequence is uncertain.<br><br>*Mivacurium and suxamethonium*<br>Metoclopramide injection may prolong the duration of neuromuscular block (through inhibition of plasma cholinesterase).<br><br>*Strong CYP2D6 inhibitors*<br>Metoclopramide exposure levels are increased when co-administered with strong CYP2D6 inhibitors such as fluoxetine and paroxetine. Although the clinical significance is uncertain, patients should be monitored for adverse reactions.	
Rationale for change	A section on interactions with other medicinal products has been added to increase patient's safety. This section has been copied from the SmPC.	
Section number: 5.3.2	Section title: Ceftriaxone	Page number: 30
Old text	For details we refer to the relevant SmPCs.	
New text	For details we refer to the relevant SmPCs. Note that for this off-patent drug, a wide range of SmPCs is available, with subtle differences. The SmPC submitted for review by Competent Authorities and Research Ethics Committees should therefore	

	be considered an example. Annual review of the SmPC will only lead to amendments to the study protocol in case of new clinical findings that are of direct relevance to the study population.	
Rationale for change	Clarification that there are several versions of the SmPCs available, with subtle differences.	
Section number: 5.3.3.	*Section title:* Paracetamol	*Page number:* 31
Old text	Paracetamol will be administered as 500 mg or 1000 mg tablets, 1000 mg suppositories, or 1000 mg intravenous infusions in a daily dose of 4 times 1000 mg for a period of 4 days or until discharge, if earlier. Paracetamol will only be given intravenously if oral or rectal administration is impossible, impractical, or refused by the patient.	
New text	Paracetamol will be administered as 500 mg or 1000 mg tablets, 1000 mg suppositories, or 1000 mg intravenous infusions in a daily dose of 4 times 1000 mg for a period of 4 days or until discharge, if earlier. In Italy and Norway, the daily dose of 4000 mg daily is higher than the maximum daily dose of 3000 mg recommended in the SmPCs in these countries, but a daily dose of 4000 mg has been approved by the Italian and Norwegian competent authorities for use in PRECIOUS. Paracetamol will only be given intravenously if oral or rectal administration is impossible, impractical, or refused by the patient.	
Rationale for change	Information to the investigators that the dose of paracetamol used in PRECIOUS is above the maximum daily dose recommended in Italy and Norway.	
Section number: 5.3.3	Section title: Paracetamol	Page number: 31
Old text	For details we refer to the relevant SmPCs.	
New text	For details we refer to the relevant SmPCs. Note that for this off-patent drug, a wide range of SmPCs is available, with subtle differences. The SmPCs submitted for review by Competent Authorities and Research Ethics Committees should therefore be considered examples. Annual review of the SmPC will only lead to amendments to the study protocol in case of new clinical findings that are of direct relevance to the study population.	
Rationale for change	Clarification that there are several versions of the SmPCs available, with subtle differences.	
Section number: 9.2.1	*Section title: Reporting of AEs and SAEs*	*Page number: 46*
Old text	In order to prevent redundant data entry, outcome events and endpoints of the clinical trial (e.g. death and infection) are not considered as AEs and will be documented separately as outcome events in the eCRF.	
New text	In order to prevent redundant data entry, outcome events and endpoints of the clinical trial (e.g. death and infection) will be documented only as outcome events in the eCRF and not as AEs.	

Rationale for change	To clarify the reporting of AEs to the investigators.	
Section number: Appendix 7	*Section title:* Known side effects of the study medication	*Page number:* 70
Old text	**Metoclopramide**<br>Very common (≥1/10): somnolence<br>Common (>1/100 - <1/10): extrapyramidal disorders (parkinsonism, akathisia); asthenia; reduced energy levels; depression; hypotension; diarrhea.<br>Uncommon (>1/1000 - <1/100): bradycardia; amenorrhea; hyperprolactinaemia; dystonia; dyskinesia; tardive dyskinesia; hallucinations; hypersensitivity.<br>Rare (>1/10.000 - <1/1000): galactorrhoea; convulsions; confusional state, anxiety, restlessness, dizziness.<br>Very rare (<1/10.000): cardiac conduction disturbances (cardiac arrest, atrioventricular block, QT prolongation); constipation; swelling of the tongue; anaphylactic reaction; methaemoglobinaemia; sulfhaemoglobinaemia; gynaectomastia; malignant neuroleptic syndrome.<br>In the case of intravenous administration: pain or discomfort at infusion site; phlebitis.	
New text	**Metoclopramide**<br>Very common (≥1/10): somnolence<br>Common (>1/100 - <1/10): extrapyramidal disorders (parkinsonism, akathisia); asthenia; reduced energy levels; depression; hypotension; diarrhea.<br>Uncommon (>1/1000 - <1/100): bradycardia; amenorrhea; hyperprolactinaemia; dystonia; dyskinesia; hallucinations; hypersensitivity; depressed level of consciousness.<br>Rare (>1/10.000 - <1/1000): galactorrhoea; convulsions; confusional state, anxiety, restlessness, dizziness.<br>Very rare (<1/10.000): constipation; swelling of the tongue; .<br>Not known: methaemoglobinaemia; sulfhaemoglobinaemia; cardiac conduction disturbances (cardiac arrest, atrioventricular block, QT prolongation; Torsade de Pointes); gynaectomastia; anaphylactic reaction; malignant neuroleptic syndrome; tardive dyskinesia; acute hypertension in patients with phaeochromocytoma<br>In the case of intravenous administration: pain or discomfort at infusion site; phlebitis.	
Rationale for change	The list of undesirable effects is now in line with the most recent versions of the SmPCs of metoclopramide. Please note that only items that have been added are indicated in red. For some other adverse reactions, the frequency has been adapted.	
Section number: Appendix 7	*Section title:* Known side effects of the study medication	*Page number:* 70

Old text	**Ceftriaxone**<br>Common (>1/100 - <1/10): diarrhea, nausea, vomiting, stomatitis, glossitis; anemia, haemolytic anemia, granulocytopenia, leucopenia, neutropenia, thrombocytopenia and eosinophilia; skin rashes (maculopapular rash or exanthema, urticarial), pruritus, edema.<br>Uncommon (>1/1000 - <1/100): allergic reaction.<br>Rare (>1/10.000 - <1/1000): pain or discomfort at infusion site; phlebitis; abdominal pain; fever; shivers; headache, vertigo, dizziness; glycosuria, oliguria, haematuria, increase in serum creatinine; hepatitis, cholestatic jaundice, increase in liver enzymes.<br>Very rare (<1/10.000): serious skin rash with blisters; coagulation disorders; very low number of white blood cells; pancreatitis.<br>Unknown: agranulocytosis; immune mediated anemia; convulsions.<br>Remaining: Overgrowth syndrome of Clostridium difficile. The risk of developing C. Difficile overgrowth was very low in previous studies with ceftriaxone (risk <1%).
New text	**Ceftriaxone**<br>Common (>1/100 - <1/10): diarrhea, nausea, vomiting, stomatitis, glossitis; anemia, haemolytic anemia, granulocytopenia, leucopenia, neutropenia, thrombocytopenia and eosinophilia; skin rashes (maculopapular rash or exanthema, urticarial), pruritus, edema.<br>Uncommon (>1/1000 - <1/100): allergic reaction.<br>Rare (>1/10.000 - <1/1000): pain or discomfort at infusion site; phlebitis; abdominal pain; drug fever; shivering; bronchospasm; headache, vertigo, dizziness; glycosuria, oliguria, haematuria, increase in serum creatinine; hepatitis, cholestatic jaundice, increase in liver enzymes; acute renal tubular necrosis; prolongation prothrombin time.<br>Very rare (<1/10.000): severe cutaneous adverse reactions (erythema multiforme, Stevens Johnson Syndrome and Lyell's Syndrome/toxic epidermal necrolysis); coagulation disorders; very low number of white blood cells; pancreatitis; renal precipitate formation.<br>Unknown: agranulocytosis; immune mediated anemia; convulsions; fatal haemolysis; bleeding; bruising; precipitates of calcium ceftriaxone in the gall bladder.<br>Remaining: Overgrowth syndrome of Clostridium difficile. The risk of developing C. Difficile overgrowth was very low in previous studies with ceftriaxone (risk <1%). Infection with Clostridium difficile during treatment with ceftriaxone may cause pseudomembranous colitis. Therefore, the possibility of the disease should be considered in patients who present with diarrhea following antibacterial agent use. Superinfection

	caused by other microorganisms non-susceptible to ceftriaxone such as yeasts, fungi (mycosis of the genital tract) or other resistant microorganisms may also develop.	
Rationale for change	The list of undesirable effects is now in line with the most recent versions of the SmPCs of ceftriaxone. Please note that only items that have been added are indicated in red. For some other adverse reactions, the frequency has been adapted.	
Section number: Appendix 9	*Section title:* protocol signature sheet local principal investigator	*Page number:* 74
Old text	N/A	
New text	Protocol version 3.0, dated 19 January 2017<br><br>I agree to the conditions relating to this study as set out in the above named protocol. I fully understand that any changes instituted by the Investigator(s) without previous discussion with the appropriate sponsor personnel would constitute a violation of the protocol, including any ancillary studies or procedures performed on study subjects (other than those procedures necessary for the well–being of the subjects).<br><br>I acknowledge that I have read the above-named protocol and agree to carry out all of its terms in accordance with the applicable regulations and law, to follow ICH GCP guidelines for good clinical practice, to obtain the required regulatory approvals prior to implementation, to allow direct access to source documents where required by rules and regulation, and agree to inspection by auditors from regulatory authorities, as required by ICH GCP. I will assure that the investigational product(s) will be used only as described in the above-named protocol.<br><br>To be signed by Principal Investigator and filed in the local ISF.<br><br>Site Name:<br><br>Full name Principal Investigator:<br><br>Signature:<br><br>Date:	
Rationale for change	Comply with GCP.	

16.2. AMENDMENT 2

Version number: 3.1

Version date: 28 June 2017

Section number: 5.3.1	*Section title:* Metoclopramide	*Page number:* 30
Old text	Study medication is defined by active substance and therefore no labelling is required.	
New text	Study medication is defined by active substance and will be labelled according to national rules and regulations.	
Rationale for change	Requirement of Study Sponsor.	
Section number: 5.3.2	*Section title:* Ceftriaxone	*Page number:* 31
Old text	Study medication is defined by active substance and therefore no labelling is required.	
New text	Study medication is defined by active substance and will be labelled according to national rules and regulations.	
Rationale for change	Requirement of Study Sponsor.	
Section number: 5.3.3	*Section title:* Paracetamol	*Page number:* 32
Old text	Study medication is defined by active substance and therefore no labelling is required	
New text	Study medication is defined by active substance and will be labelled according to national rules and regulations.	
Rationale for change	Requirement of Study Sponsor.	

Administrative changes

Minor changes involving grammar, wordsmithing, punctuation, and other editorial changes have been made throughout the document. These minor changes are not recorded in the summary of changes.

16.3. AMENDMENT 3

Version number: 4.0
Version date: 13 December 2017

Section number: N/A	*Section title:* Synopsis	*Page number:* 9
Old text	the possibility to start treatment within 12 hours of symptom onset	
New text	the possibility to start treatment within 24 hours of symptom onset	
Rationale for change	The inclusion window of the trial is extended from 12 to 24 hours to increase the recruitment rate in the trial. Extending the time period between stroke onset and the start of the treatment will not affect the potential of the trial to prevent complications, and to improve functional outcome. The peak incidence of fever and infections is later than 24 hours after stroke onset.[1-3] The presence of fever in the first days after stroke is associated with a higher risk of a poor outcome than the presence of fever on admission in the first hours after stroke.[4,5] Starting treatment within 24 hours rather than 12 hours therefore still has the potential to prevent complications which are associated with a poor outcome.<br><br>In many of the participating centres, patient inclusion is limited to working hours, and with a time window for inclusion of 12 hours patients admitted outside working hours are often missed. An extension of this time window to 24 hours will greatly increase to possibility to include patients in the trial.<br><br>References<br>1. Saini M, Saqqur M, Kamruzzaman A, Lees KR, Shuaib A. Effect of hyperthermia on prognosis after acute ischemic stroke. Stroke 2009;40(9):3051-9.<br>2. Karaszewski B, Thomas RG, Dennis MS, Wardlaw JM. Temporal profile of body temperature in acute ischemic stroke: relation to stroke severity and outcome. BMC Neurol 2012;18(12):123.<br>3. Vermeij FH, Scholte op Reimer WJM, de Man P, van Oostenbrugge RJ, Franke CL, de Jonge G, et al. Stroke-associated infection is an independent risk factor for poor outcome after acute ischemic stroke: data from the Netherlands Stroke Survey. Cerebrovasc Dis 2009;27:465-71.<br>4. den Hertog HM, van der Worp HB, van Gemert HM, Algra A, Kappelle LJ, van Gijn J, et al. An early rise in body temperature is related to unfavorable outcome after stroke: data from the PAIS study. J Neurol 2011;258(2):302-7.<br>5. Geurts M, Scheijmans FE, van Seeters T, Biessels GJ, Kappelle LJ, Velthuis BK, et al. Temporal profile of body temperature in acute ischemic stroke: relation to infarct size and outcome. BMC Neurol 2016;16(1):233.	
Section number: N/A	*Section title:* Synopsis	*Page number:* 9, 10
Old text	Exclusion criteria	

	1. Active infection requiring antibiotic treatment, as judged by the treating physician;<br>2. Clinical indication for one or more of the drugs tested in this patient;<br>3. Pre-stroke score on the mRS ≥4<br>4. Death appearing imminent at the time of assessment.<br><br>In addition, patients will be excluded from participation in the trial treatment strata for any of the following reasons:<br>**For the ceftriaxone stratum:**<br>1. Known hypersensitivity to beta-lactam antibiotics;<br>**For the paracetamol stratum:**<br>1. Known hypersensitivity to paracetamol or any of the excipients;<br>2. Known severe hepatic insufficiency;<br>3. Chronic alcoholism.<br>**For the metoclopramide stratum:**<br>1. Hypersensitivity to metoclopramide or to any of the excipients;<br>2. Gastrointestinal haemorrhage, mechanical obstruction or gastro-intestinal perforation for which the stimulation of gastrointestinal motility constitutes a risk;<br>3. Confirmed or suspected pheochromocytoma;<br>4. History of neuroleptic or metoclopramide-induced tardive dyskinesia;<br>5. Epilepsy;<br>6. Parkinson's disease;<br>7. Use of levodopa or dopaminergic agonists;<br>8. Known history of methaemoglobinaemia with metoclopramide or of NADH cytochrome-b5 deficiency.
New text	Exclusion criteria<br><br>1. Active infection requiring antibiotic treatment, as judged by the treating physician;<br>2. Pre-stroke score on the mRS ≥4<br>3. Death appearing imminent at the time of assessment.<br><br>In addition, patients will be excluded from participation in the trial treatment strata for any of the following reasons:<br>**For the ceftriaxone stratum:**<br>1. Known hypersensitivity to beta-lactam antibiotics;<br>2. Clinical indication for antibiotic treatment. Use of an antibiotic before screening is not an exclusion criterion.<br>**For the paracetamol stratum:**<br>1. Known hypersensitivity to paracetamol or any of the excipients;<br>2. Known severe hepatic insufficiency;<br>3. Chronic alcoholism.

	4. Clinical indication for the use of paracetamol. Incidental use of paracetamol before screening is not an exclusion criterion.<br><br>**For the metoclopramide stratum:**<br>1. Hypersensitivity to metoclopramide or to any of the excipients;<br>2. Gastrointestinal haemorrhage, mechanical obstruction or gastro-intestinal perforation for which the stimulation of gastrointestinal motility constitutes a risk;<br>3. Confirmed or suspected pheochromocytoma;<br>4. History of neuroleptic or metoclopramide-induced tardive dyskinesia;<br>5. Epilepsy;<br>6. Parkinson's disease;<br>7. Use of levodopa or dopaminergic agonists;<br>8. Known history of methaemoglobinaemia with metoclopramide or of NADH cytochrome-b5 deficiency.<br>9. Clinical indication for the use of metoclopramide. Incidental use of metoclopramide before screening is not an exclusion criterion.	
Rationale for change	The original exclusion criterion '*Clinical indication for one or more of the drugs tested in this patient*' proved to give rise to confusion. It has now been clarified that 1. Patients who have an indication for one of the study drugs may still be included and randomised only for the other two stratums. The stratum of the drug for which there is a clinical indication will be censored. 2. Patients who have received one or more doses of one of the study drugs before screening may still be included if there is no clinical indication to continue this particular drug.	
Section number: N/A	*Section title:* Synopsis	*Page number:* 10
Old text	the possibility to start treatment within 12 hours of symptom onset	
New text	the possibility to start treatment within 24 hours of symptom onset	
Rationale for change	See "Rationale for change" above	
Section number: 1.0	*Section title:* Introduction and rationale	*Page number:* 19
Old text	the possibility to start treatment within 12 hours of symptom onset	
New text	the possibility to start treatment within 24 hours of symptom onset	
Rationale for change	See "Rationale for change" above.	
Section number: 1.0	*Section title:* Introduction and rationale	*Page number:* 22
Old text	the possibility to start treatment within 12 hours of symptom onset	
New text	the possibility to start treatment within 24 hours of symptom onset	

Rationale for change	See "Rationale for change" above	
Section number: 4.2	*Section title:* Inclusion criteria	*Page number:* 25
Old text	the possibility to start treatment within 12 hours of symptom onset	
New text	the possibility to start treatment within 24 hours of symptom onset	
Rationale for change	See "Rationale for change" above	
Section number: 4.3	*Section title:* Exclusion criteria	*Page number:* 26
Old text	Identical to changes in the "synopsis" above (page number 9 and 10)	
New text	Identical to changes in the "synopsis" above (page number 9 and 10)	
Rationale for change	See "Rationale for change" above	
Section number: 5.1.2	*Section title:* Hospital phase	*Page number:* 27
Old text	the possibility to start treatment within 12 hours of symptom onset	
New text	the possibility to start treatment within 24 hours of symptom onset	
Rationale for change	See "Rationale for change" above	
Section number: 5.2	*Section title:* Treatment allocation	*Page number:* 27
Old text	the possibility to start treatment within 12 hours of symptom onset	
New text	the possibility to start treatment within 24 hours of symptom onset	
Rationale for change	See "Rationale for change" above	
Section number: 7.5	*Section title:* Subgroup analyses	*Page number:* 38
Old text	Time to treatment (<6, ≥6 hours);	
New text	Time to treatment (<6, ≥6 hours, <12 hours, ≥12 hours);	
Rationale for change	Because of the extension of the inclusion time frame, we have added a new subgroup analysis to assess if the treatment effect differs between patients included in the short (<12 hours) and extended (>12 hours) inclusion period.	
Section number: 12.3	*Section title:* Informed consent	*Page number:* 51
Old text	the possibility to start treatment within 12 hours of symptom onset	
New text	the possibility to start treatment within 24 hours of symptom onset	
Rationale for change	See "Rationale for change" above	
Section number: Appendix 7	*Section title:* Known side effects of the study medication	*Page number:* 70
Old text	**Metoclopramide**	

	Very common (≥1/10): somnolence<br>Common (>1/100 - <1/10): extrapyramidal disorders (parkinsonism, akathisia); asthenia; reduced energy levels; depression; hypotension; diarrhea.<br>Uncommon (>1/1000 - <1/100): bradycardia; amenorrhea; hyperprolactinaemia; dystonia; dyskinesia; tardive dyskinesia; hallucinations; hypersensitivity.<br>Rare (>1/10.000 - <1/1000): galactorrhoea; convulsions; confusional state, anxiety, restlessness, dizziness.<br>Very rare (<1/10.000): cardiac conduction disturbances (cardiac arrest, atrioventricular block, QT prolongation); constipation; swelling of the tongue; anaphylactic reaction; methaemoglobinaemia; sulfhaemoglobinaemia; gynaectomastia; malignant neuroleptic syndrome.<br>In the case of intravenous administration: pain or discomfort at infusion site; phlebitis.
New text	**Metoclopramide**<br>Very common (≥1/10): somnolence<br>Common (>1/100 - <1/10): extrapyramidal disorders (parkinsonism, akathisia); asthenia; reduced energy levels; depression; hypotension; diarrhea.<br>Uncommon (>1/1000 - <1/100): bradycardia; amenorrhea; hyperprolactinaemia; dystonia; dyskinesia; hallucinations; hypersensitivity; depressed level of consciousness.<br>Rare (>1/10.000 - <1/1000): galactorrhoea; convulsions; confusional state, anxiety, restlessness, dizziness.<br>Very rare (<1/10.000): constipation; swelling of the tongue; .<br>Not known: methaemoglobinaemia; sulfhaemoglobinaemia; cardiac conduction disturbances (cardiac arrest, atrioventricular block, QT prolongation; Torsade de Pointes); gynaectomastia; anaphylactic reaction; malignant neuroleptic syndrome; tardive dyskinesia; dyspnea; suicidal ideation; acute hypertension in patients with phaeochromocytoma<br>In the case of intravenous administration: pain or discomfort at infusion site; phlebitis.
Rationale for change	The list of undesirable effects is updated with relevant changes in the new versions of the SmPCs. Please note that only items that have been added are indicated in red.

Administrative changes

Minor changes involving grammar, wordsmithing, punctuation, and other editorial changes have been made throughout the document. These minor changes are not recorded in the summary of changes.

16.4. AMENDMENT 4

Version number: 4.1
Version date: 16-12-2020

Section number: N/A	*Section title:* Signature page	*Page number:* 2
Old text:	Professor Gabriël J.E. Rinkel	
New text:	Professor Nicolette C. Notermans	
Rationale for change:	New chairman of the department	
Section number: N/A	*Section title:* Synopsis	*Page number:* 11
Old text	Planned schedule: First patient included by May 2016;final follow-up of the last patient by April 2020.	
New text	Planned schedule: First patient included by May 2016; final follow-up of the last patient by November 2022.	
Rationale for change	Extension of the trial duration	
Section number: 3	*Section title:* Study design	*Page number:* 25
Old text	Patients will be recruited in about 80 hospitals in about 9 European countries over a period of about four years	
New text	Patients will be recruited in about 80 hospitals in about 9 European countries over a period of about seven years	
Rationale for change	Extension of the trial duration	

Administrative changes
Minor changes involving grammar, wordsmithing, punctuation, and other editorial changes have been made throughout the document. These minor changes are not recorded in the summary of changes.
